# Supplementary material for: The haplotype-resolved assembly of COL40 a cassava (Manihot esculenta) line with broad-spectrum resistance against viruses causing Cassava brown streak disease unveils a region of highly repeated elements on chromosome 12
Source: G3 (Bethesda). 2025 Apr 16;15(6):jkaf083. doi: 10.1093/g3journal/jkaf083 (PMC12134984; doi:10.1093/g3journal/jkaf083)
Supplement: jkaf083_Supplementary_Data [file jkaf083_supplementary_data.zip › Additional_File_3_G3-2024-405442.pdf]

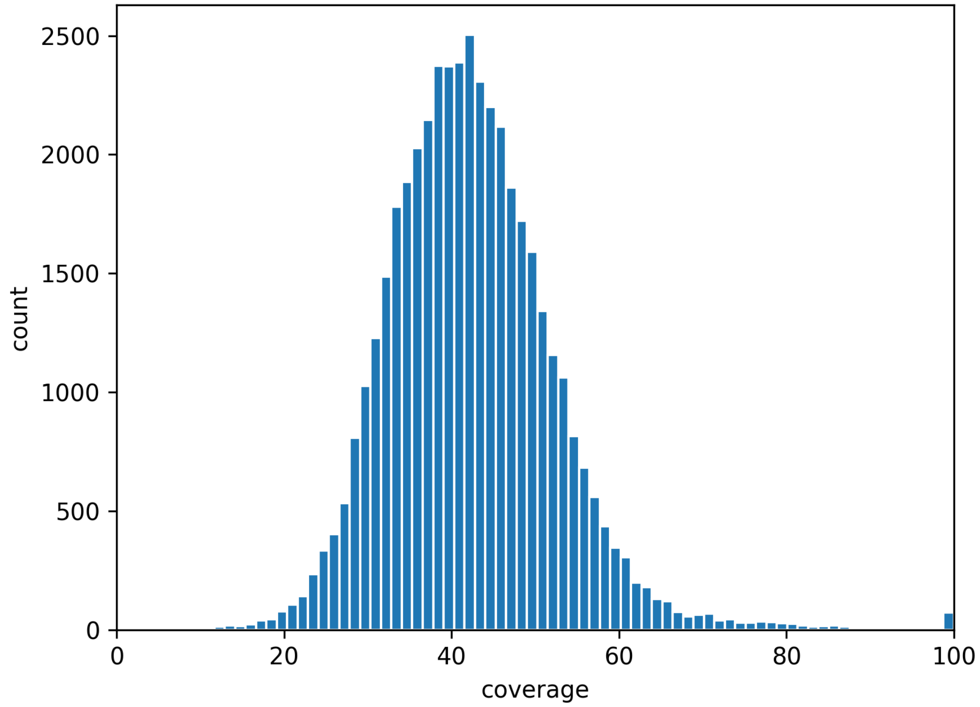


Figure 1: Coverage histogram of mean coverage over 1000 bp on chromosome 1 of haplophase A

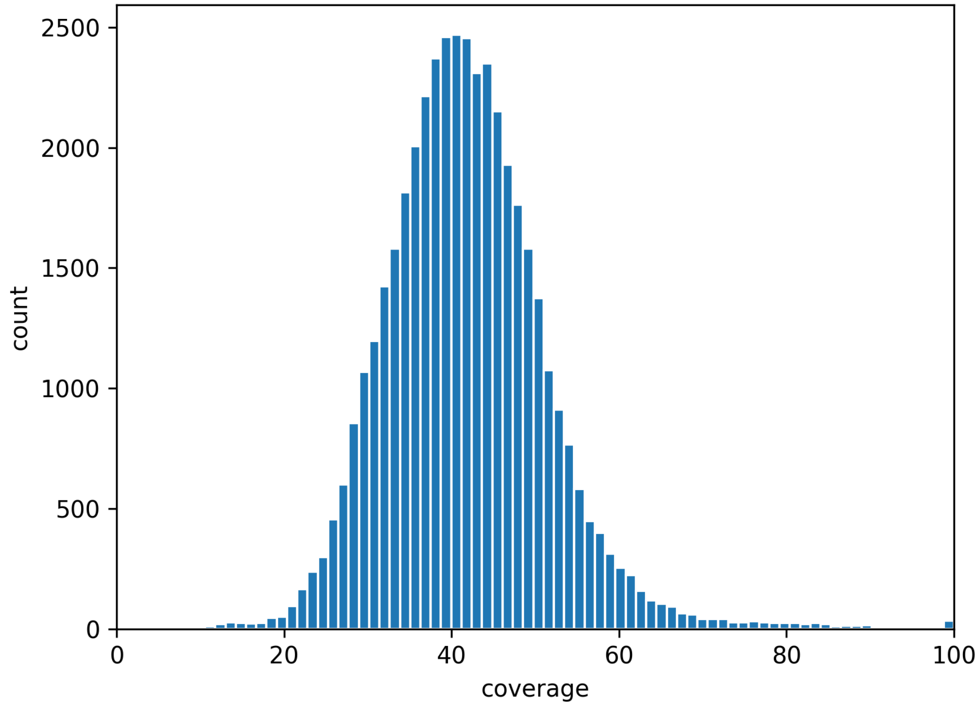


Figure 2: Coverage histogram of mean coverage over 1000 bp on chromosome 2 of haplophase A

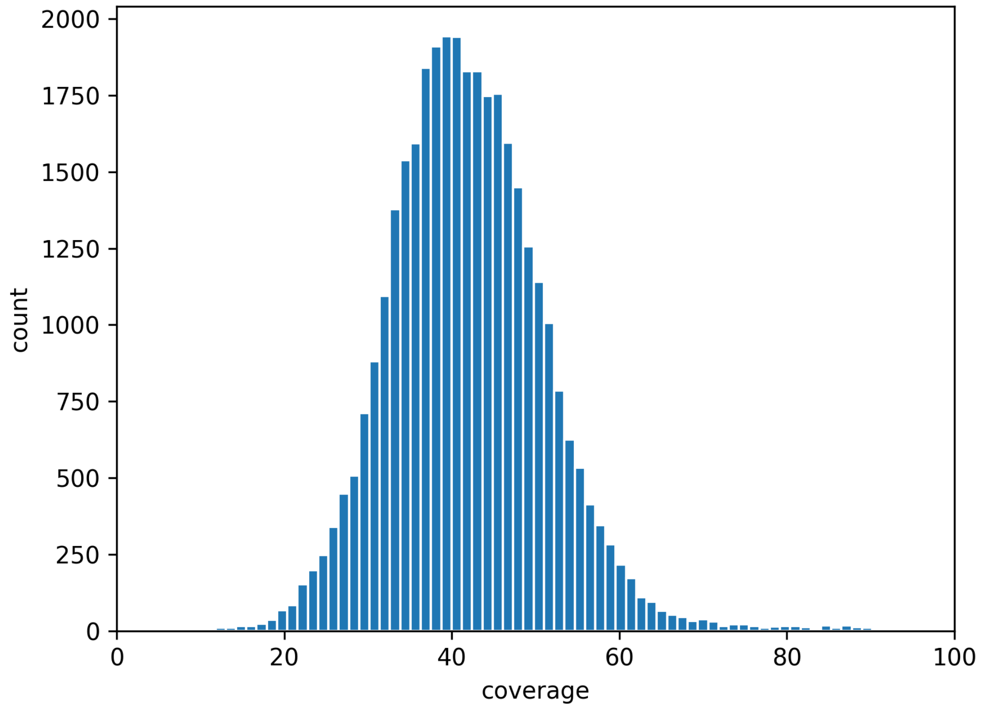


Figure 3: Coverage histogram of mean coverage over 1000 bp on chromosome 3 of haplophase A

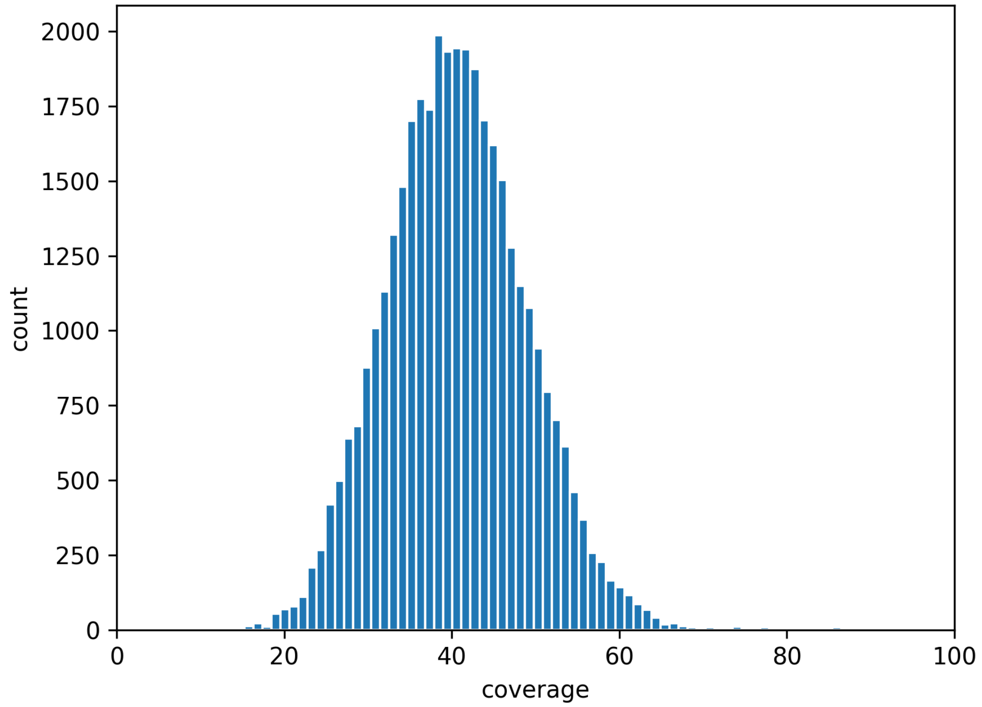


Figure 4: Coverage histogram of mean coverage over 1000 bp on chromosome 4 of haplophase A

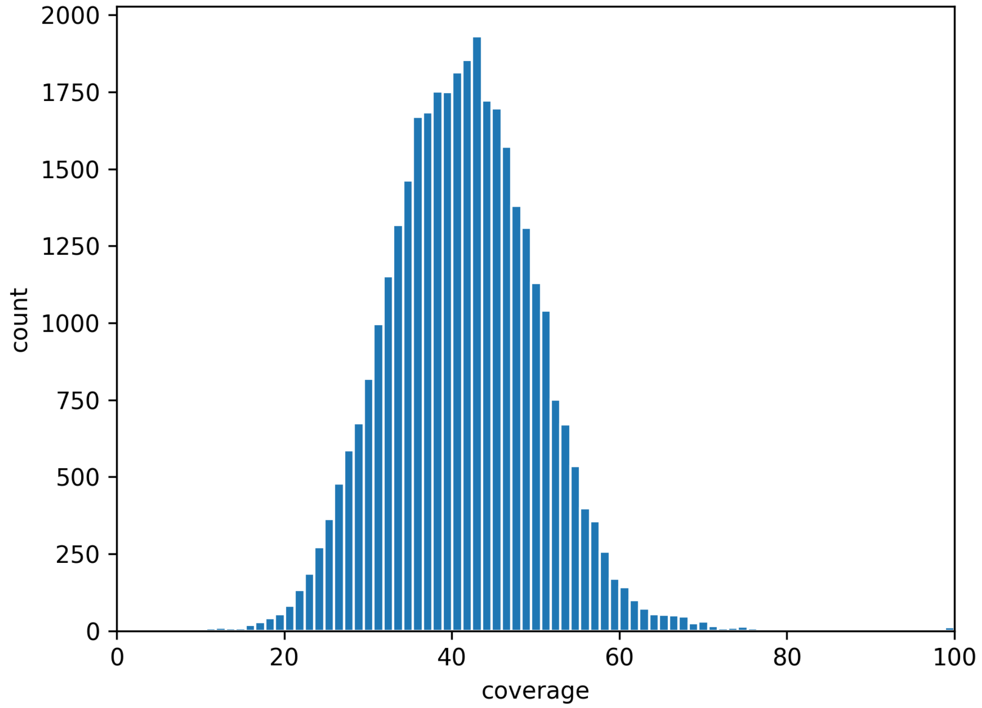


Figure 5: Coverage histogram of mean coverage over 1000 bp on chromosome 5 of haplophase A

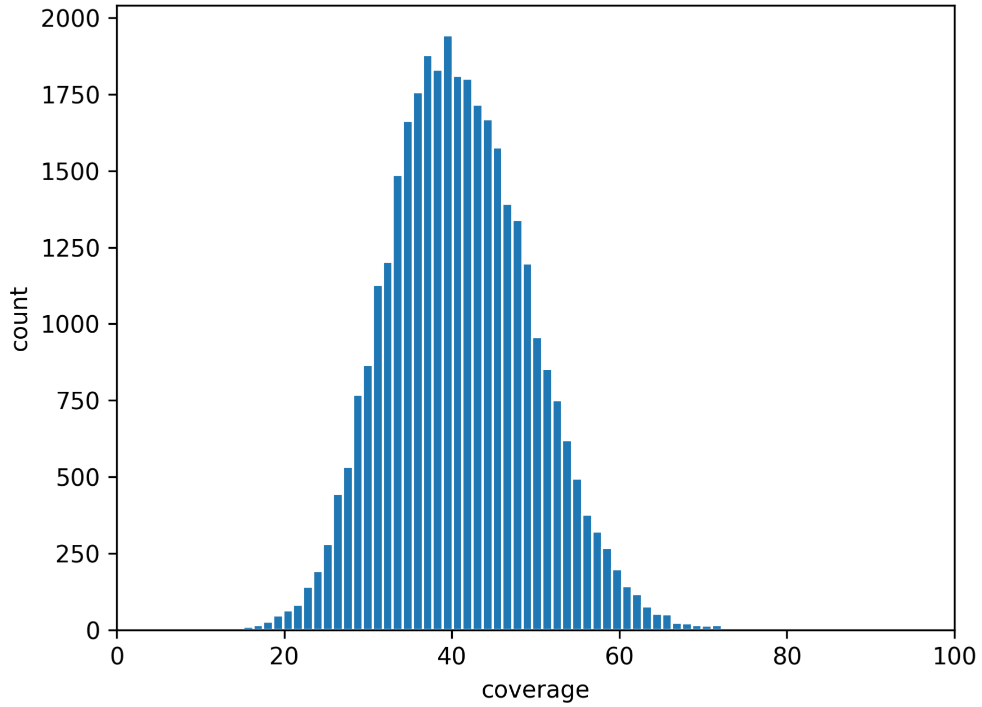


Figure 6: Coverage histogram of mean coverage over 1000 bp on chromosome 6 of haplophase A

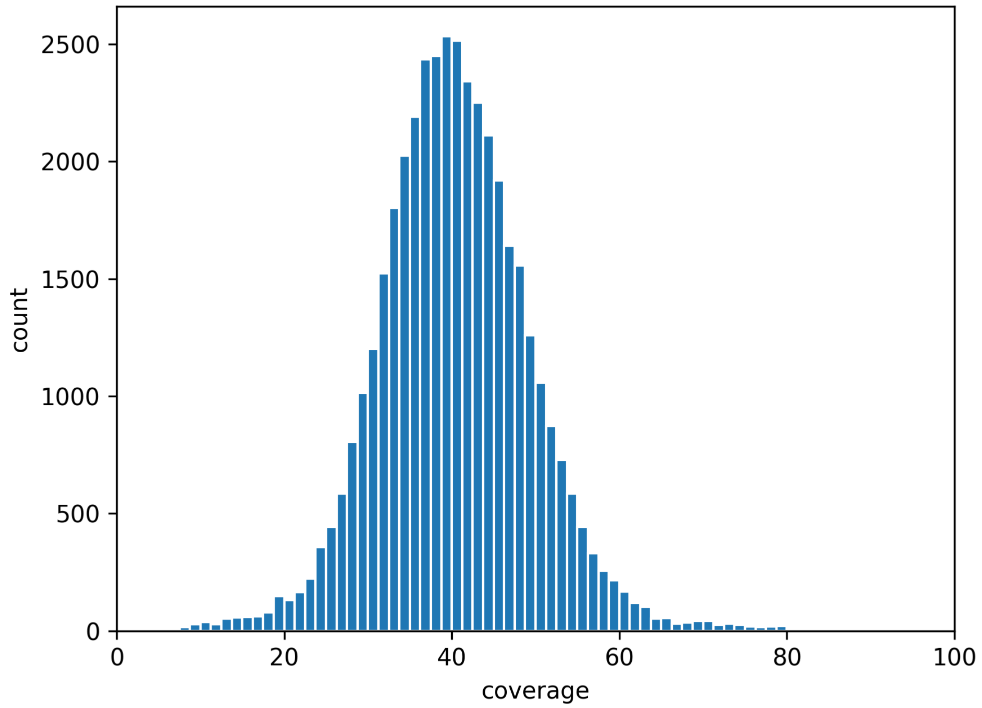


Figure 7: Coverage histogram of mean coverage over 1000 bp on chromosome 7 of haplophase A

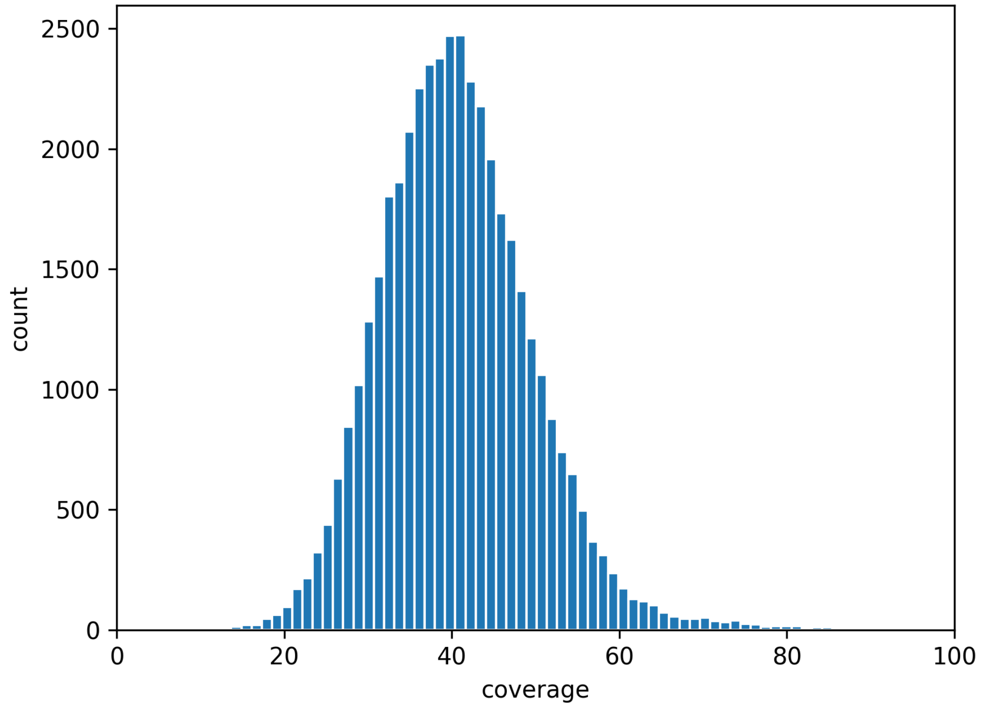


Figure 8: Coverage histogram of mean coverage over 1000 bp on chromosome 8 of haplophase A

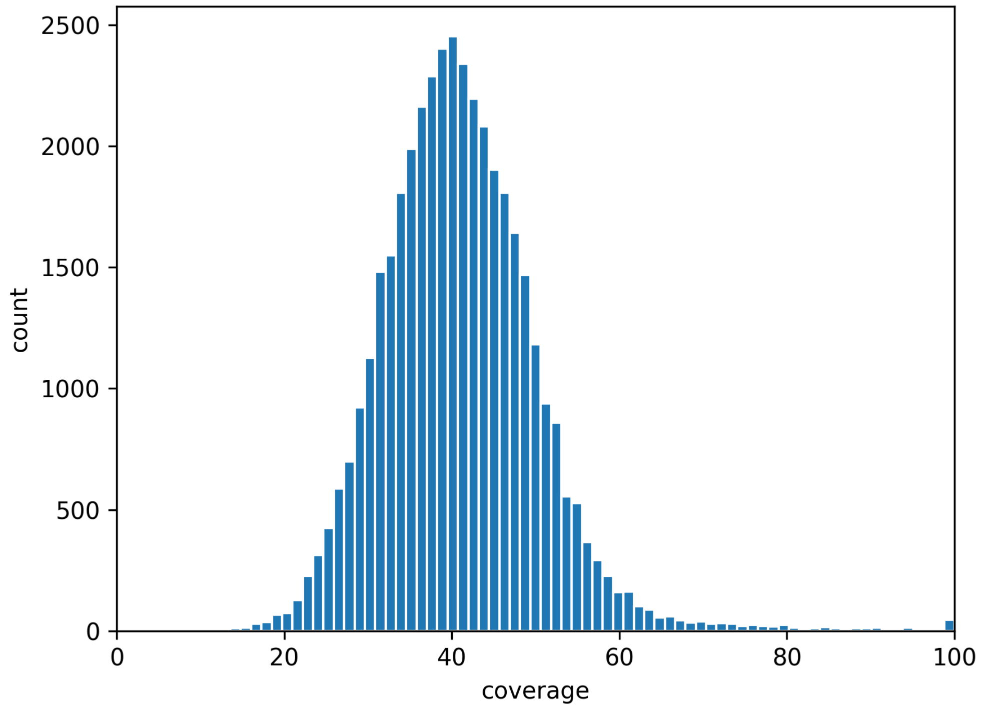


Figure 9: Coverage histogram of mean coverage over 1000 bp on chromosome 9 of haplophase A

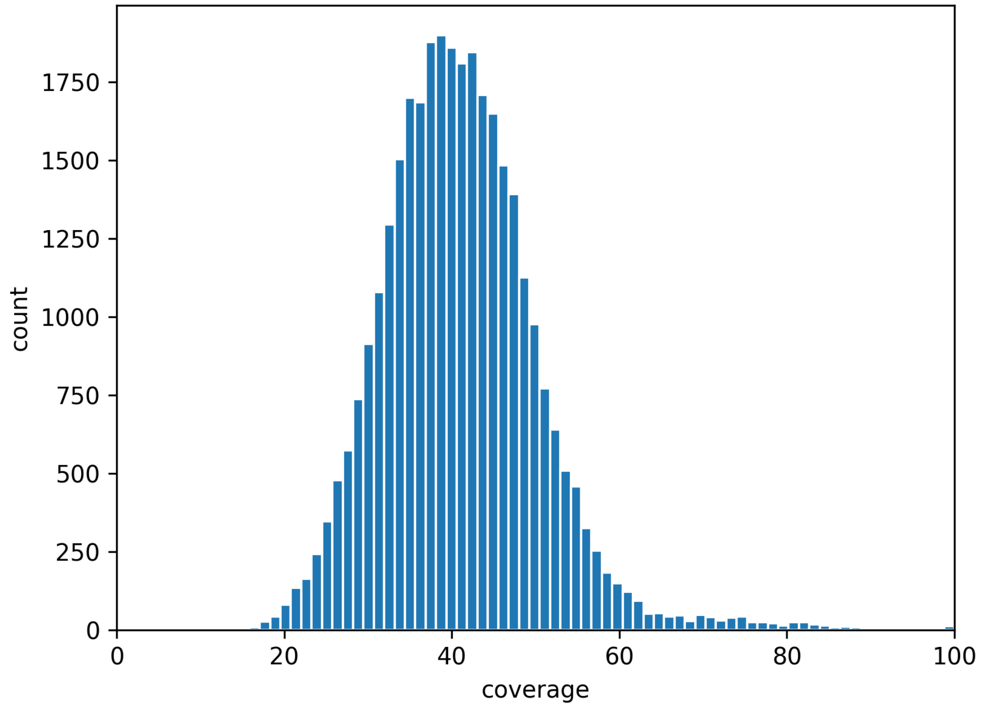


Figure 10: Coverage histogram of mean coverage over 1000 bp on chromosome 10 of haplophase A

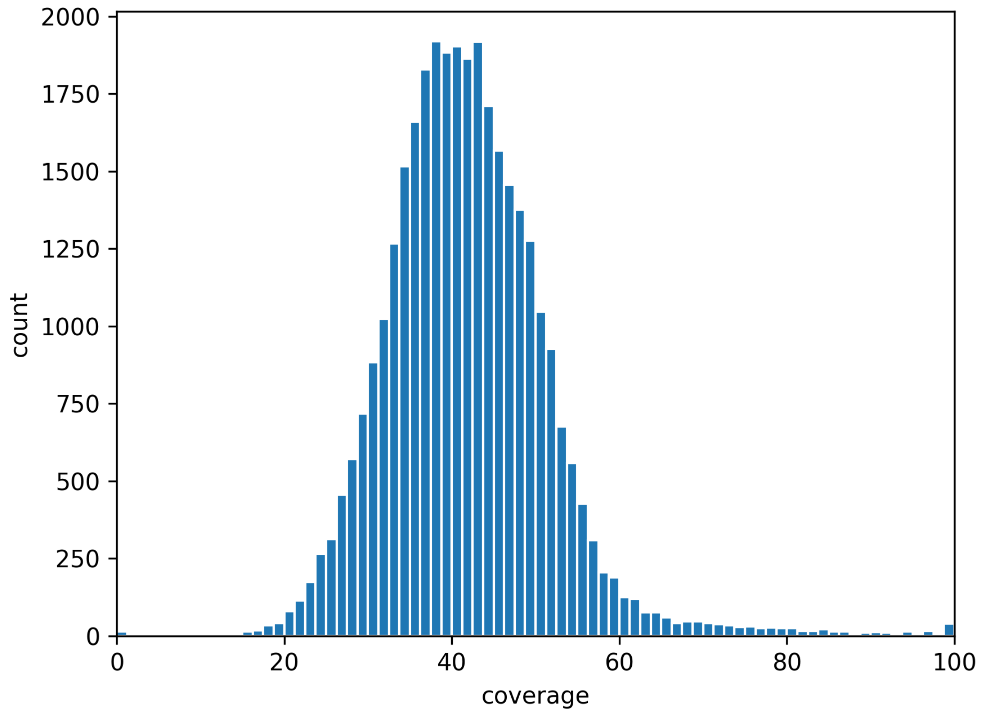


Figure 11: Coverage histogram of mean coverage over 1000 bp on chromosome 11 of haplophase A

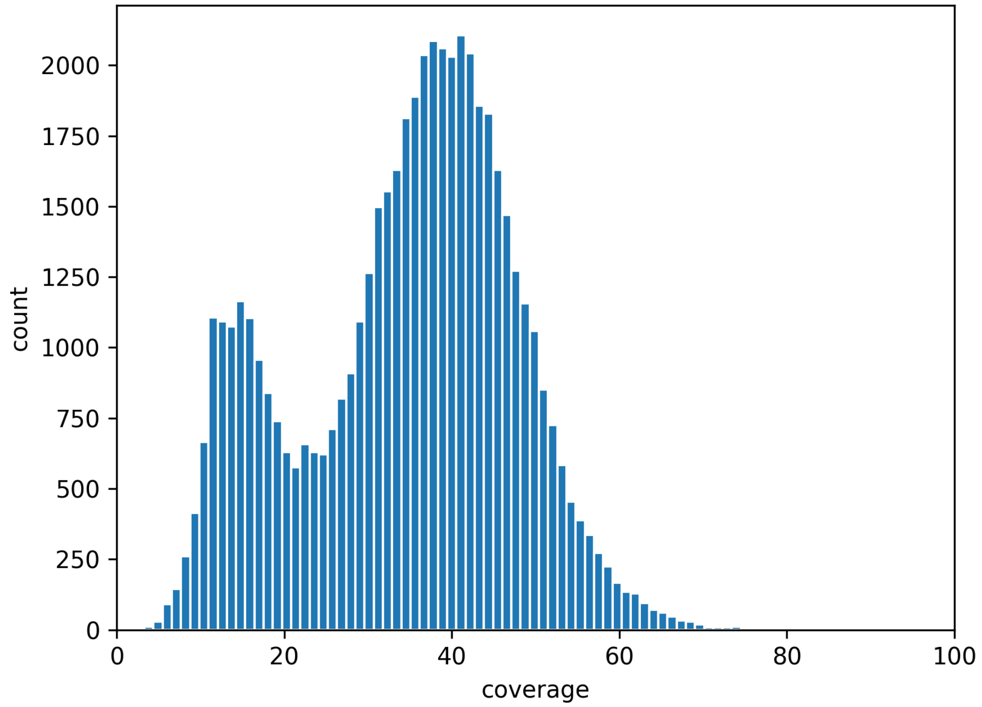


Figure 12: Coverage histogram of mean coverage over 1000 bp on chromosome 12 of haplophase A

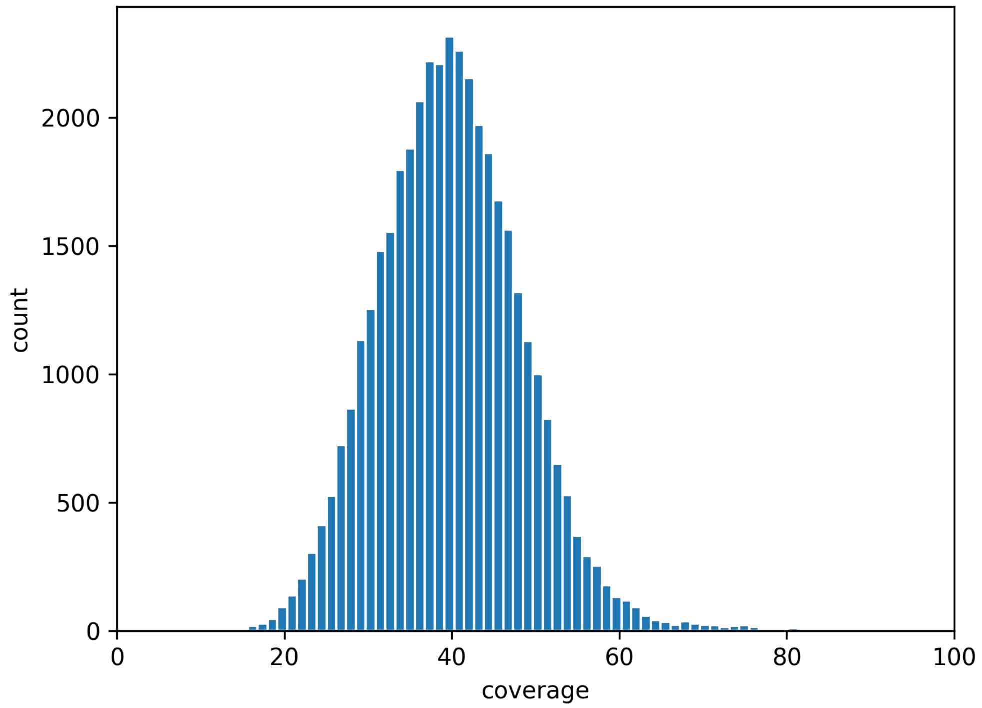


Figure 13: Coverage histogram of mean coverage over 1000 bp on chromosome 13 of haplophase A

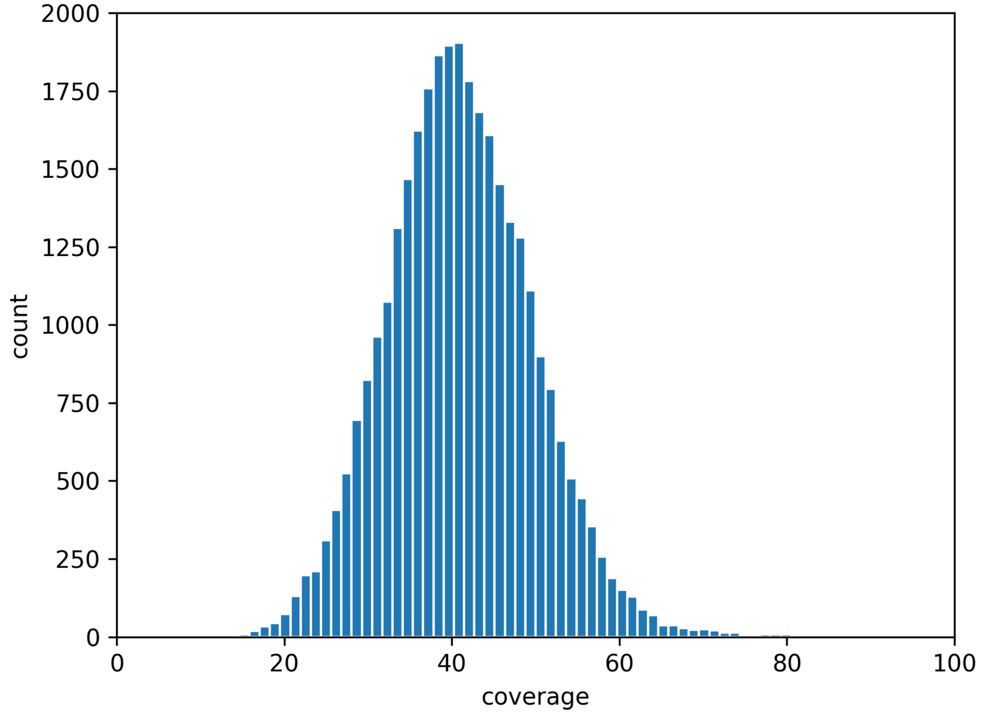


Figure 14: Coverage histogram of mean coverage over 1000 bp on chromosome 14 of haplophase A

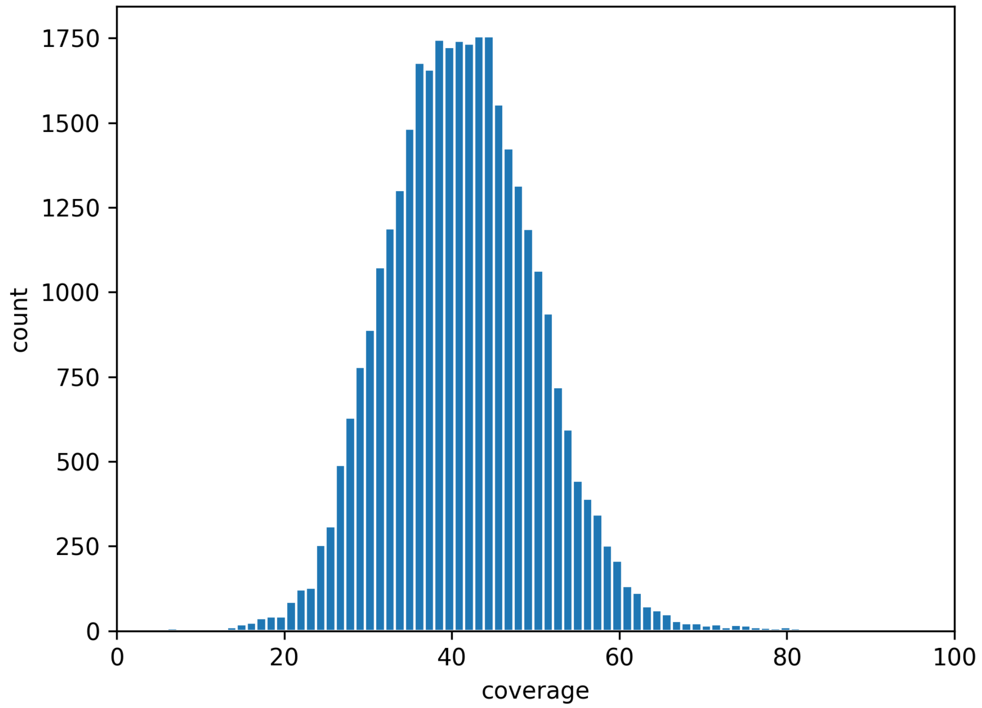


Figure 15: Coverage histogram of mean coverage over 1000 bp on chromosome 15 of haplophase A

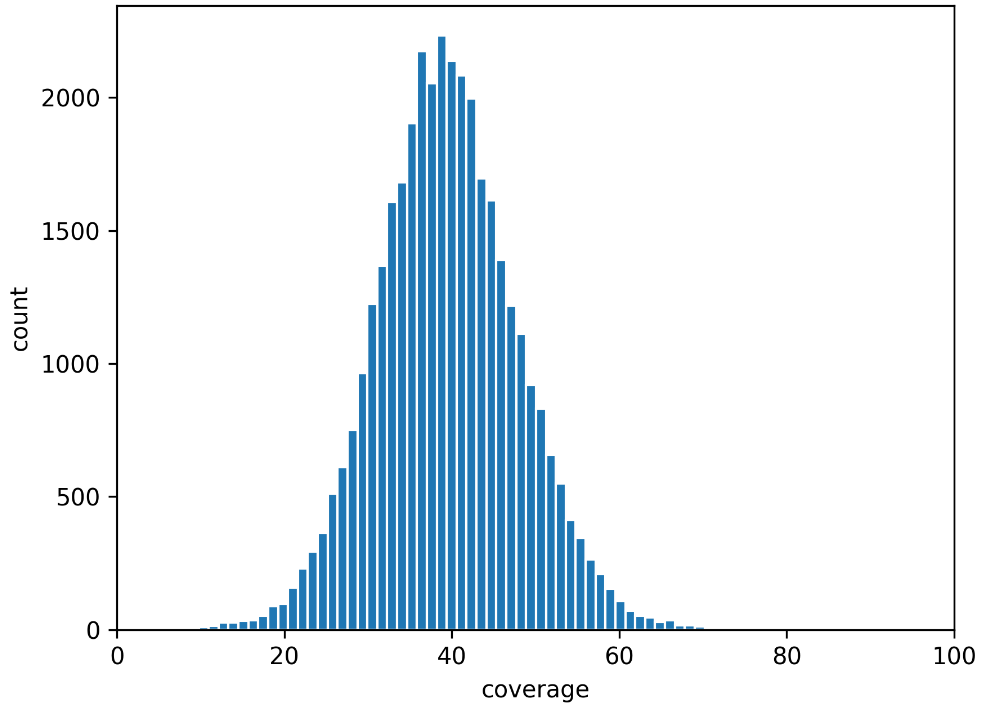


Figure 16: Coverage histogram of mean coverage over 1000 bp on chromosome 16 of haplophase A

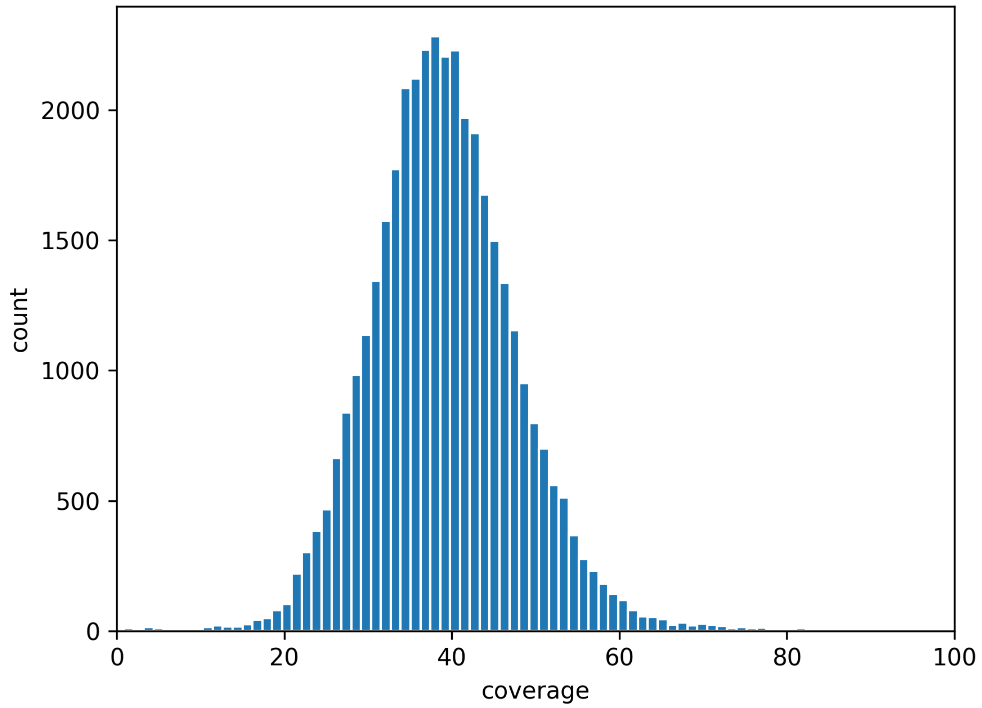


Figure 17: Coverage histogram of mean coverage over 1000 bp on chromosome 17 of haplophase A

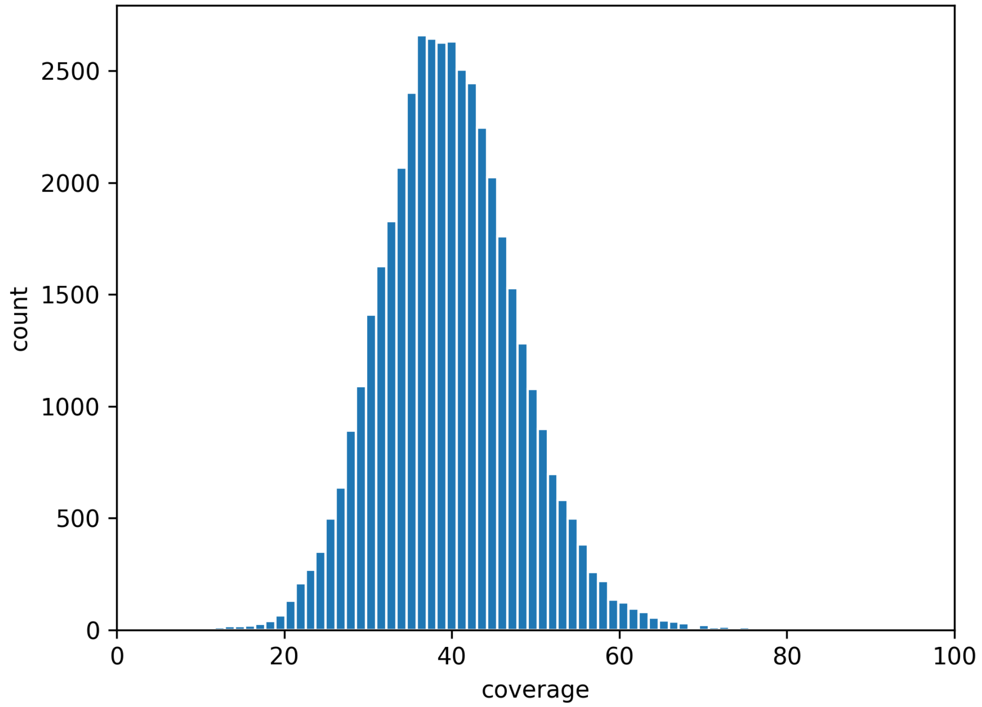


Figure 18: Coverage histogram of mean coverage over 1000 bp on chromosome 18 of haplophase A
