# Supplementary material for: The haplotype-resolved assembly of COL40 a cassava (Manihot esculenta) line with broad-spectrum resistance against viruses causing Cassava brown streak disease unveils a region of highly repeated elements on chromosome 12
Source: G3 (Bethesda). 2025 Apr 16;15(6):jkaf083. doi: 10.1093/g3journal/jkaf083 (PMC12134984; doi:10.1093/g3journal/jkaf083)
Supplement: jkaf083_Supplementary_Data [file jkaf083_supplementary_data.zip › Additional_File_4_G3-2024-405442.pdf]

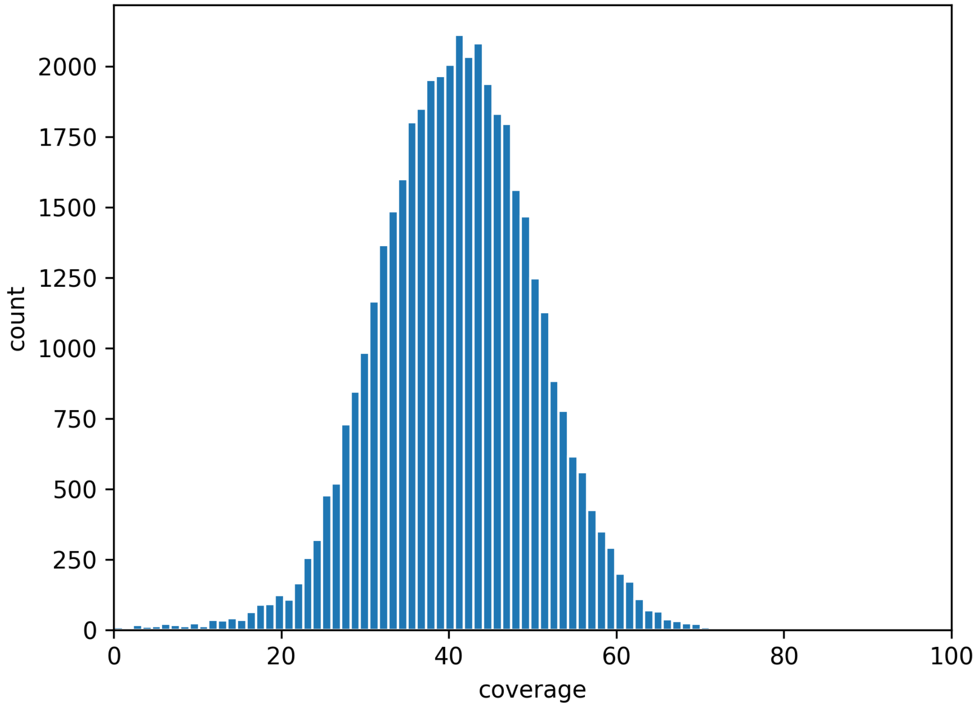

Figure 1: Coverage histogram of mean coverage over 1000 bp on chromosome 1 of haplophase B

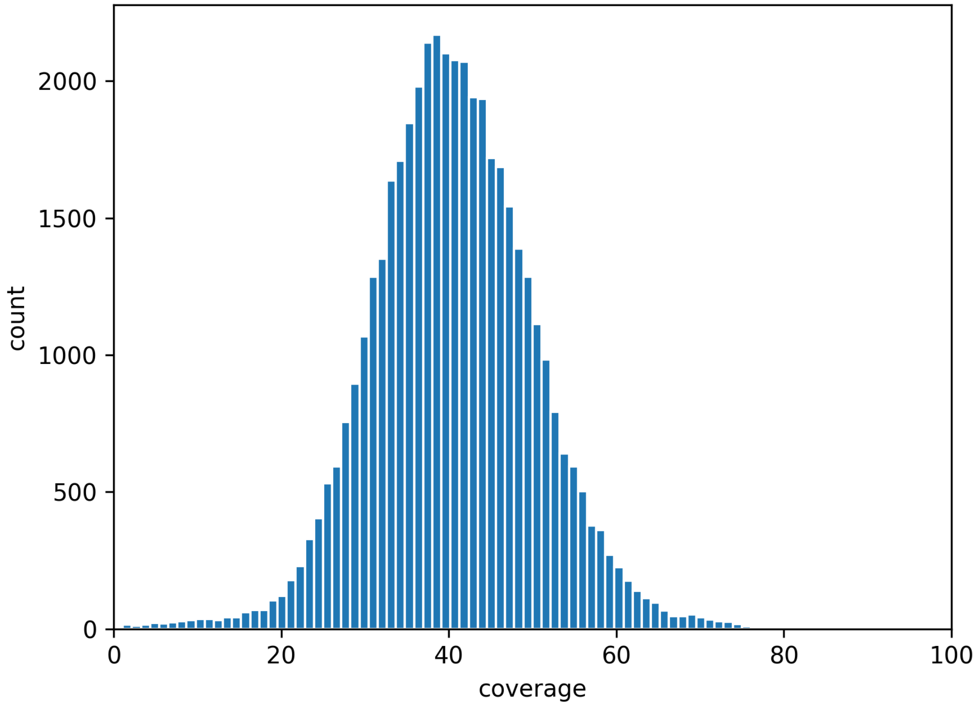

Figure 2: Coverage histogram of mean coverage over 1000 bp on chromosome 2 of haplophase B

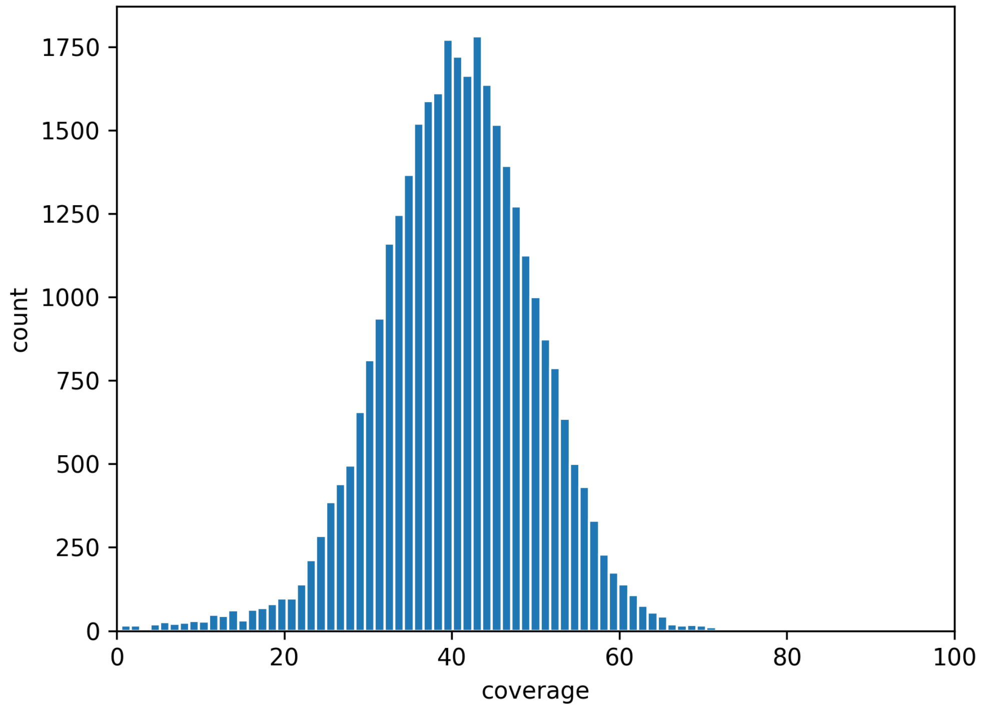

Figure 3: Coverage histogram of mean coverage over 1000 bp on chromosome 3 of haplophase B

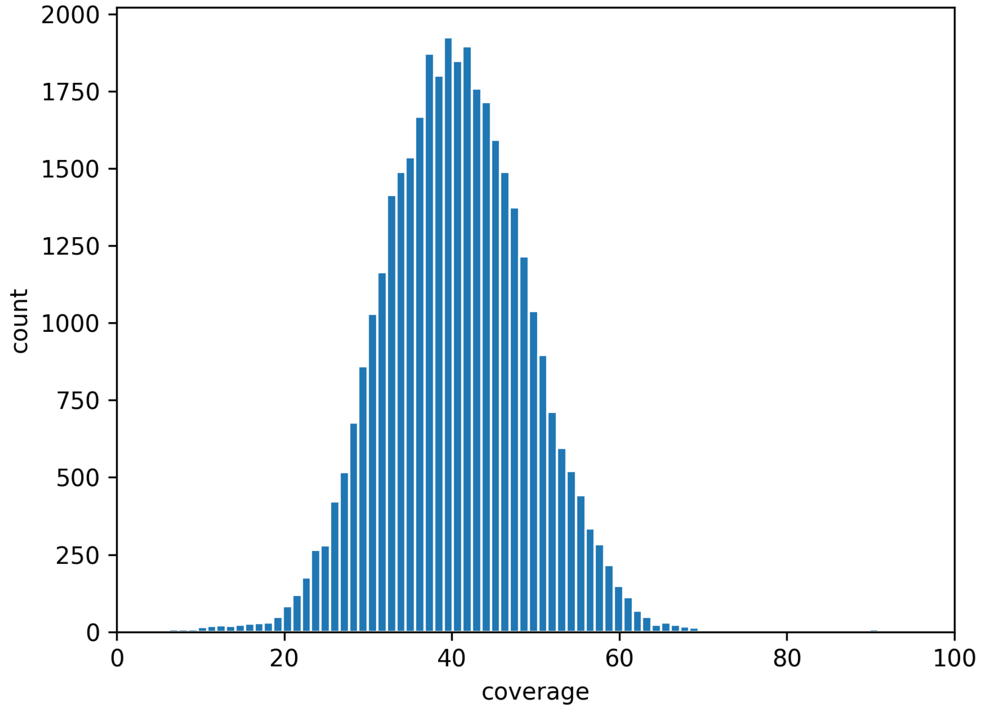

Figure 4: Coverage histogram of mean coverage over 1000 bp on chromosome 4 of haplophase B

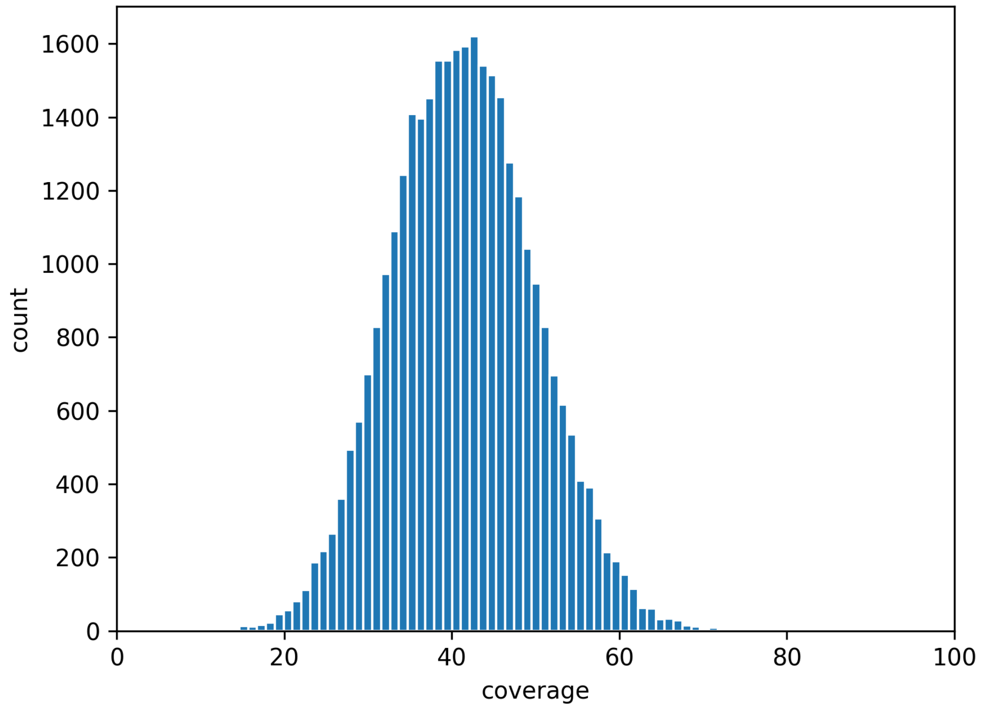

Figure 5: Coverage histogram of mean coverage over 1000 bp on chromosome 5 of haplophase B

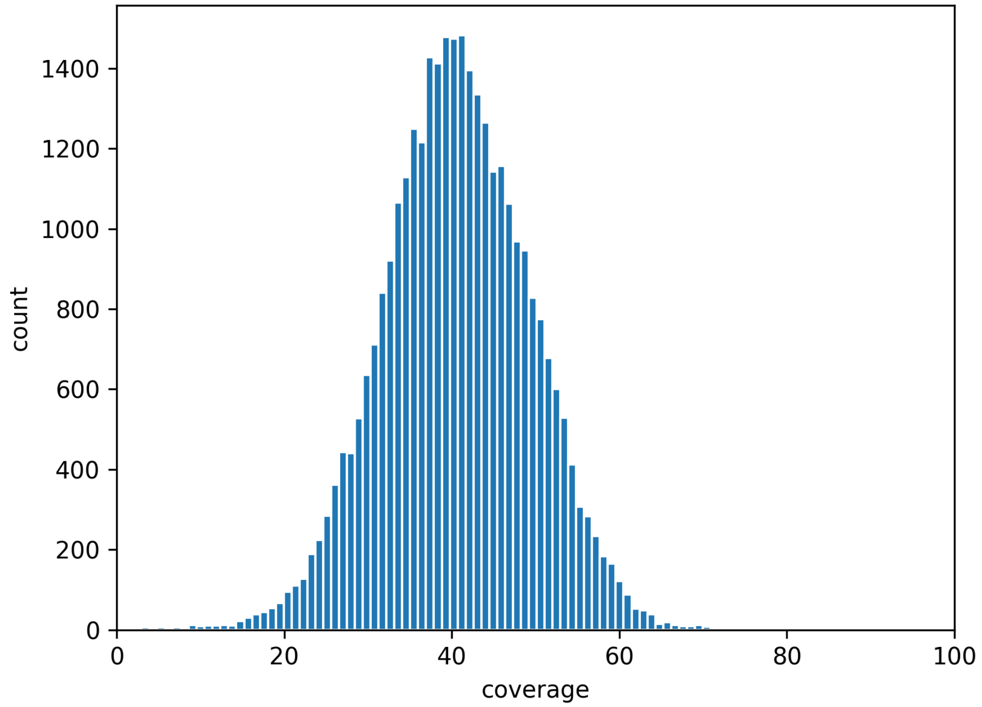

Figure 6: Coverage histogram of mean coverage over 1000 bp on chromosome 6 of haplophase B

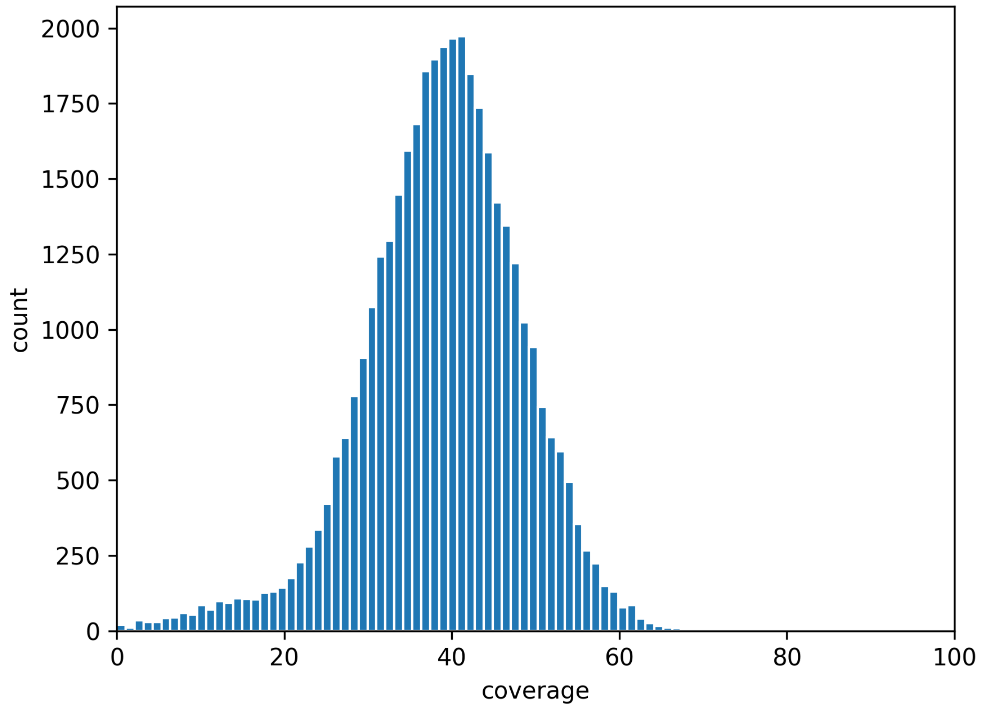

Figure 7: Coverage histogram of mean coverage over 1000 bp on chromosome 7 of haplophase B

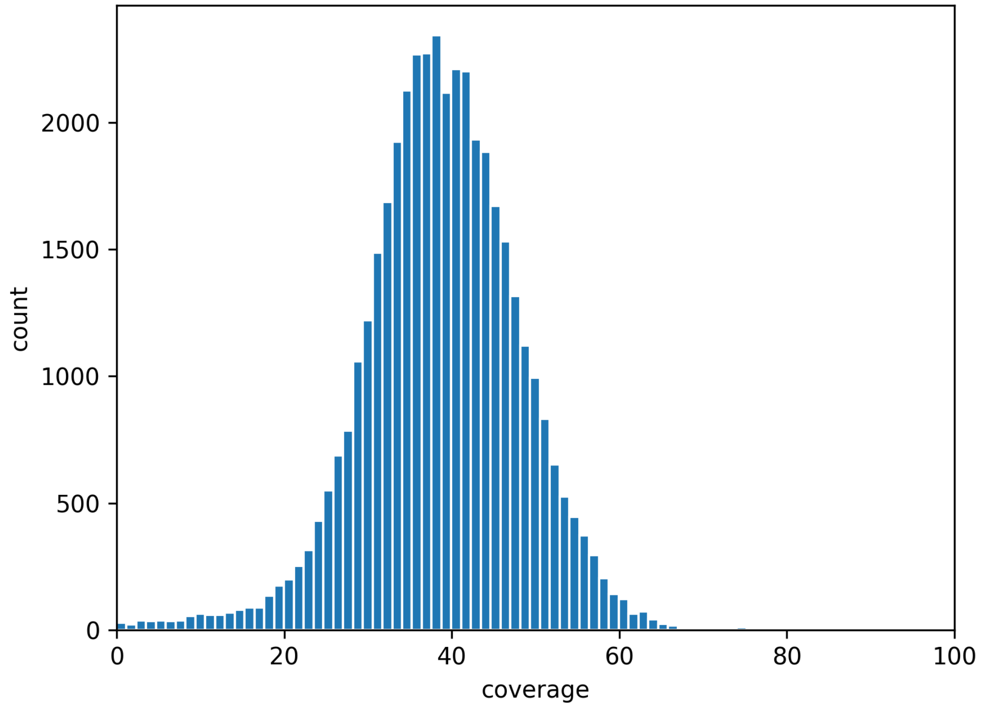

Figure 8: Coverage histogram of mean coverage over 1000 bp on chromosome 8 of haplophase B

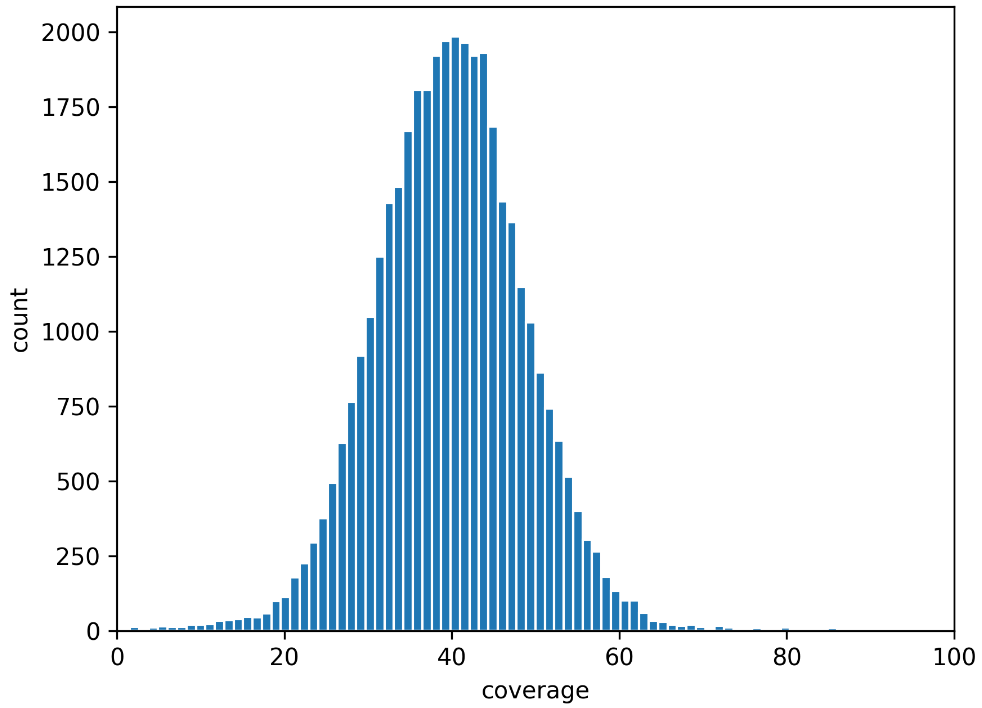

Figure 9: Coverage histogram of mean coverage over 1000 bp on chromosome 9 of haplophase B

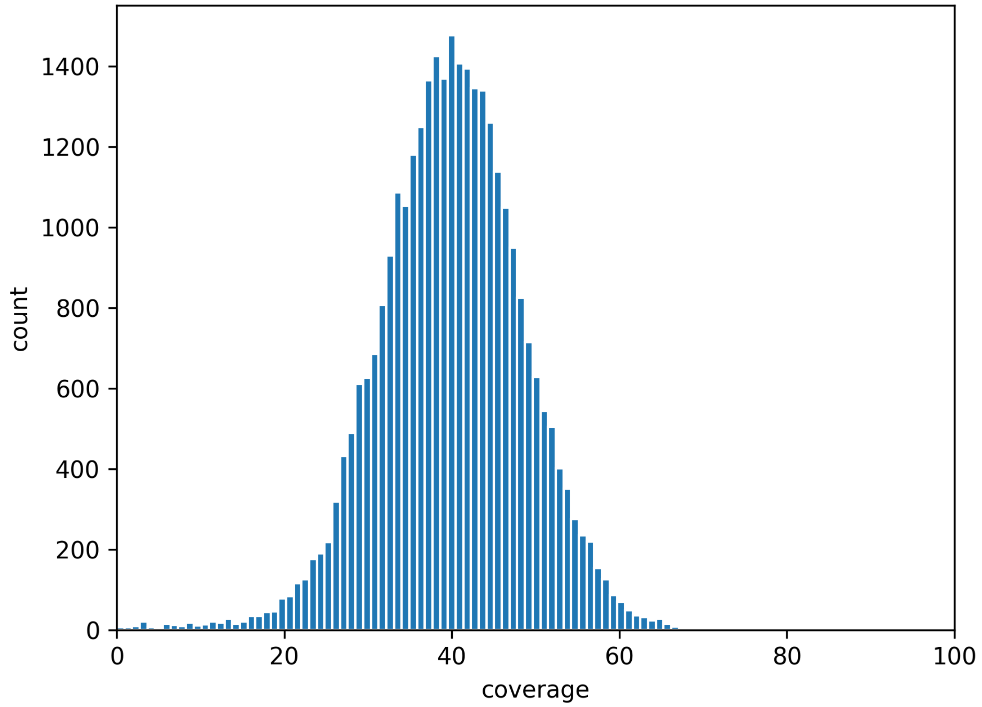

Figure 10: Coverage histogram of mean coverage over 1000 bp on chromosome 10 of haplophase B

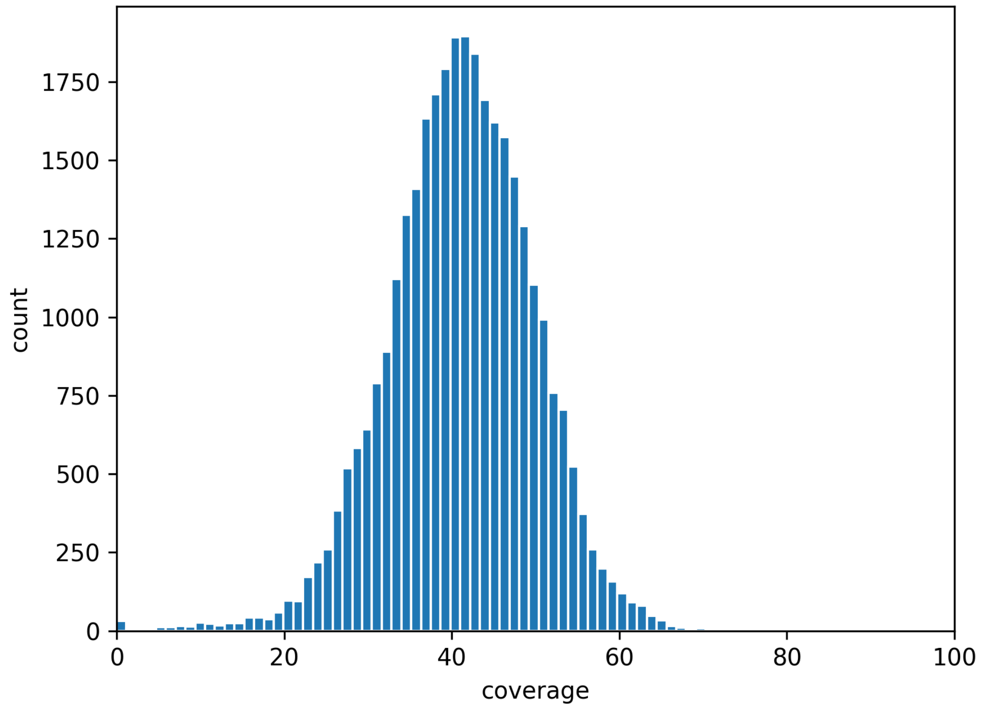

Figure 11: Coverage histogram of mean coverage over 1000 bp on chromosome 11 of haplophase B

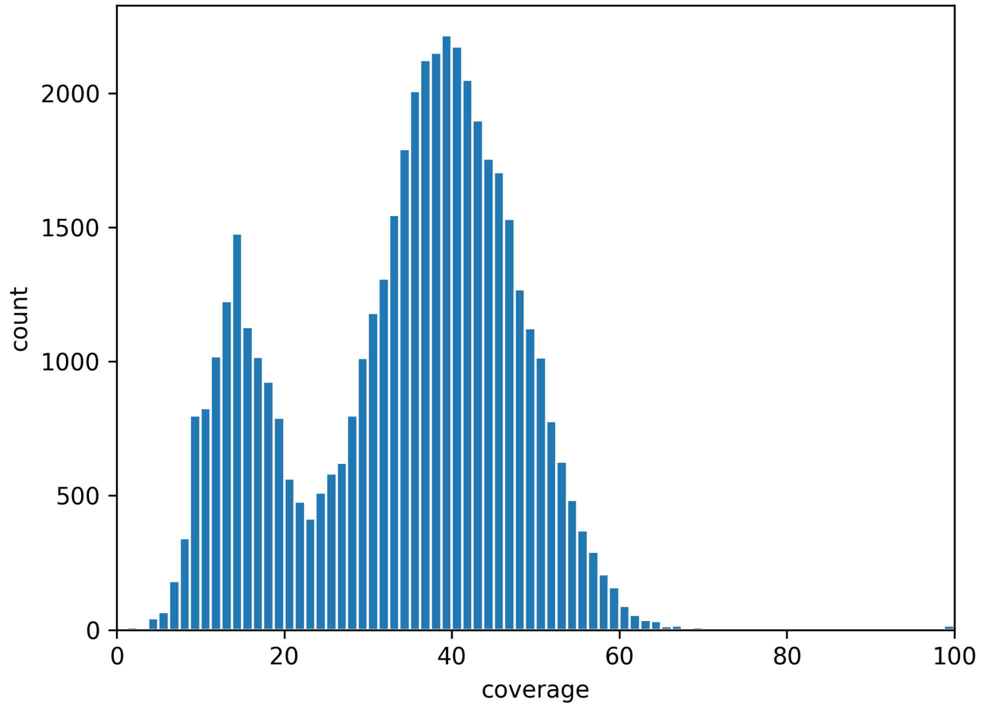

Figure 12: Coverage histogram of mean coverage over 1000 bp on chromosome 12 of haplophase B

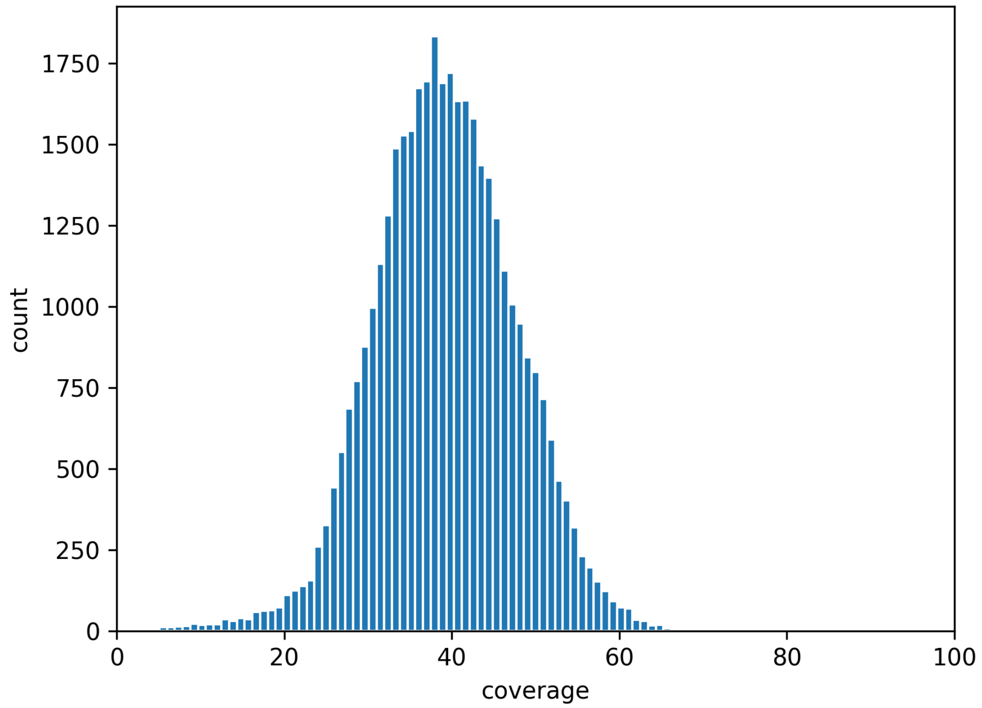

Figure 13: Coverage histogram of mean coverage over 1000 bp on chromosome 13 of haplophase B

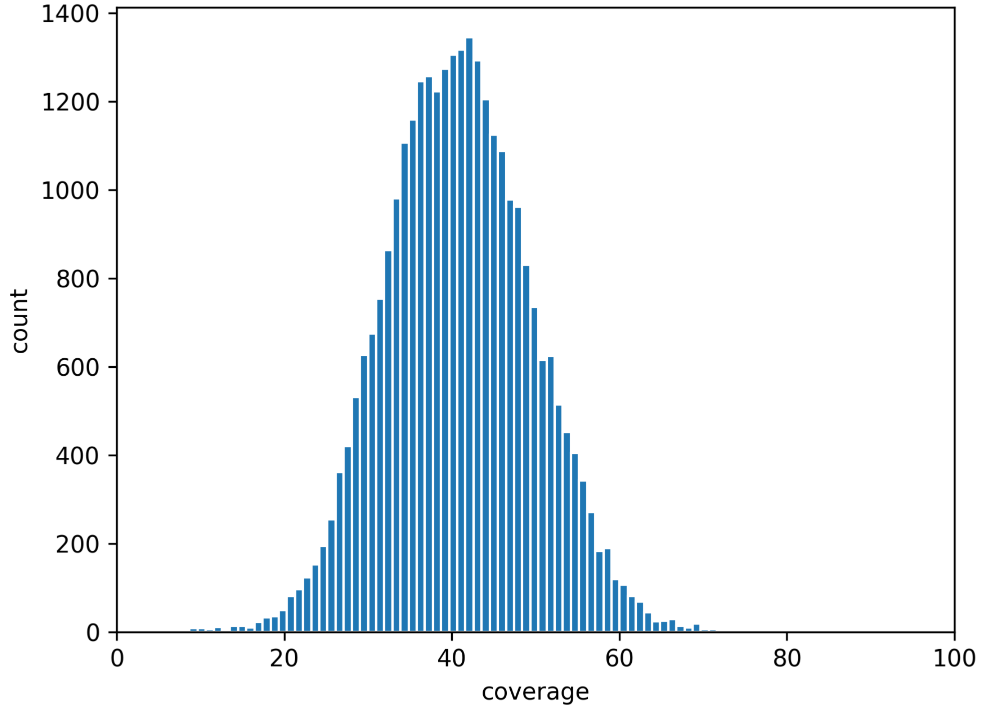

Figure 14: Coverage histogram of mean coverage over 1000 bp on chromosome 14 of haplophase B

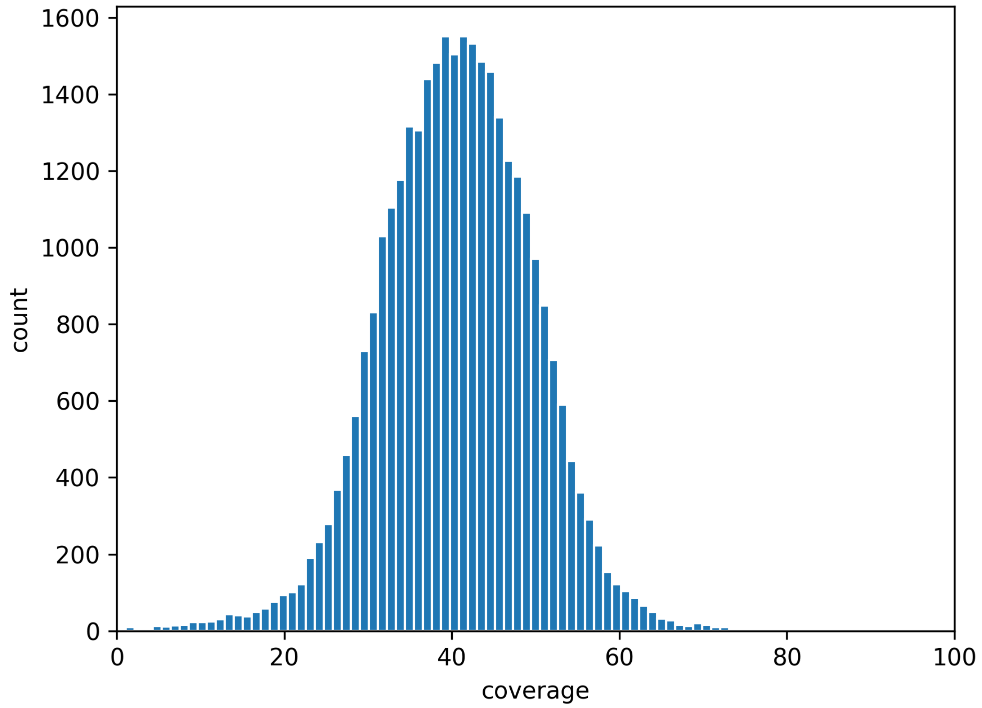

Figure 15: Coverage histogram of mean coverage over 1000 bp on chromosome 15 of haplophase B

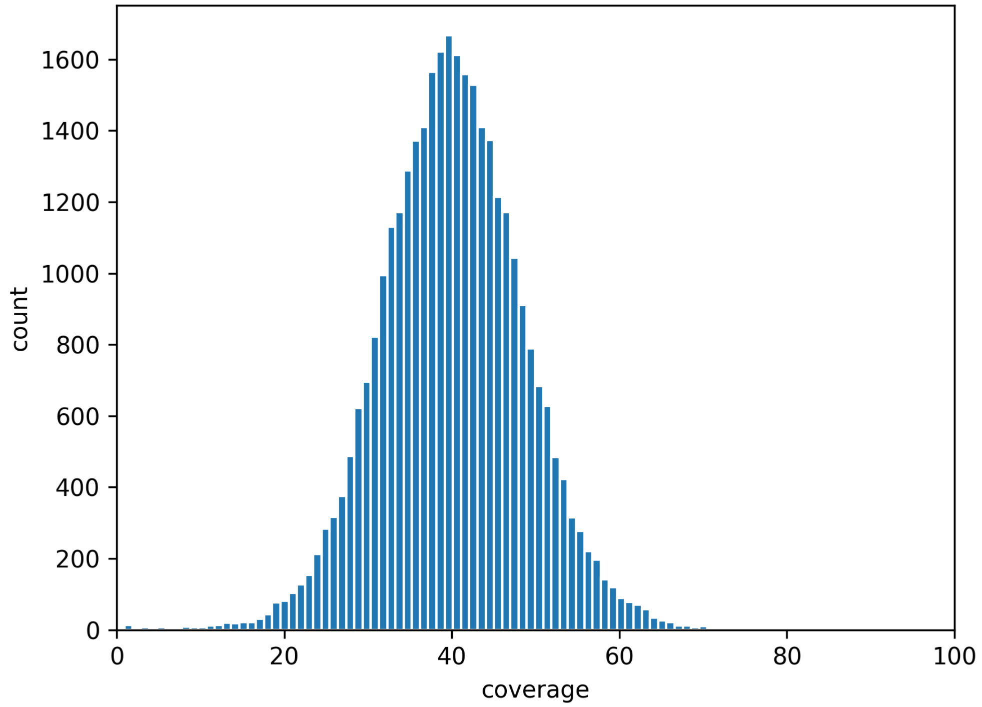

Figure 16: Coverage histogram of mean coverage over 1000 bp on chromosome 16 of haplophase B

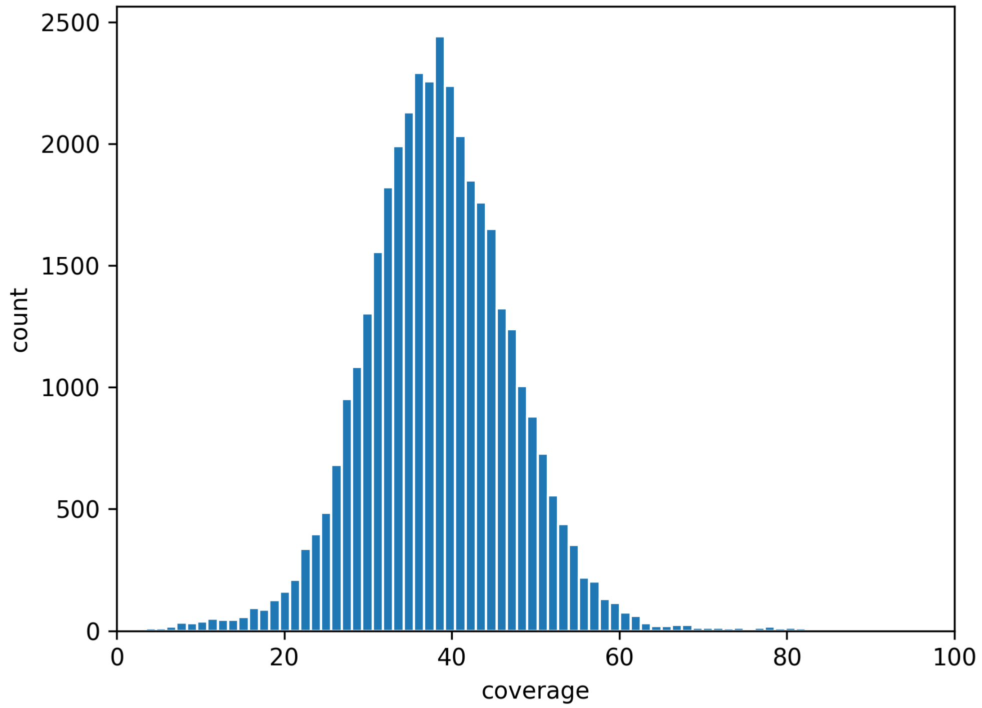

Figure 17: Coverage histogram of mean coverage over 1000 bp on chromosome 17 of haplophase B

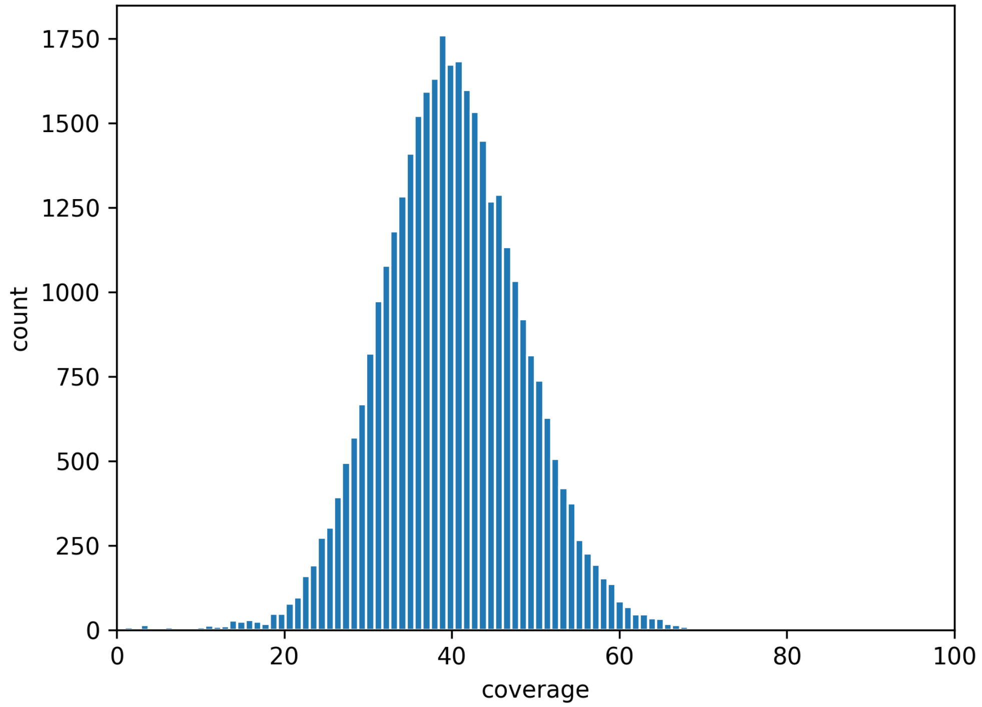

Figure 18: Coverage histogram of mean coverage over 1000 bp on chromosome 18 of haplophase B
