# Supplementary material for: The haplotype-resolved assembly of COL40 a cassava (Manihot esculenta) line with broad-spectrum resistance against viruses causing Cassava brown streak disease unveils a region of highly repeated elements on chromosome 12
Source: G3 (Bethesda). 2025 Apr 16;15(6):jkaf083. doi: 10.1093/g3journal/jkaf083 (PMC12134984; doi:10.1093/g3journal/jkaf083)
Supplement: jkaf083_Supplementary_Data [file jkaf083_supplementary_data.zip › Supplemental_File_Legends_G3-2024-405442.docx]

**Supplemental File Legends**

- Additional File 1: Scaffolding orientation for each chromosome of haplophase A created with ALLMAPS
- Additional File 2: Scaffolding orientation for each chromosome of haplophase B created with ALLMAPS
- Additional File 3: Coverage histograms for each chromosome of haplophase A
- Additional File 4: Coverage histograms for each chromosome of haplophase B
- Additional File 5: RNA-Seq libraries from COL40 and other cultivars used as hints in the functional annotation with BRAKER3
