# Supplementary figures and images for: The haplotype-resolved assembly of COL40 a cassava (Manihot esculenta) line with broad-spectrum resistance against viruses causing Cassava brown streak disease unveils a region of highly repeated elements on chromosome 12
Source: G3 (Bethesda). 2025 Apr 16;15(6):jkaf083. doi: 10.1093/g3journal/jkaf083 (PMC12134984; doi:10.1093/g3journal/jkaf083)

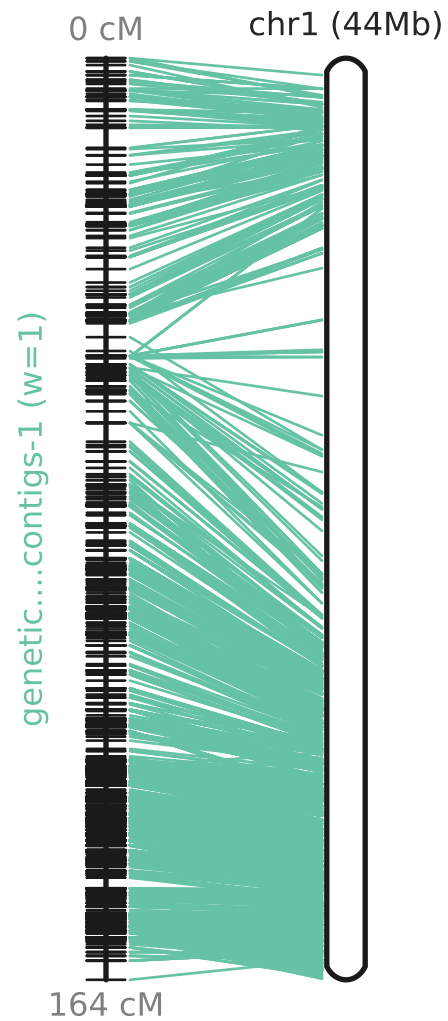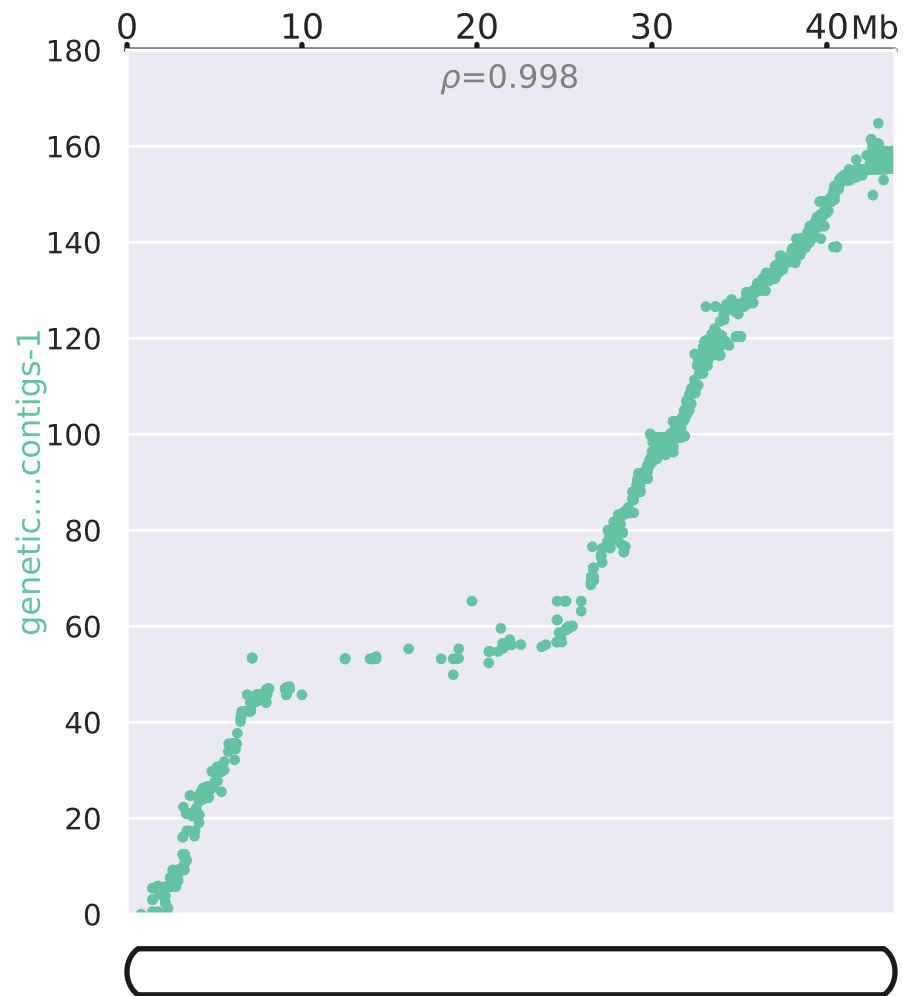

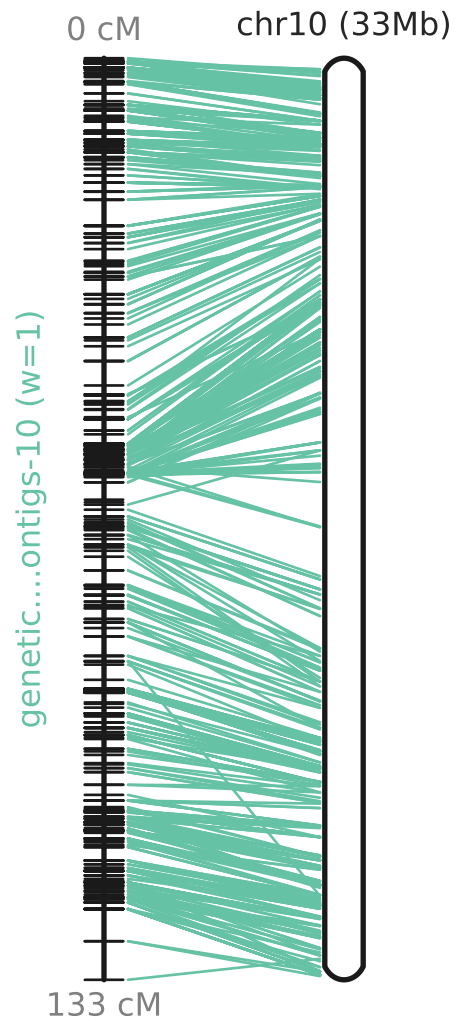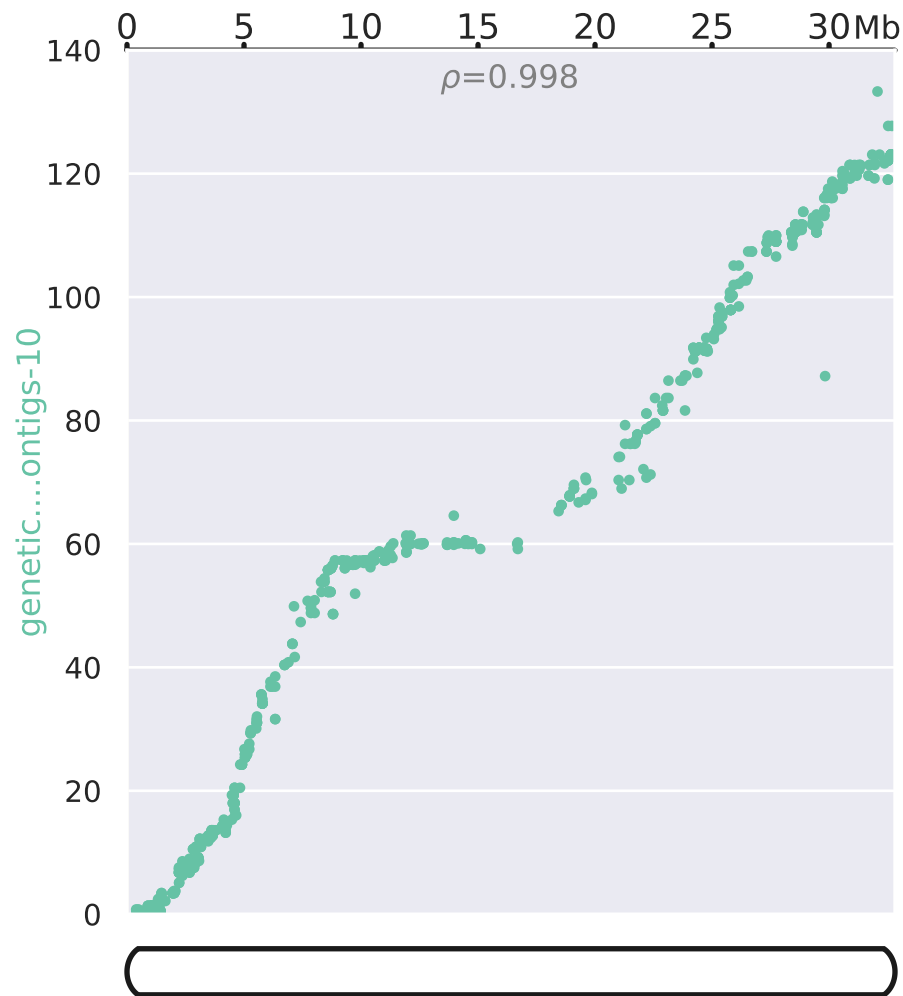

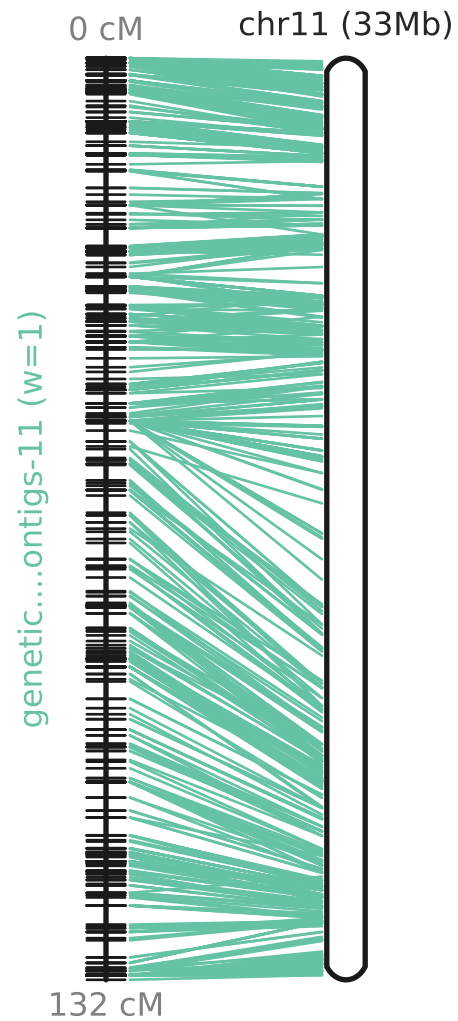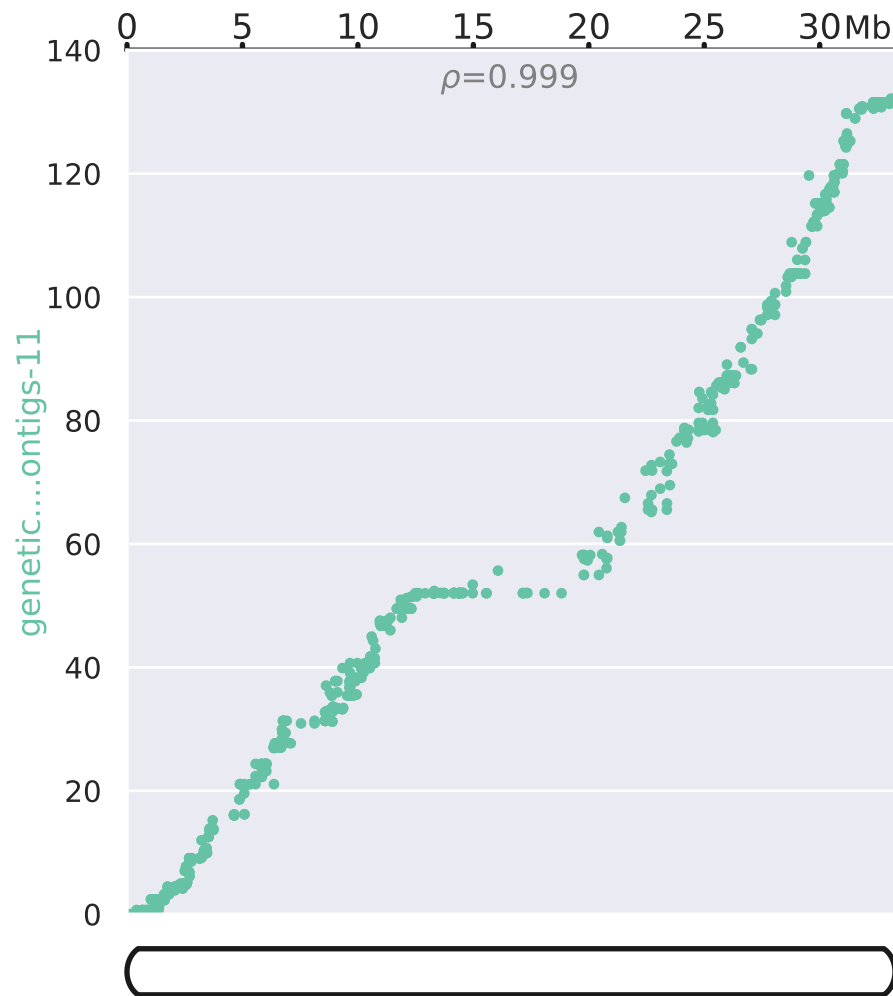

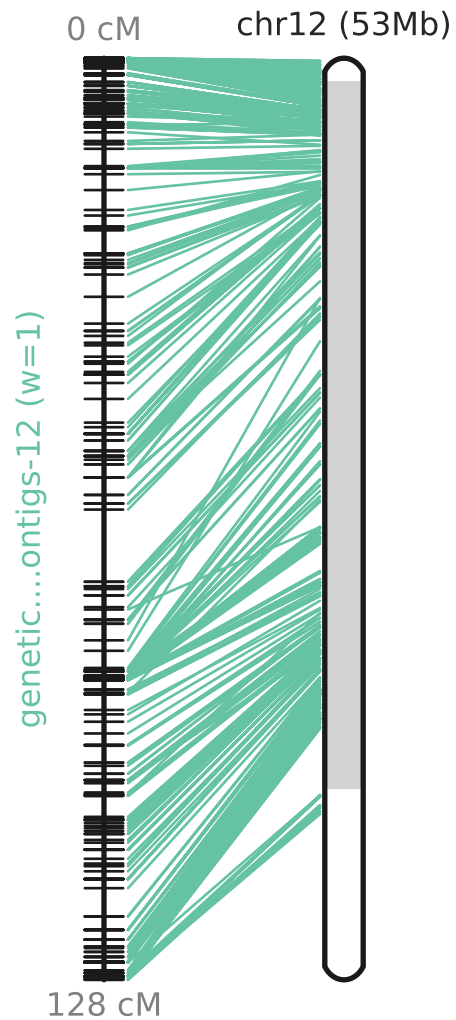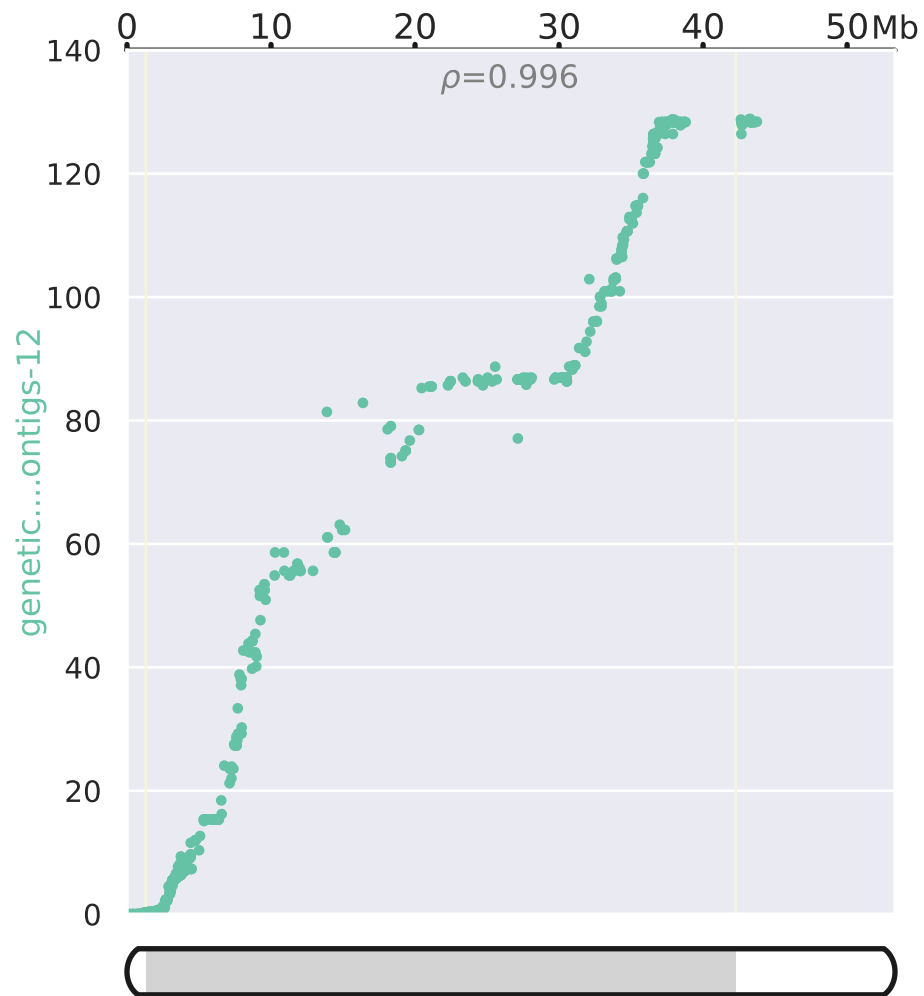

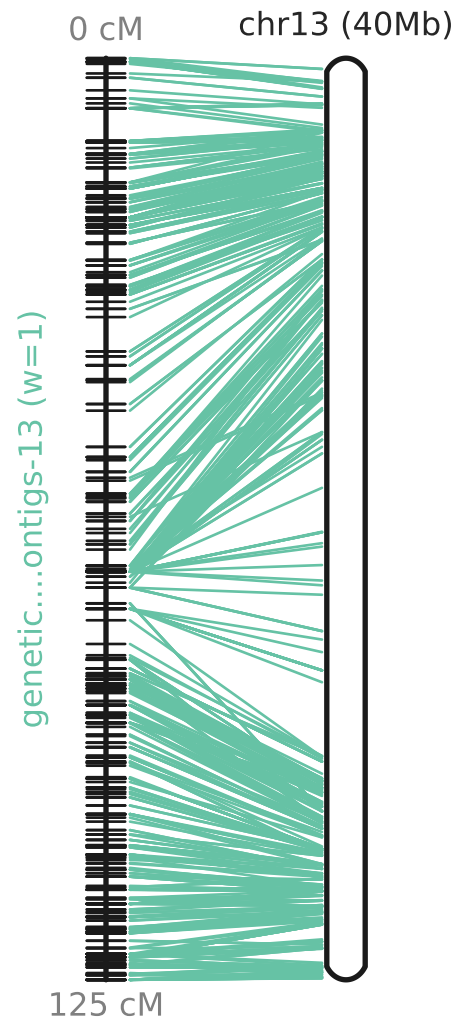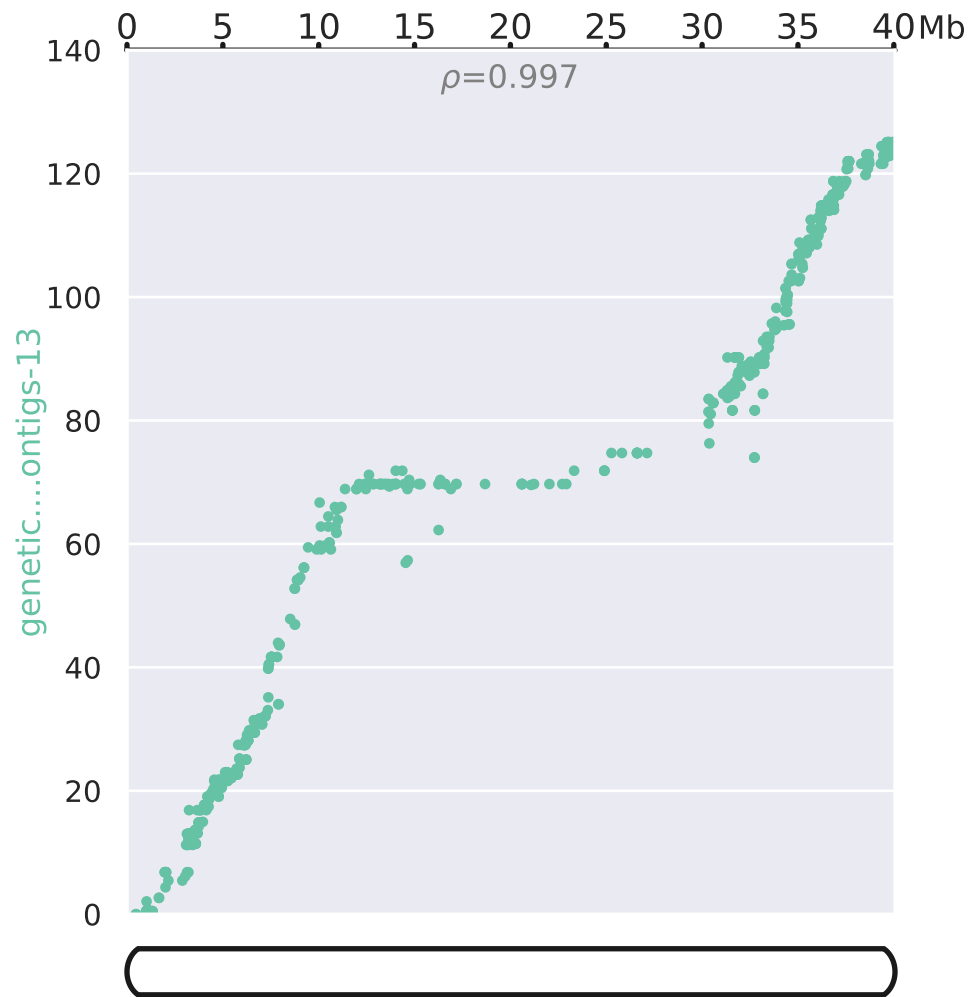

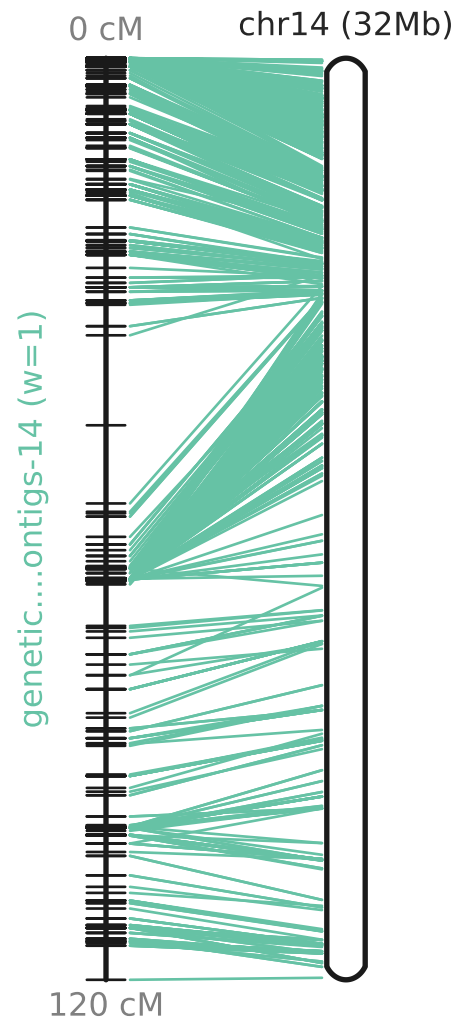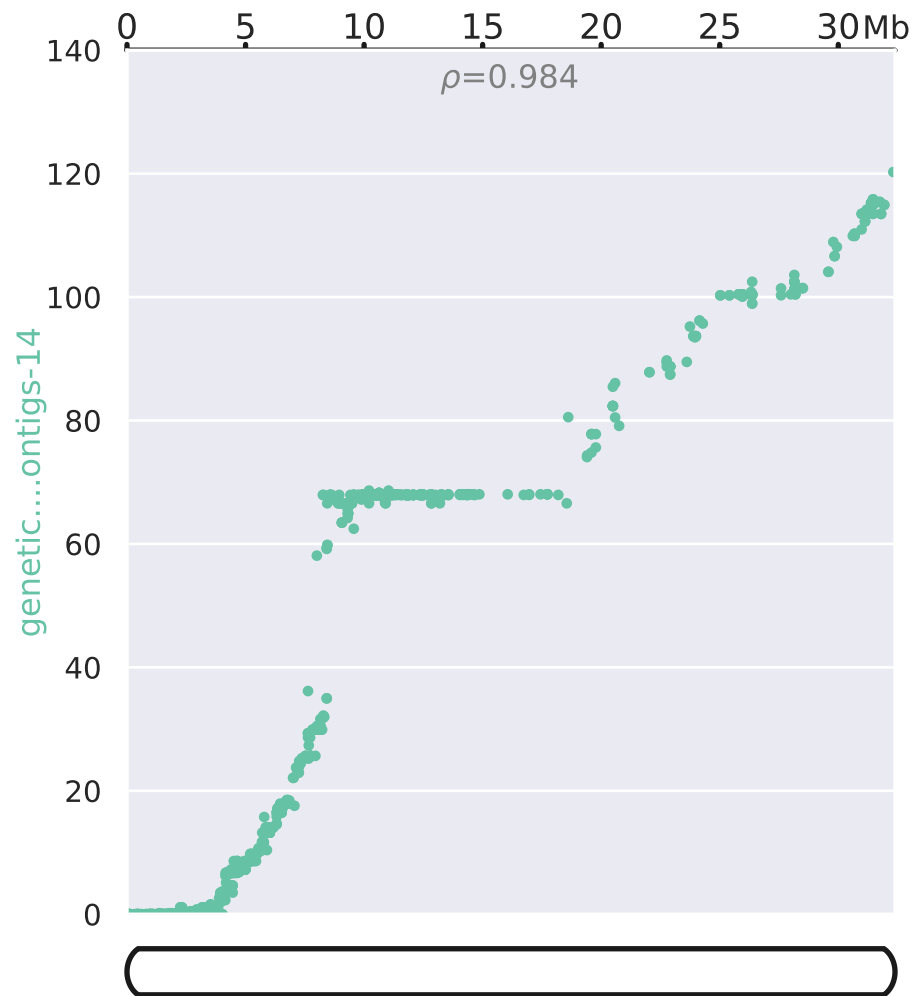

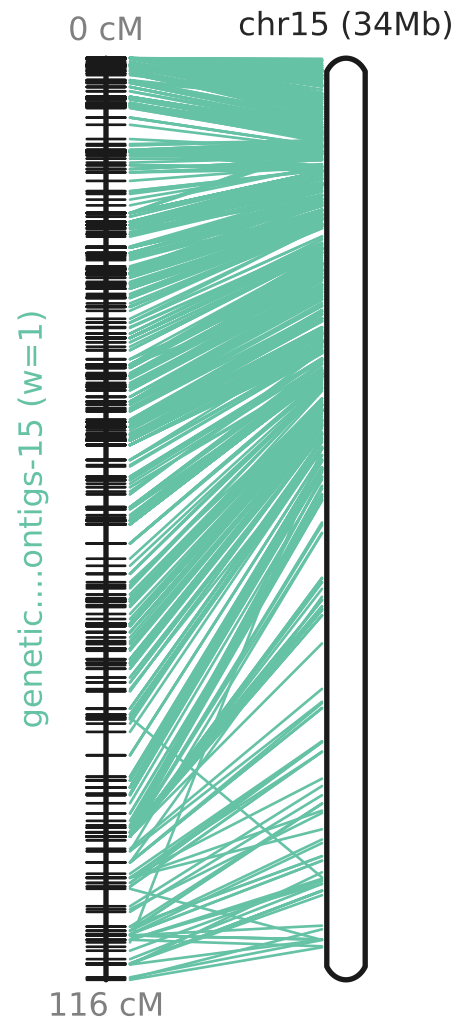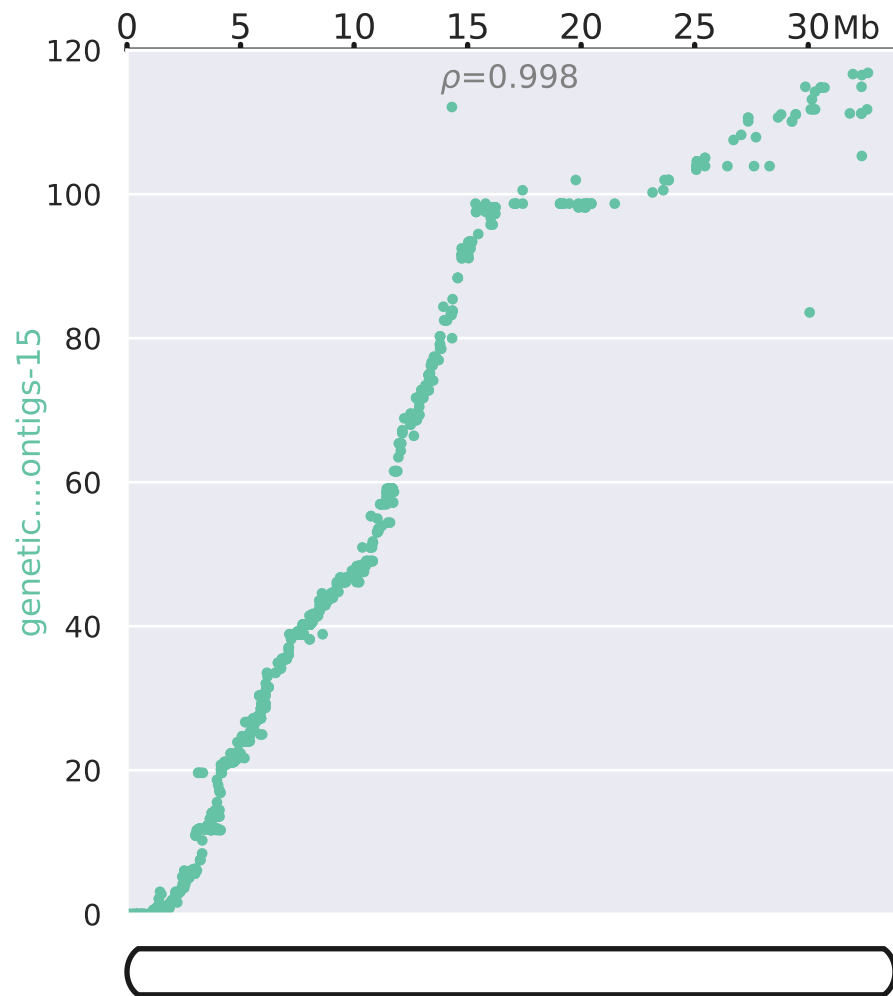

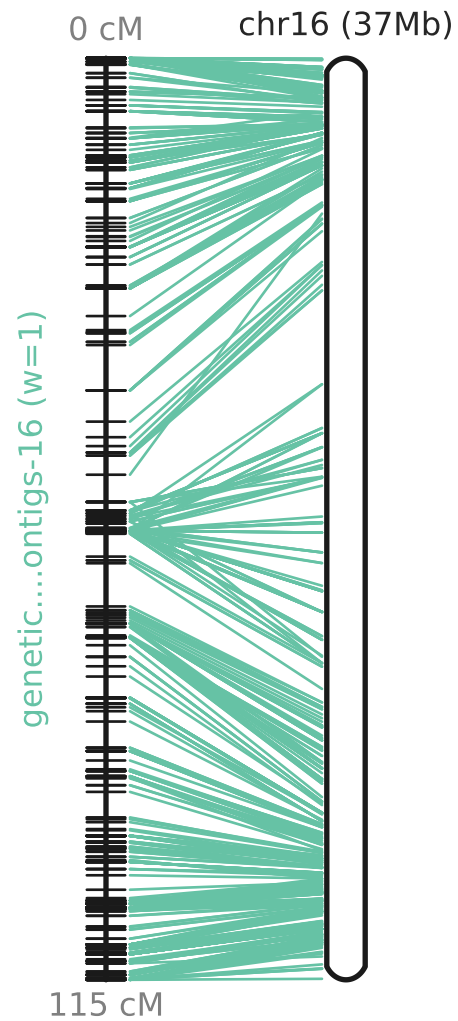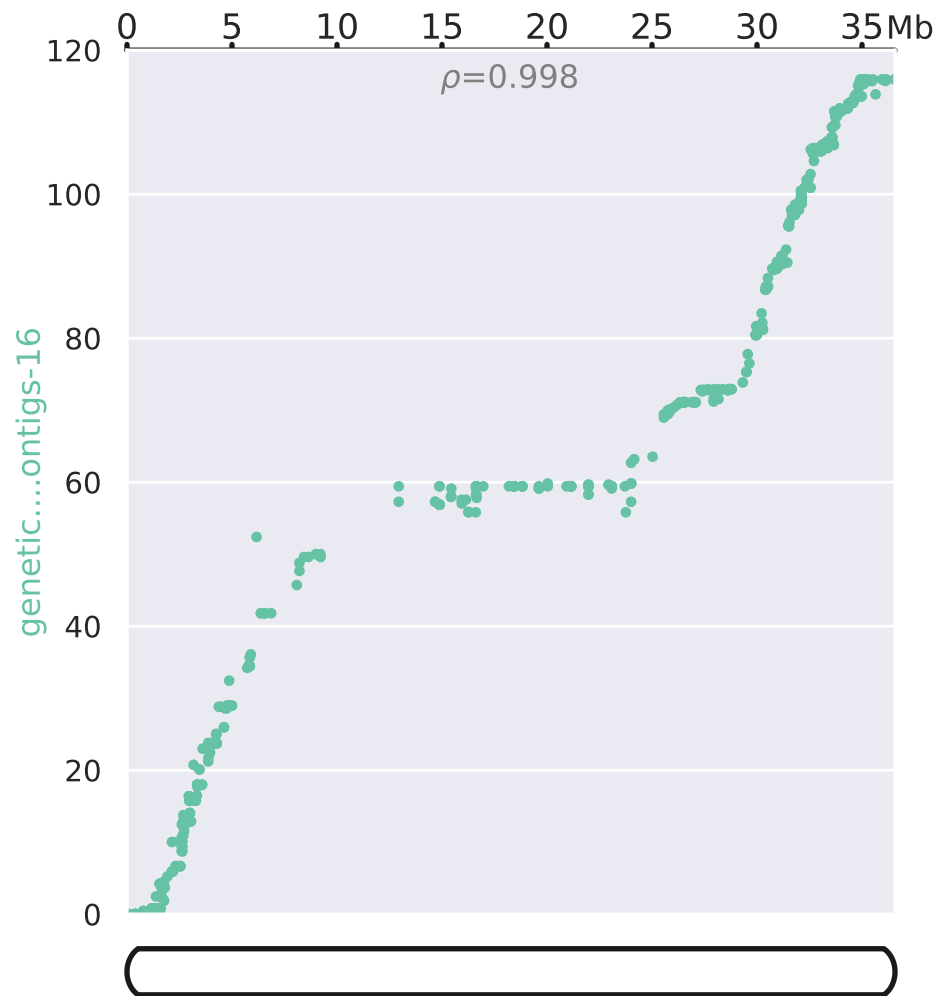

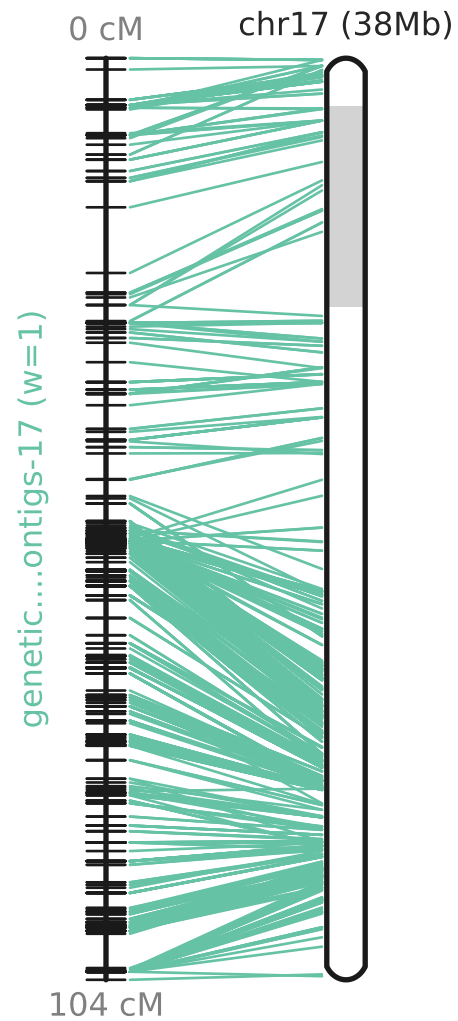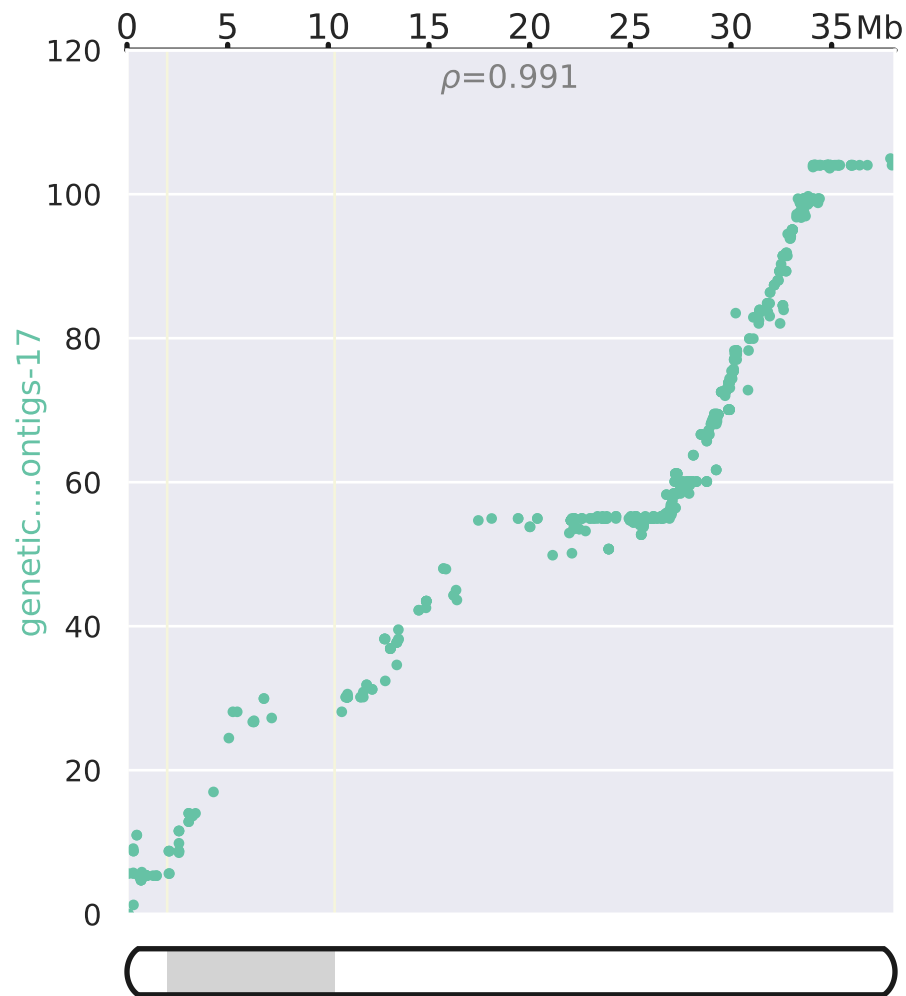

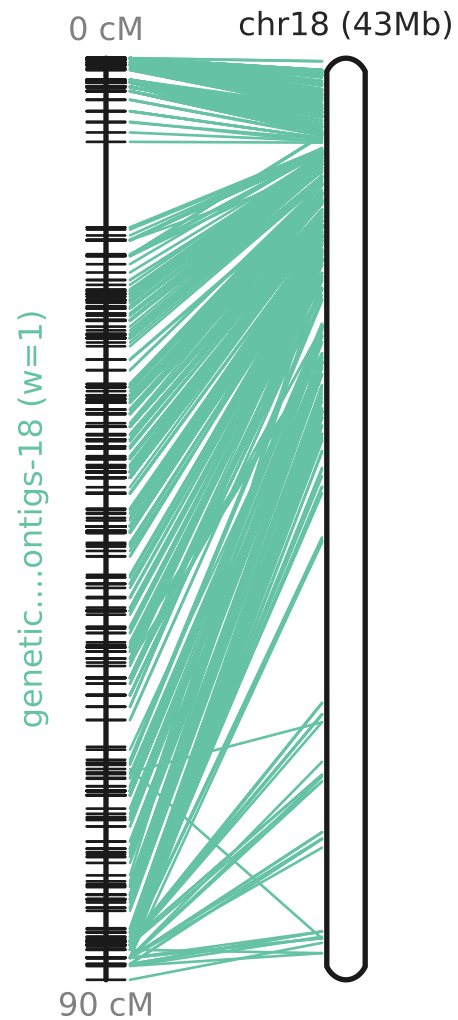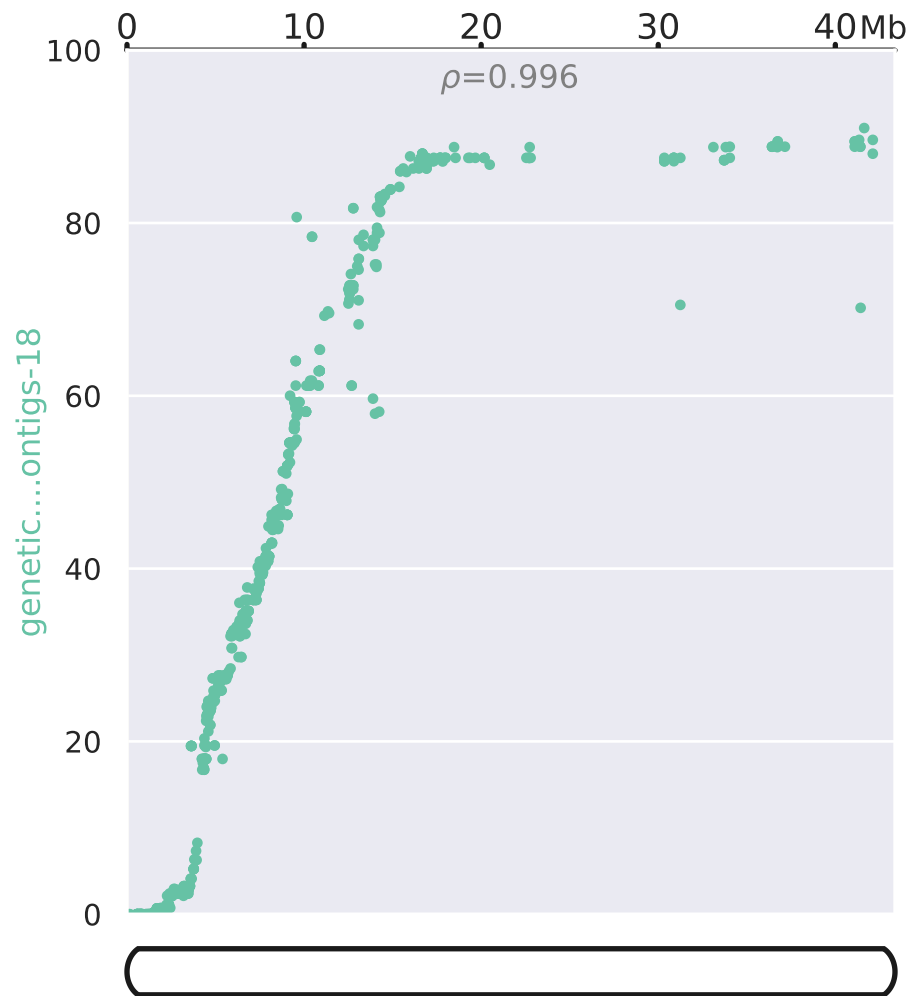

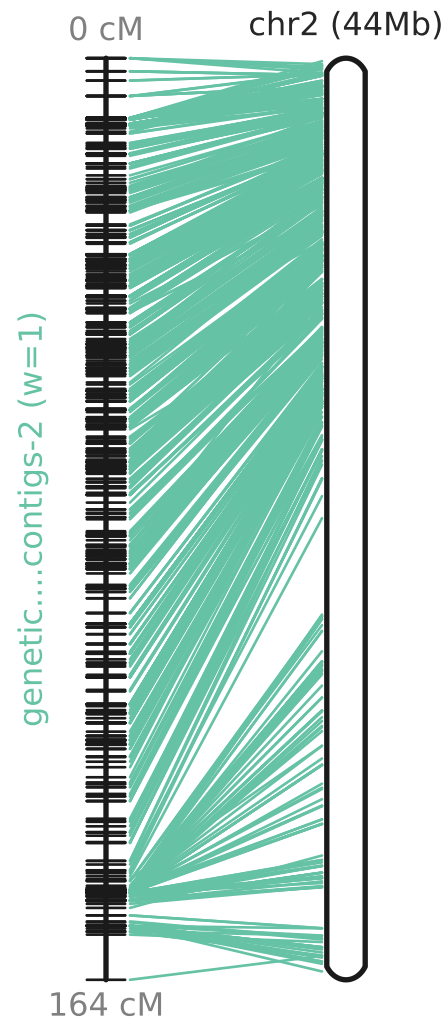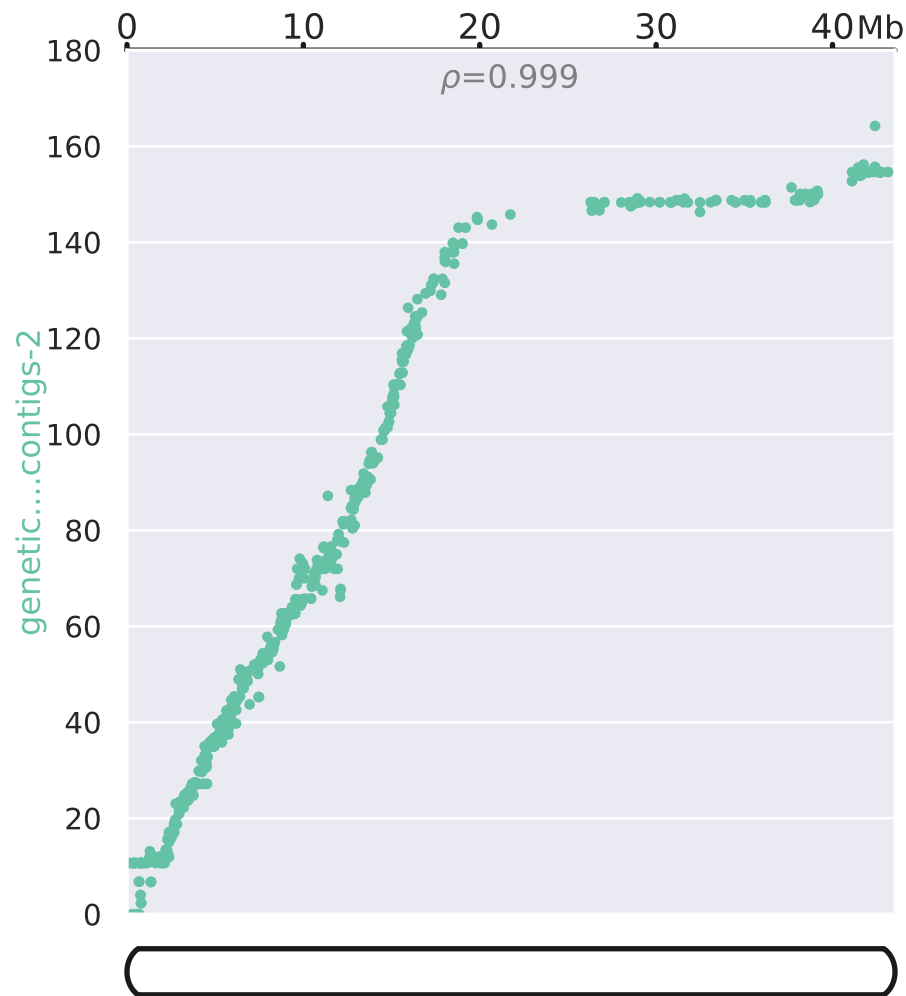

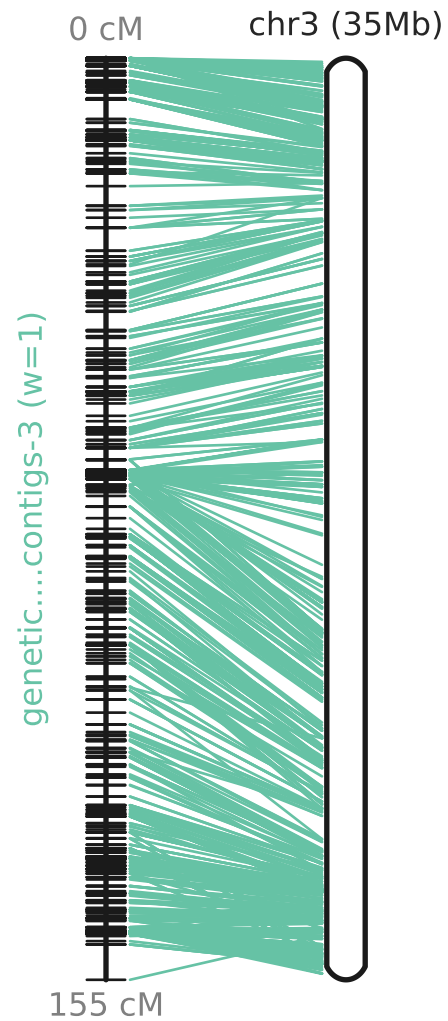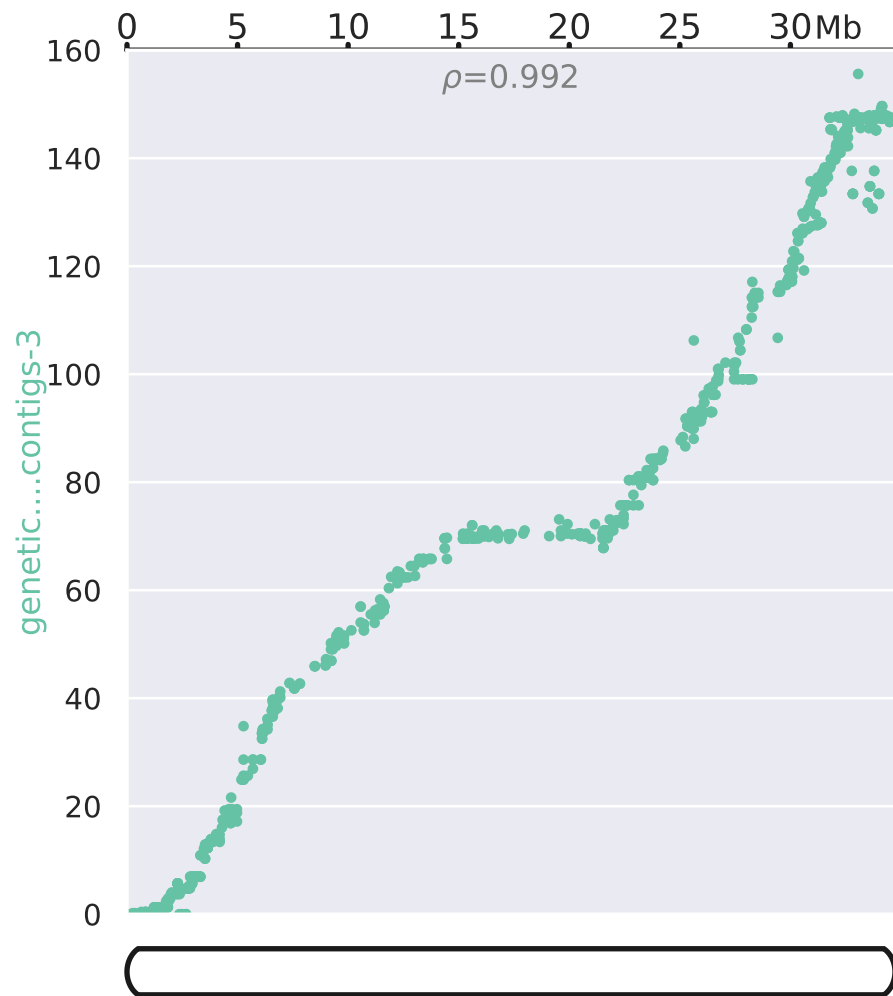

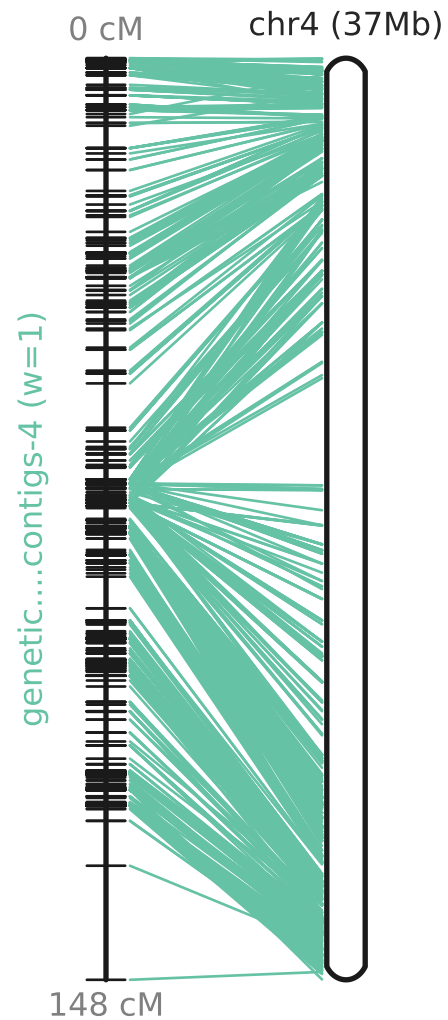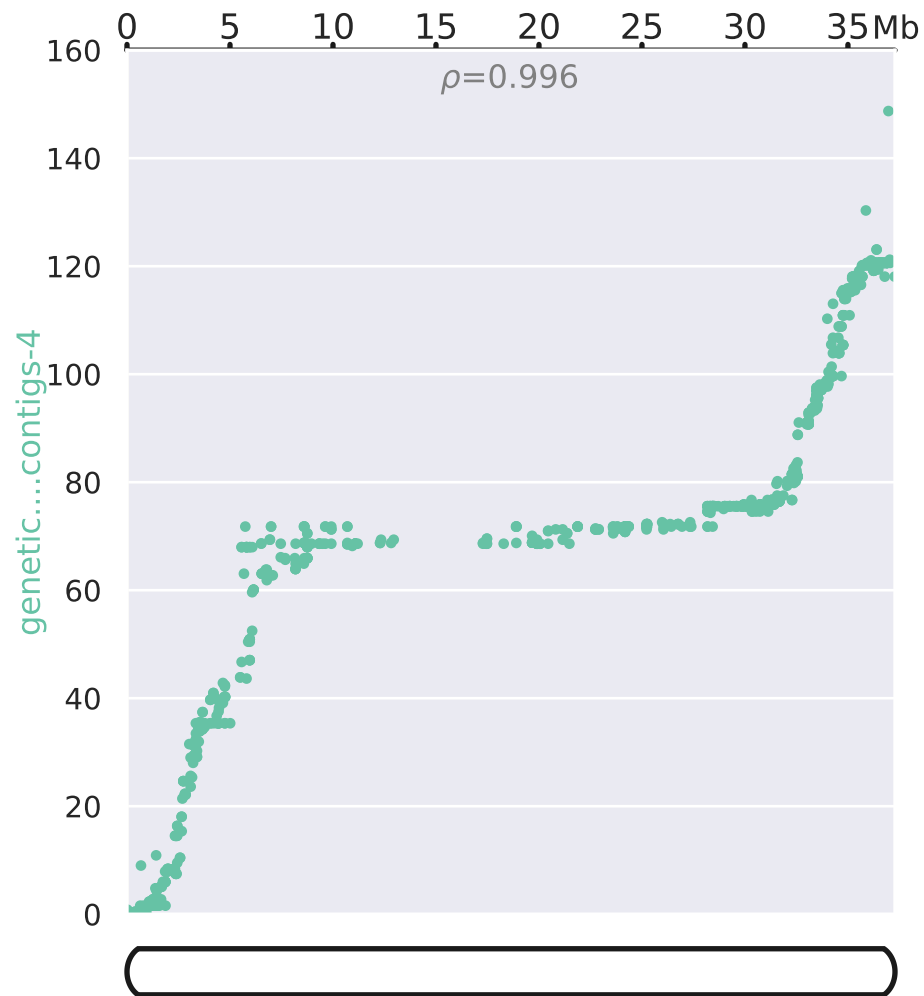

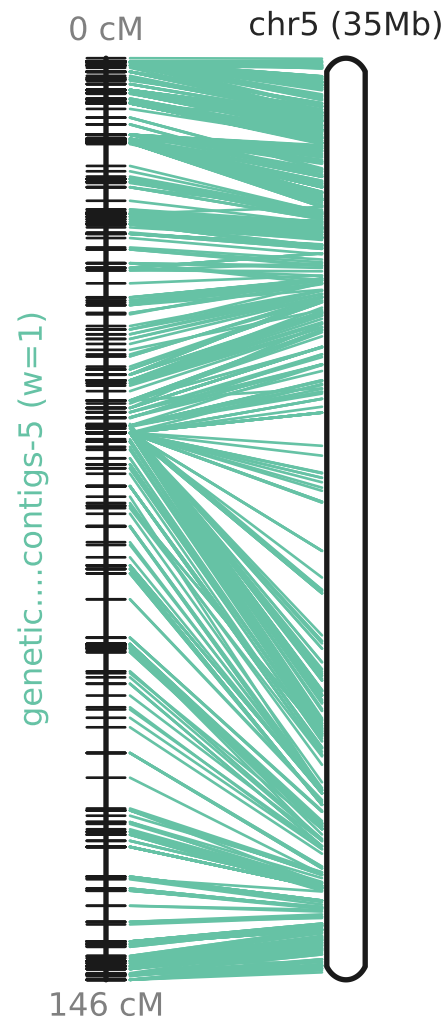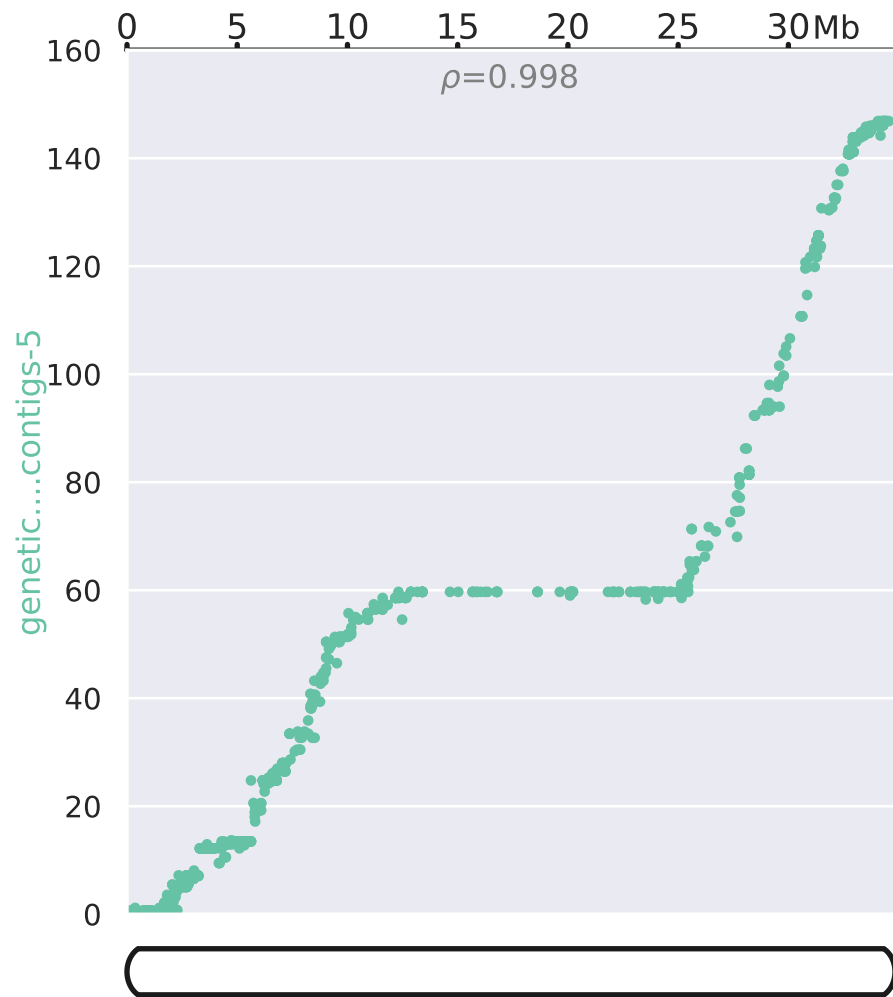

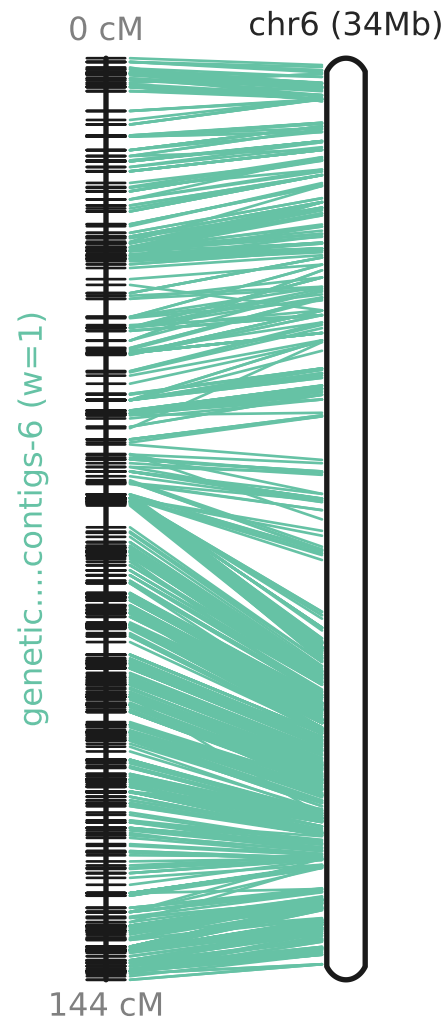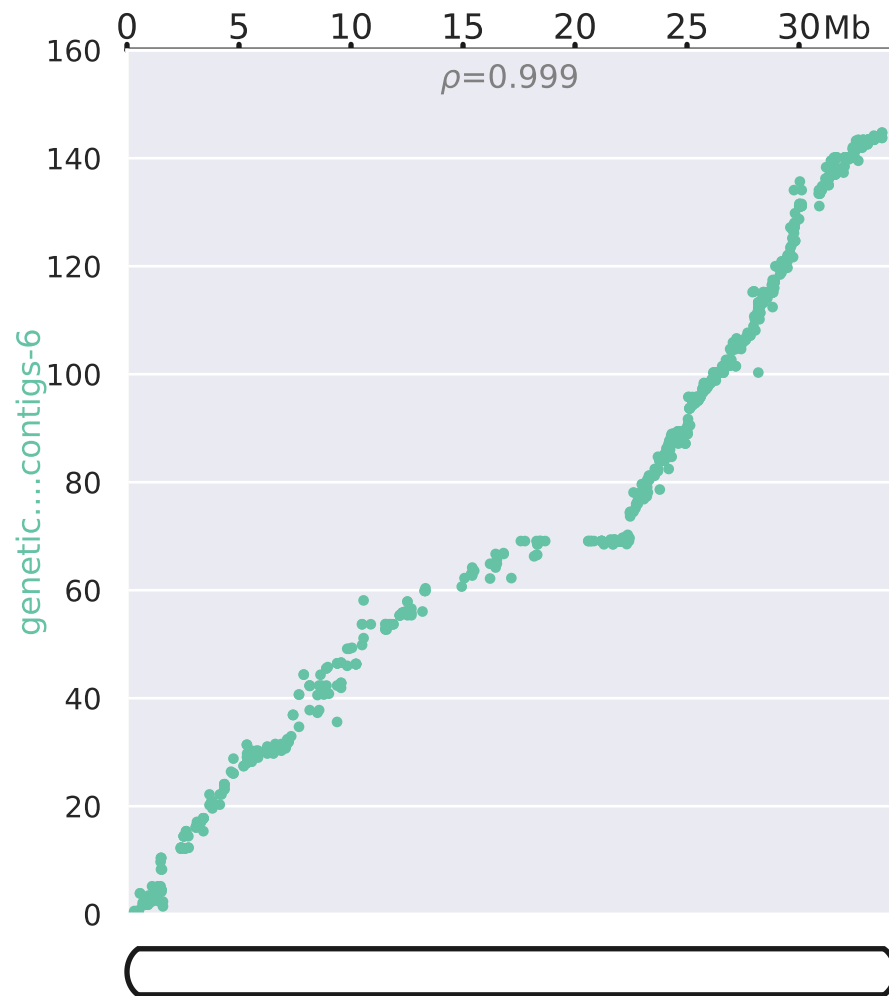

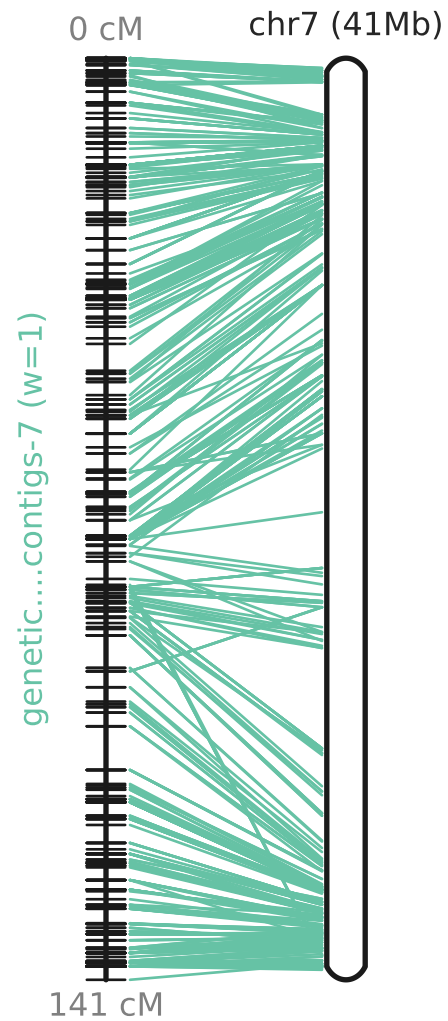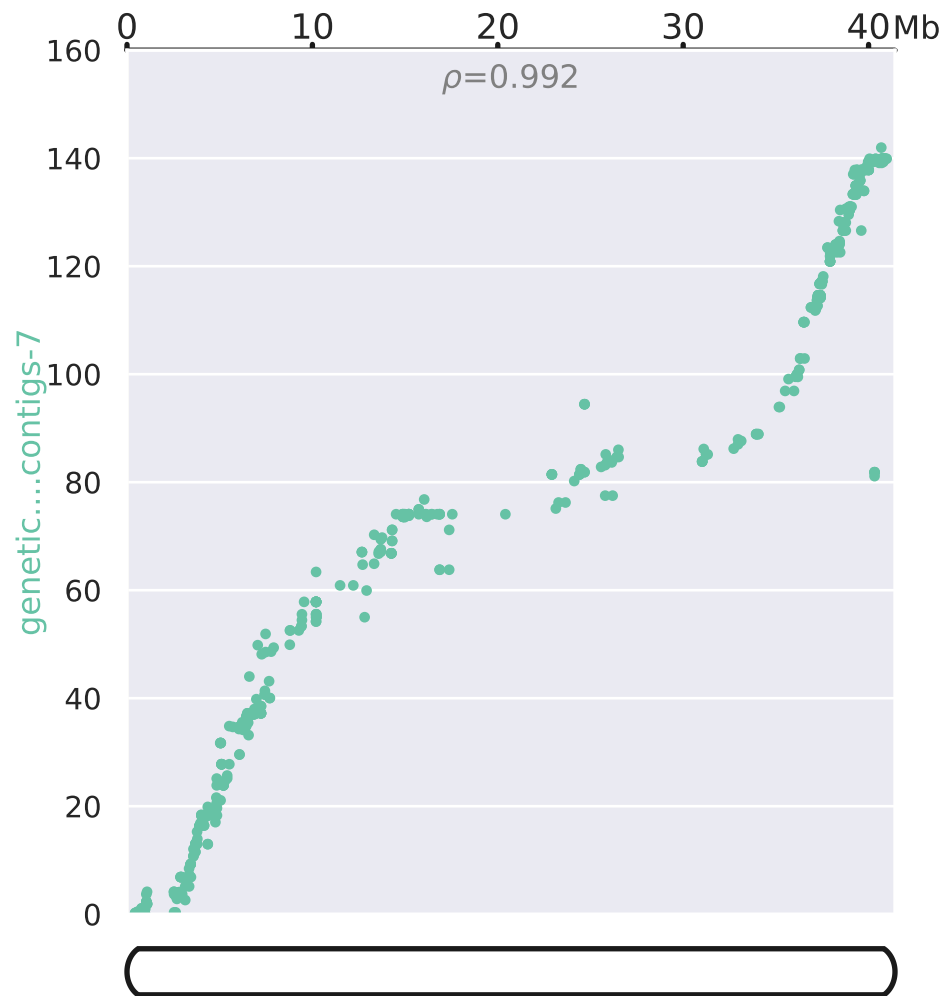

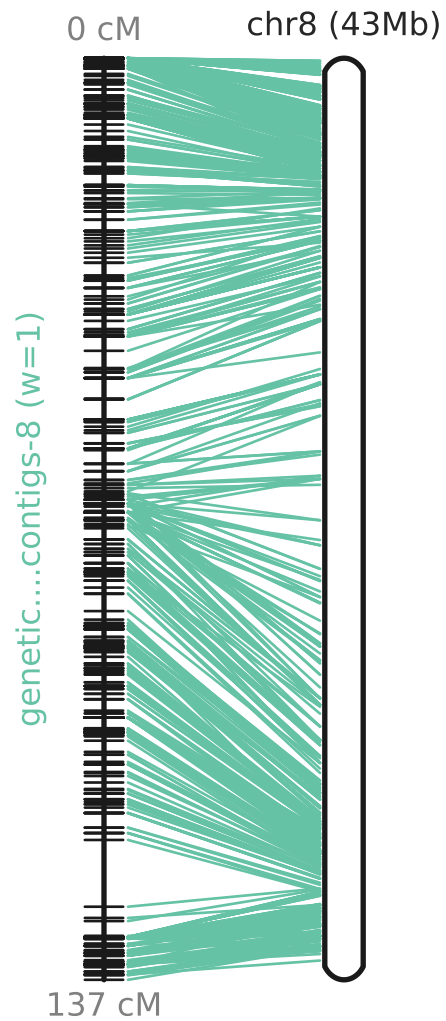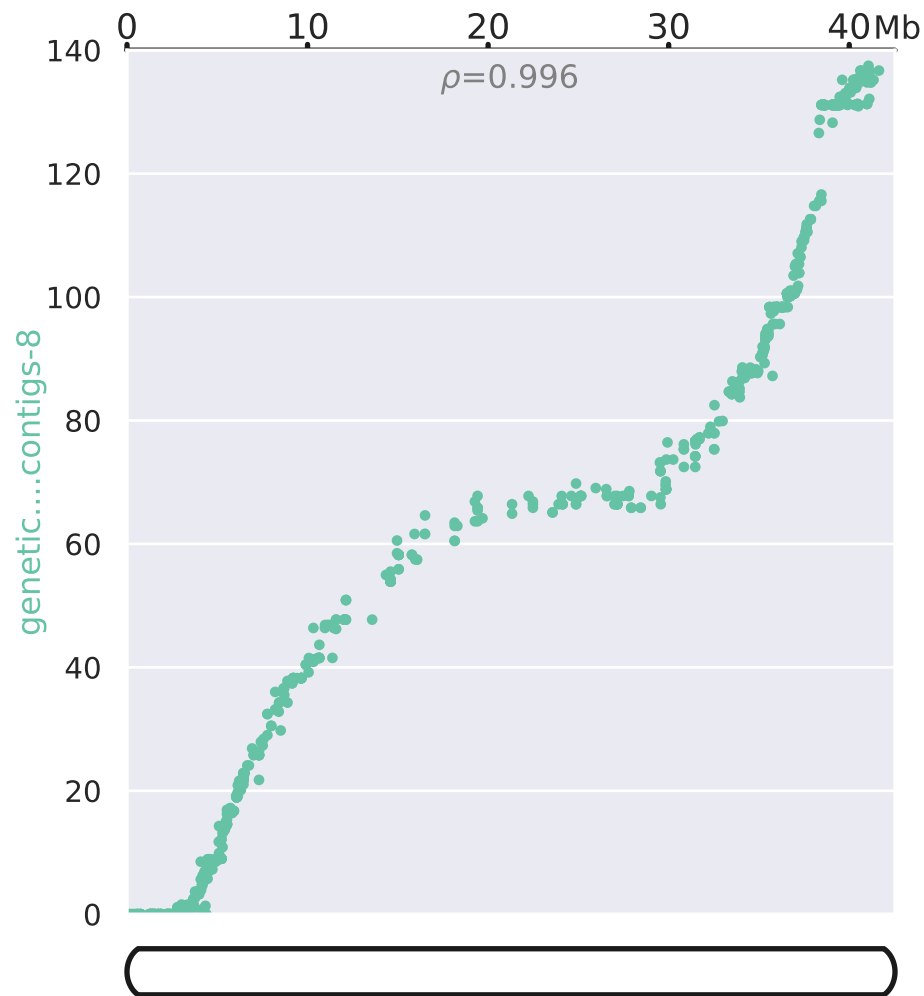

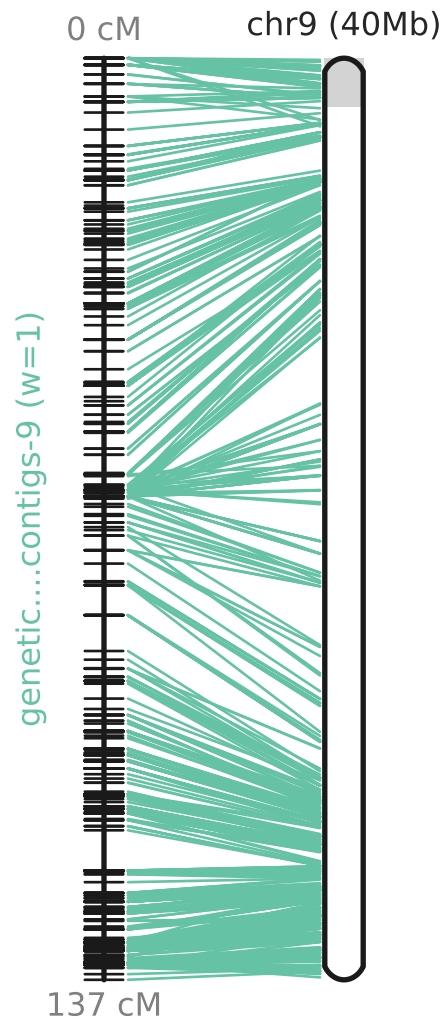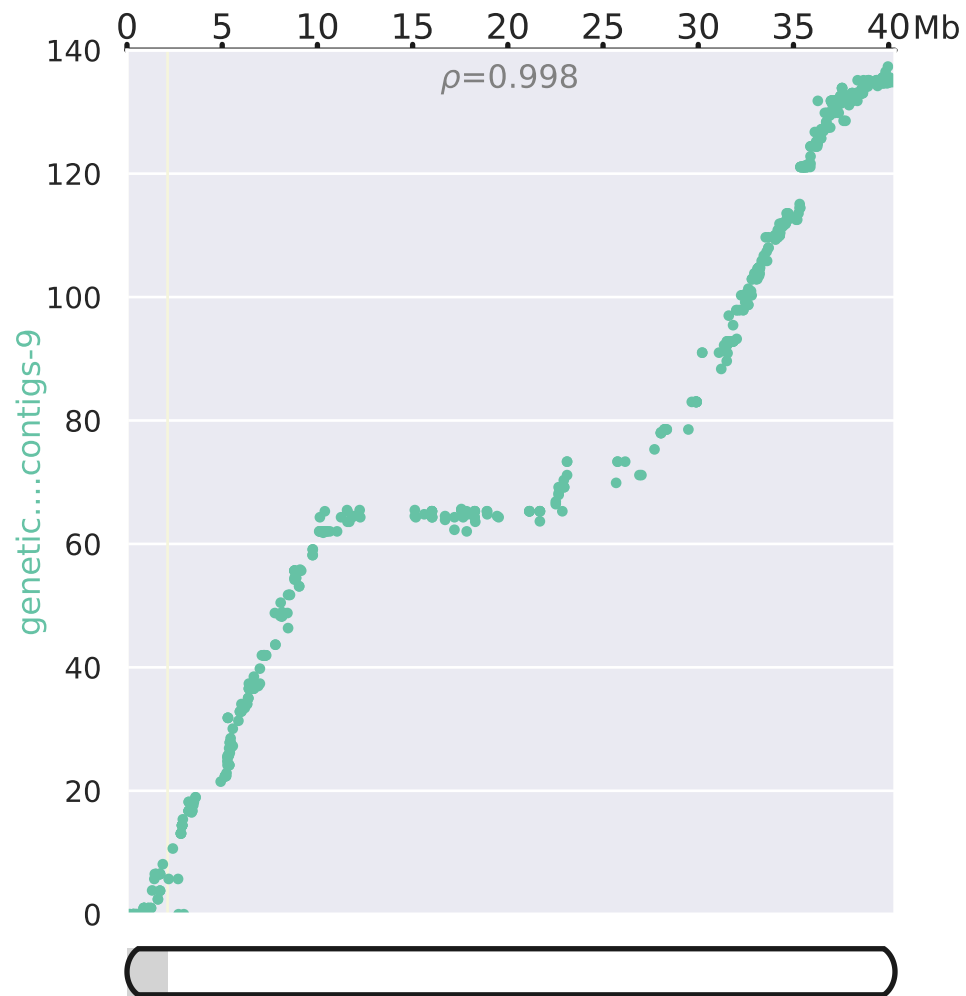

Supplement: jkaf083_Supplementary_Data [file jkaf083_supplementary_data.zip › Additional_File_1_G3-2024-405442.pdf]

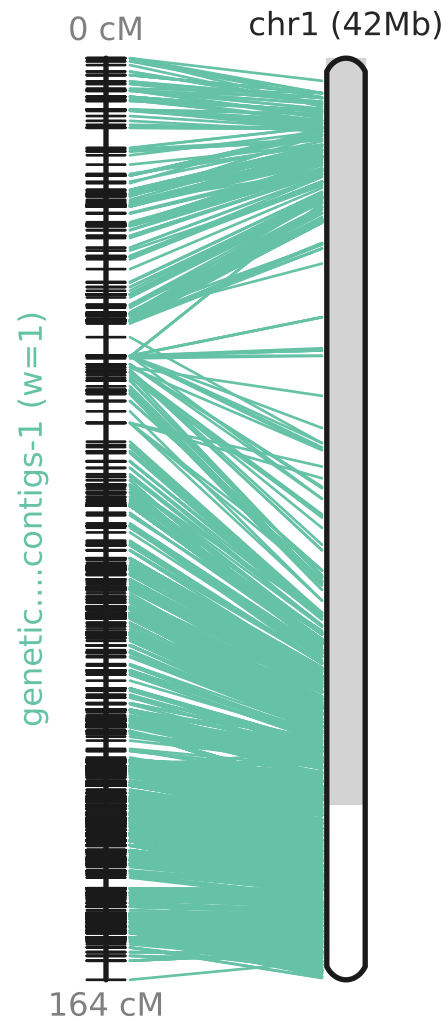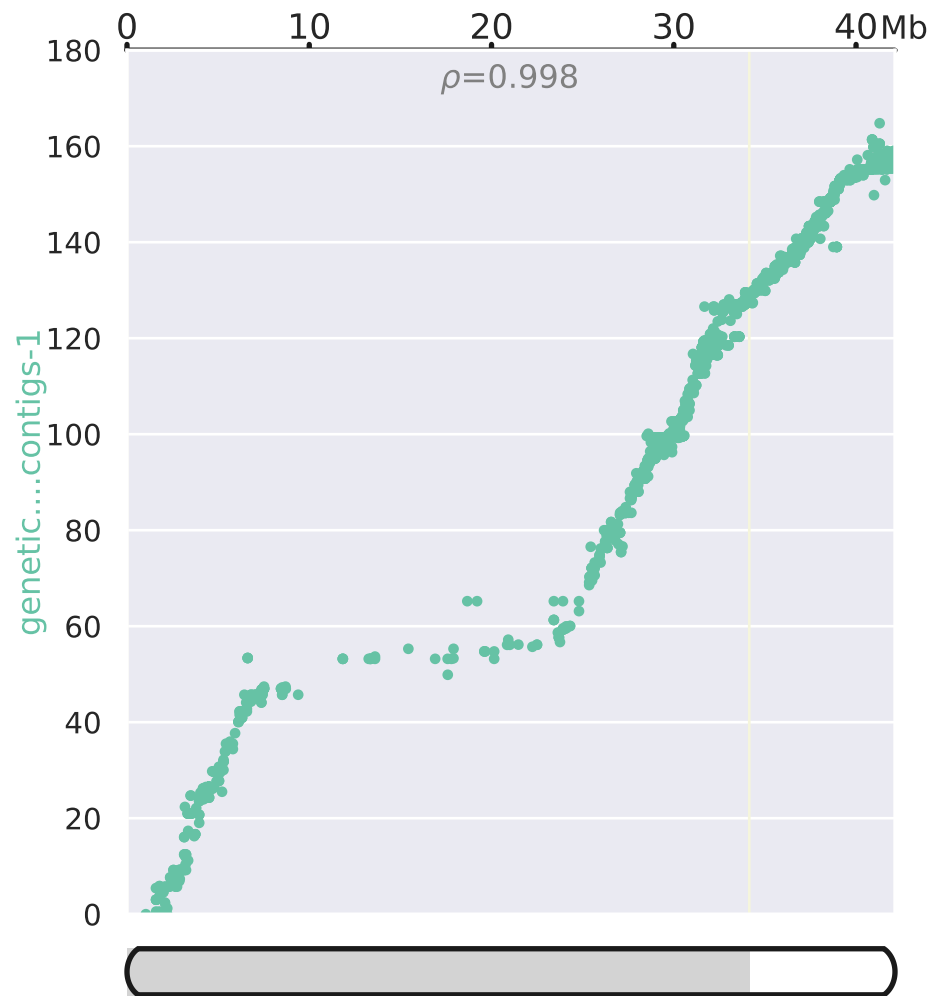

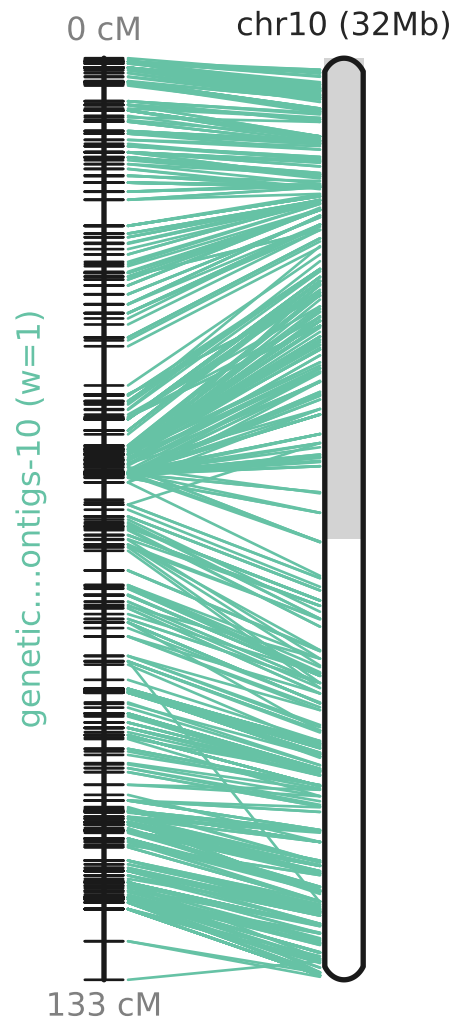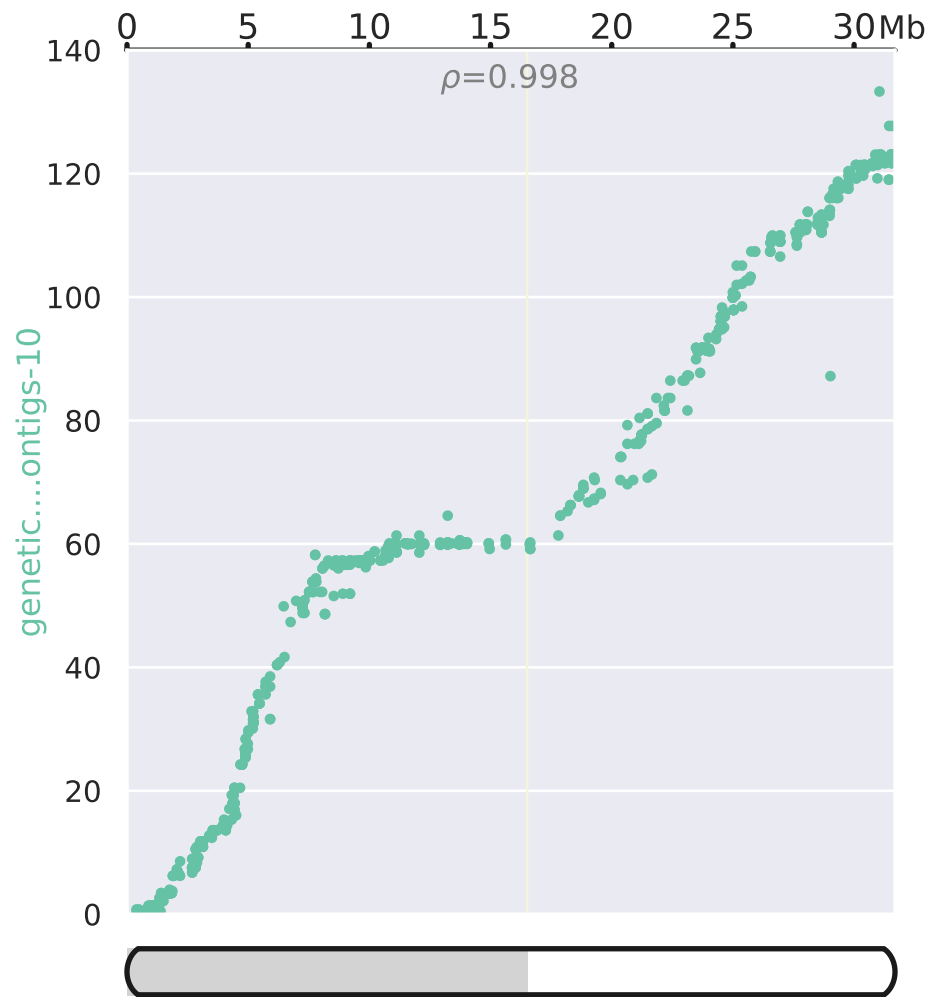

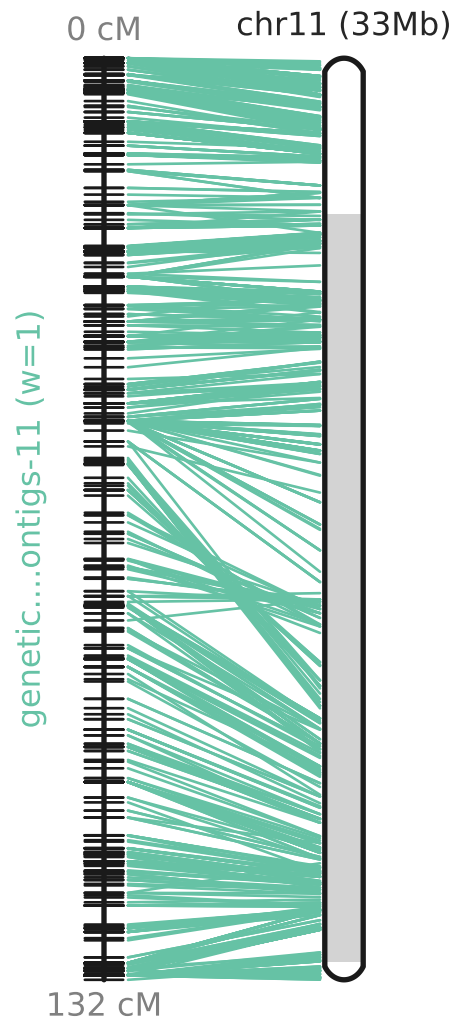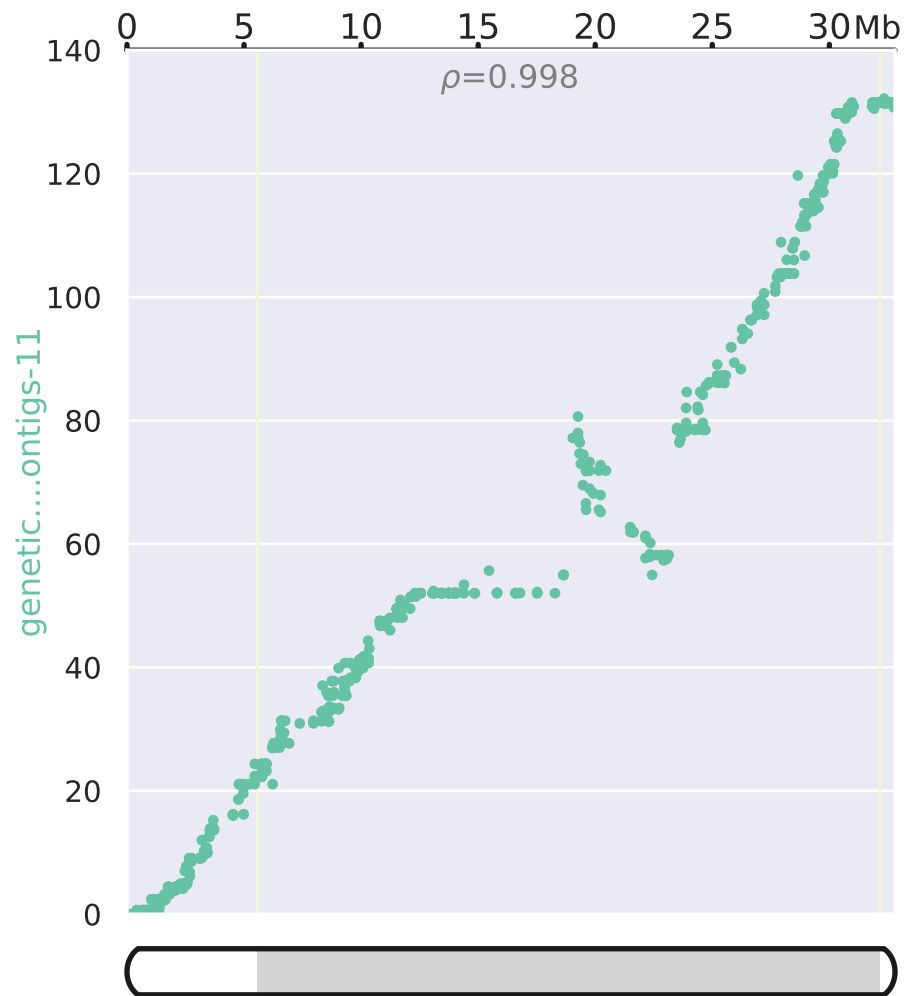

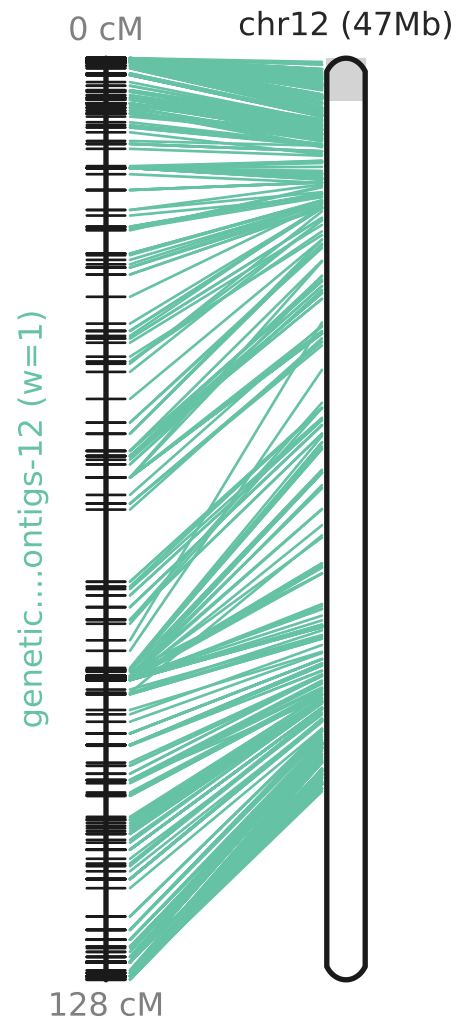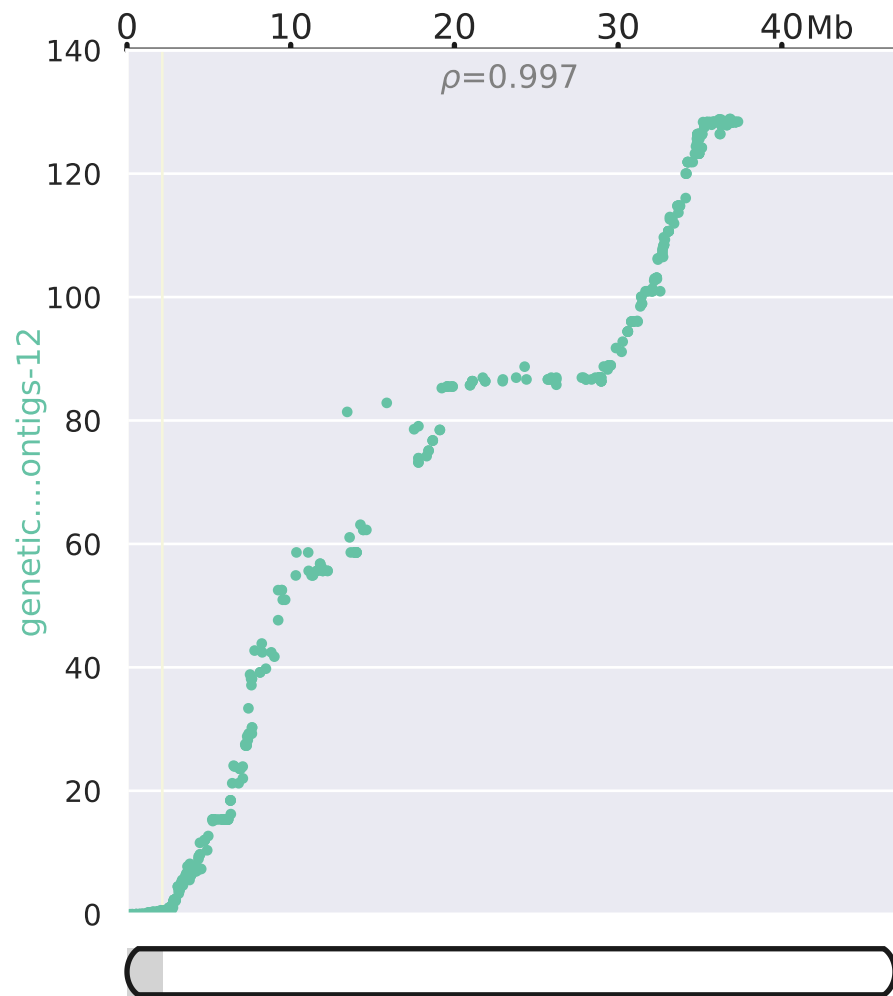

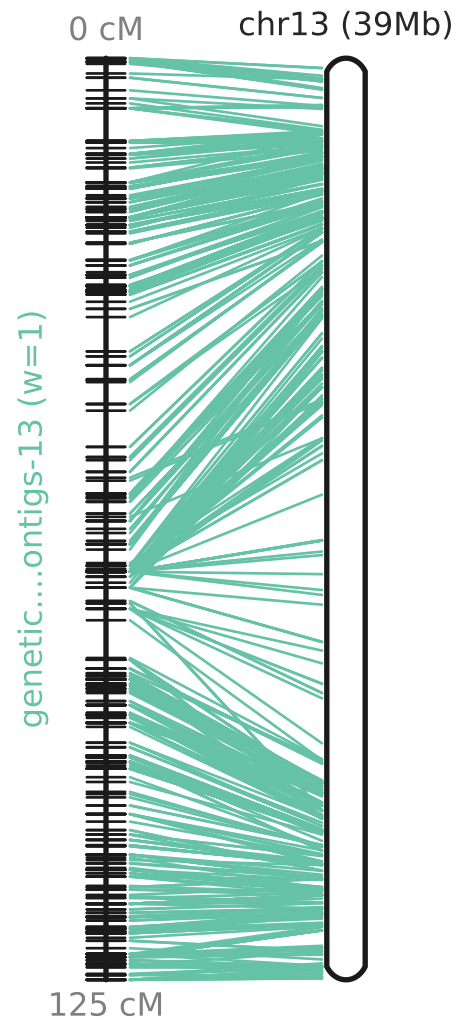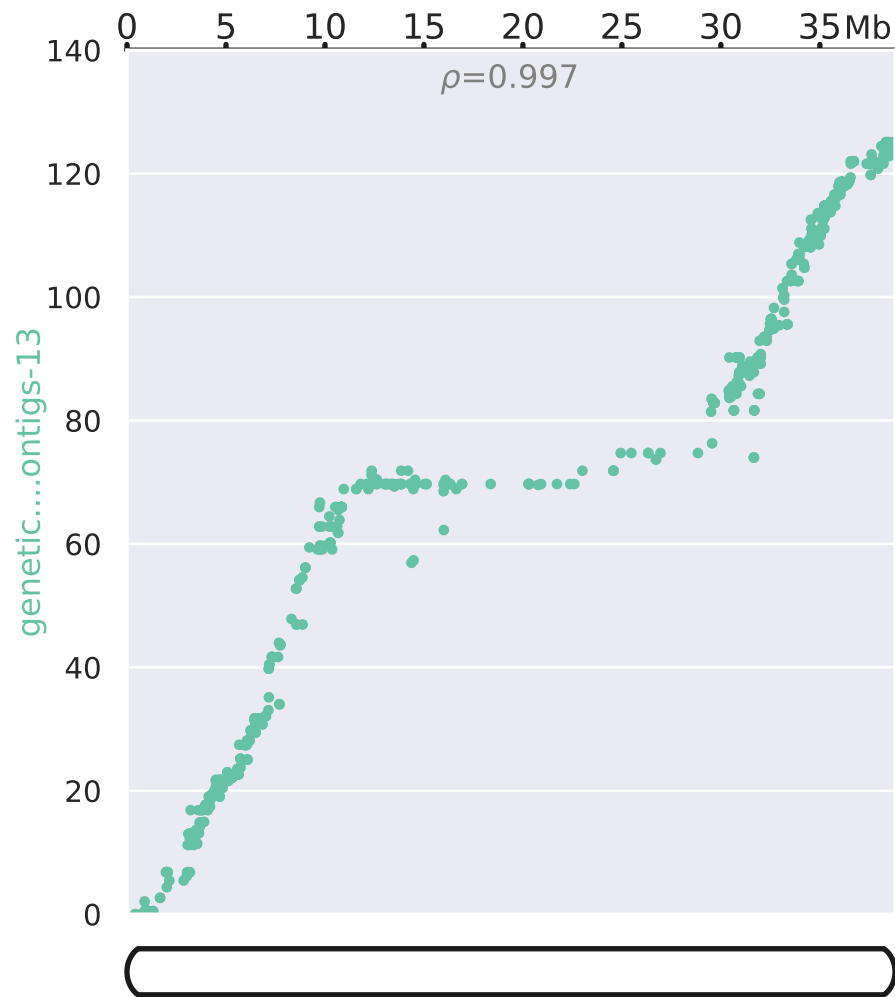

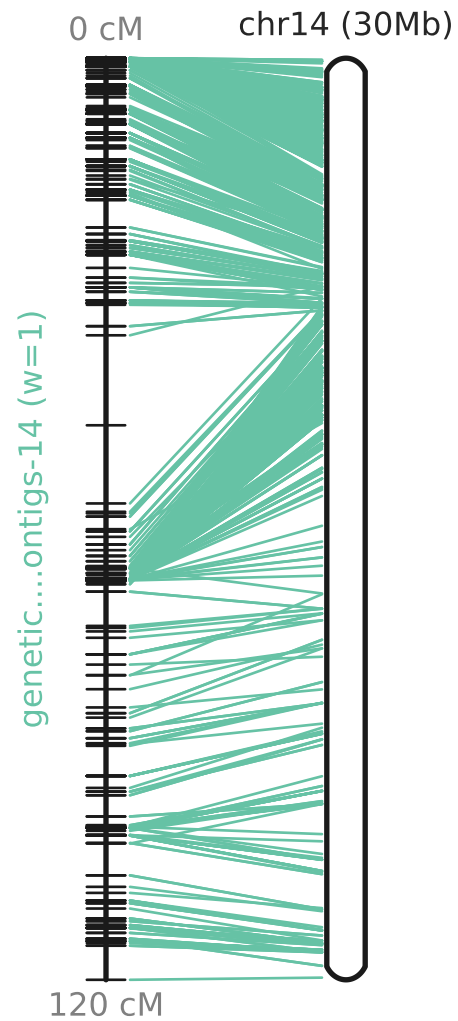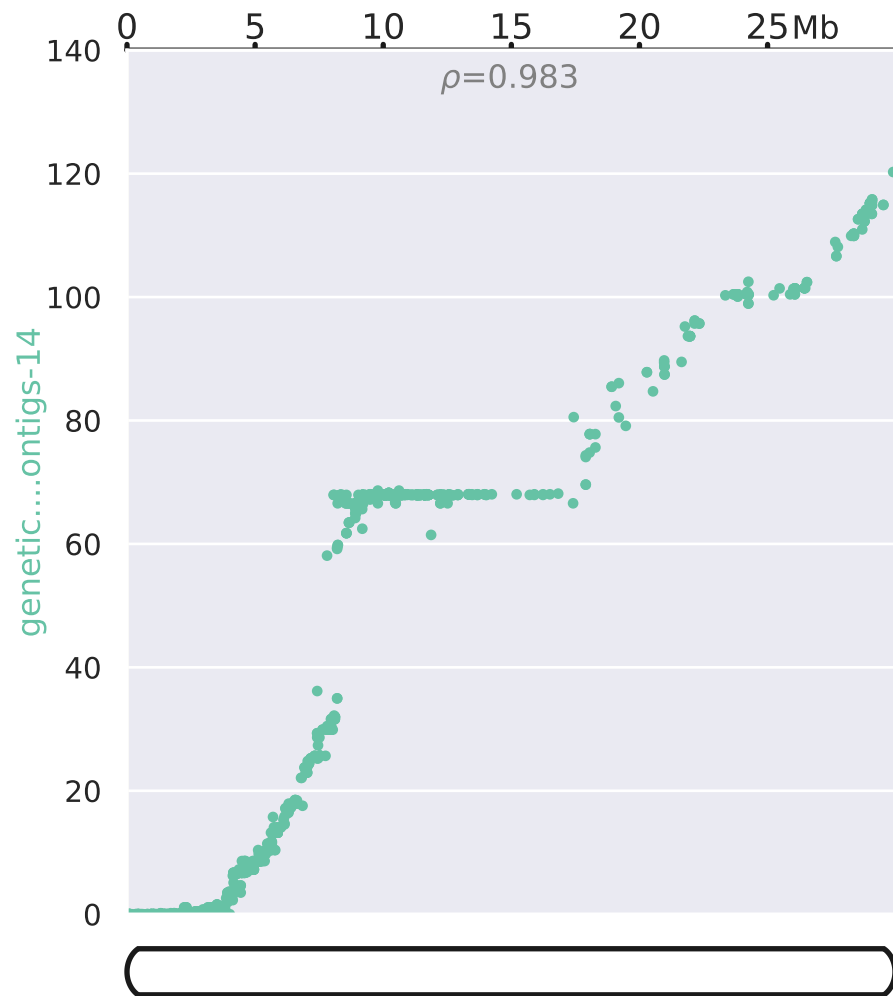

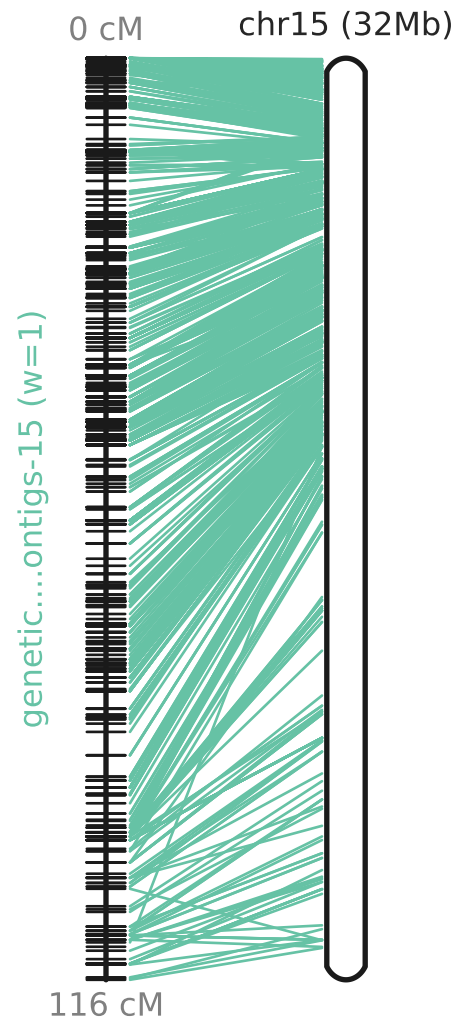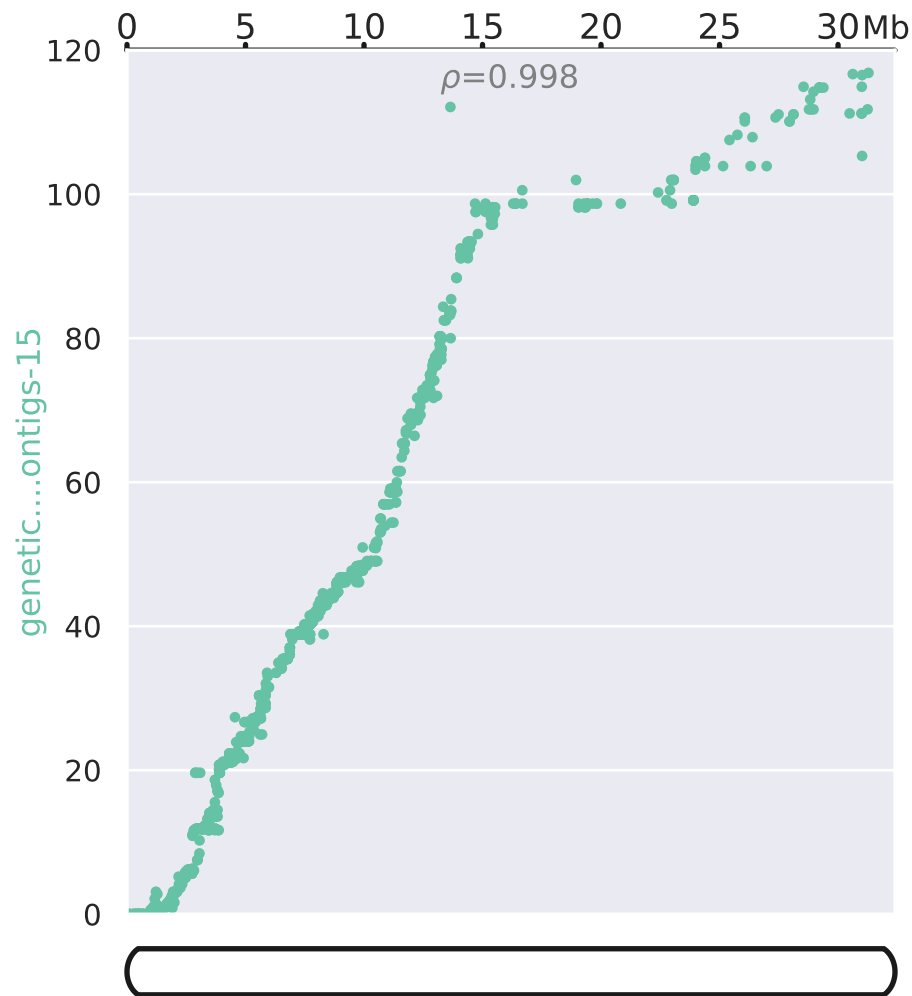

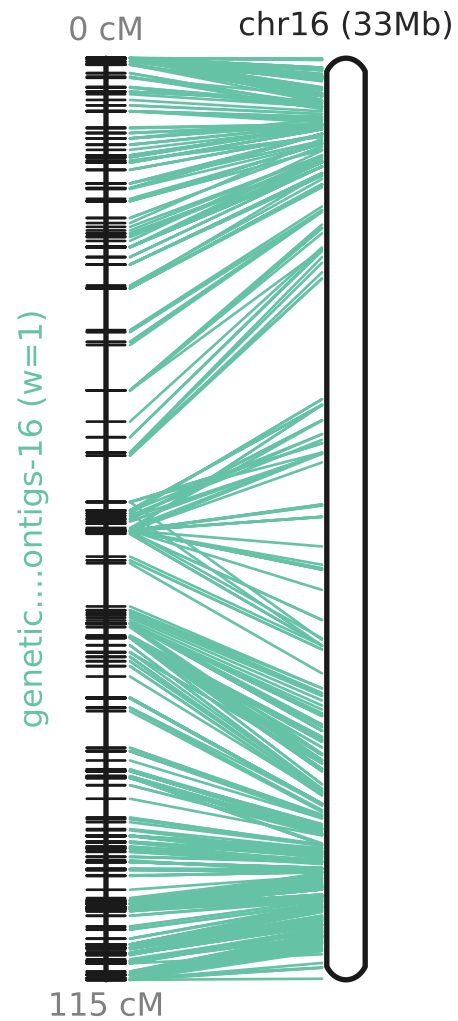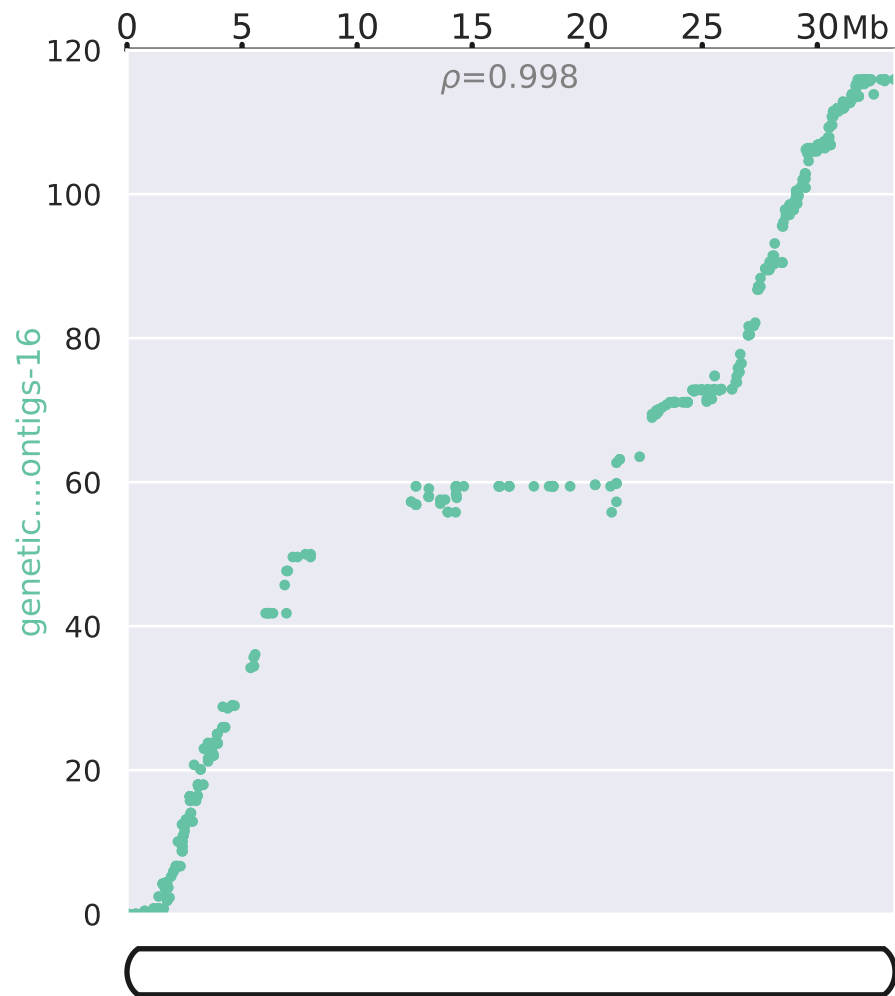

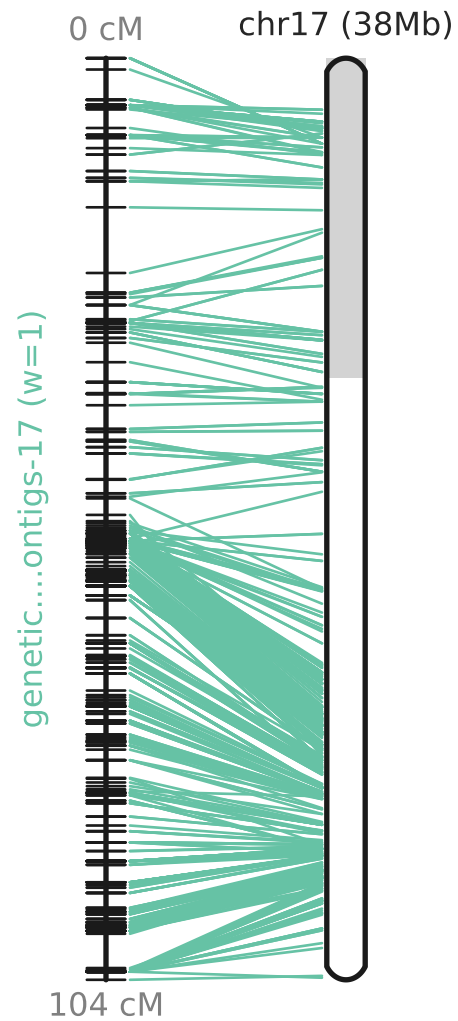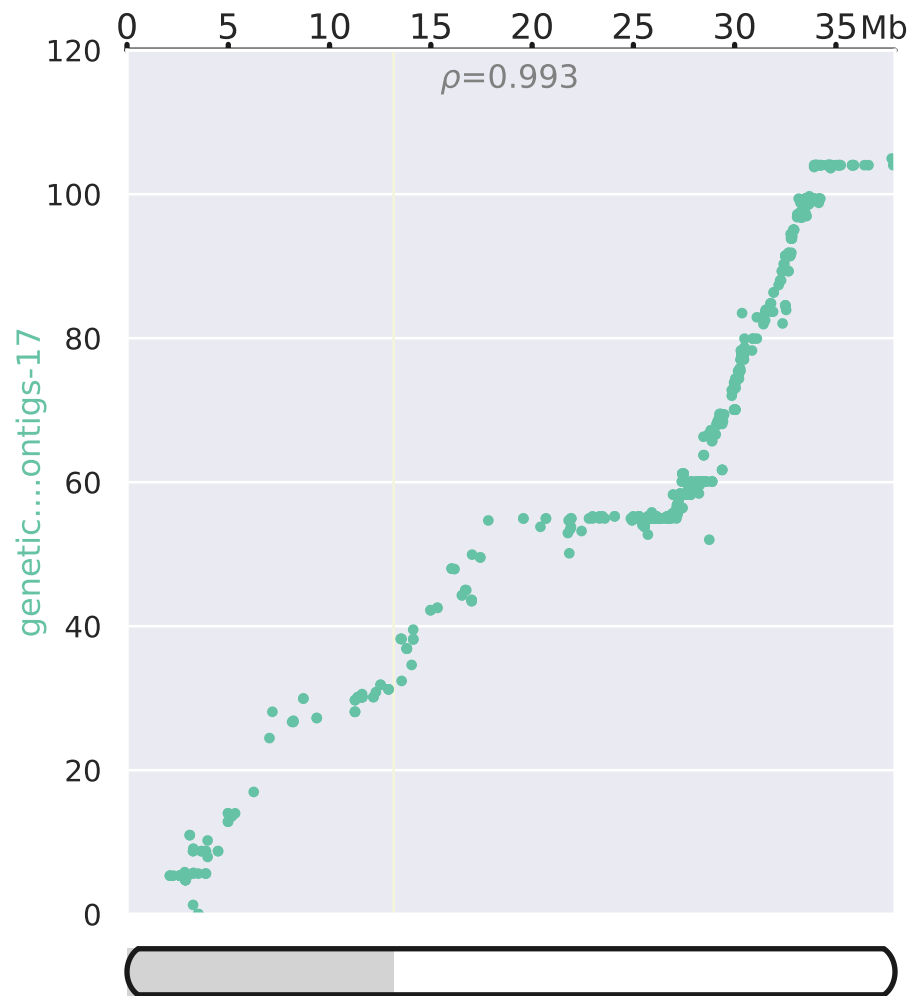

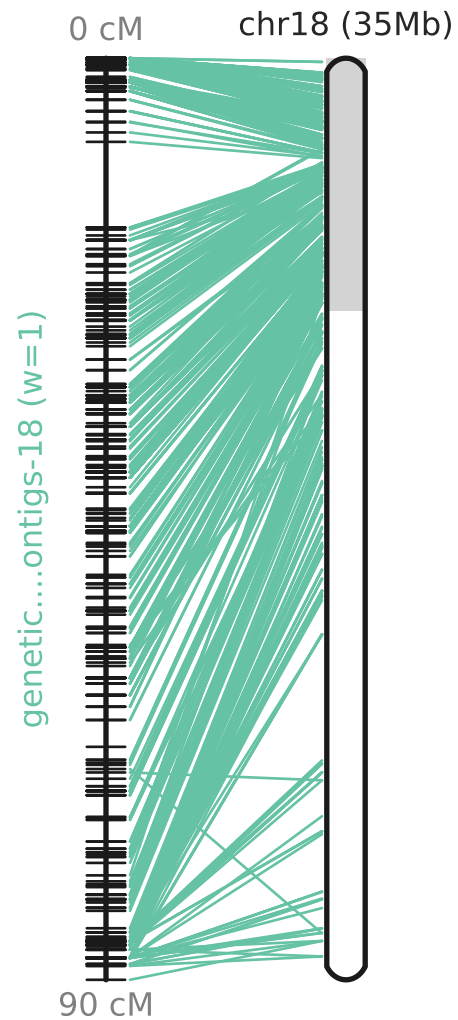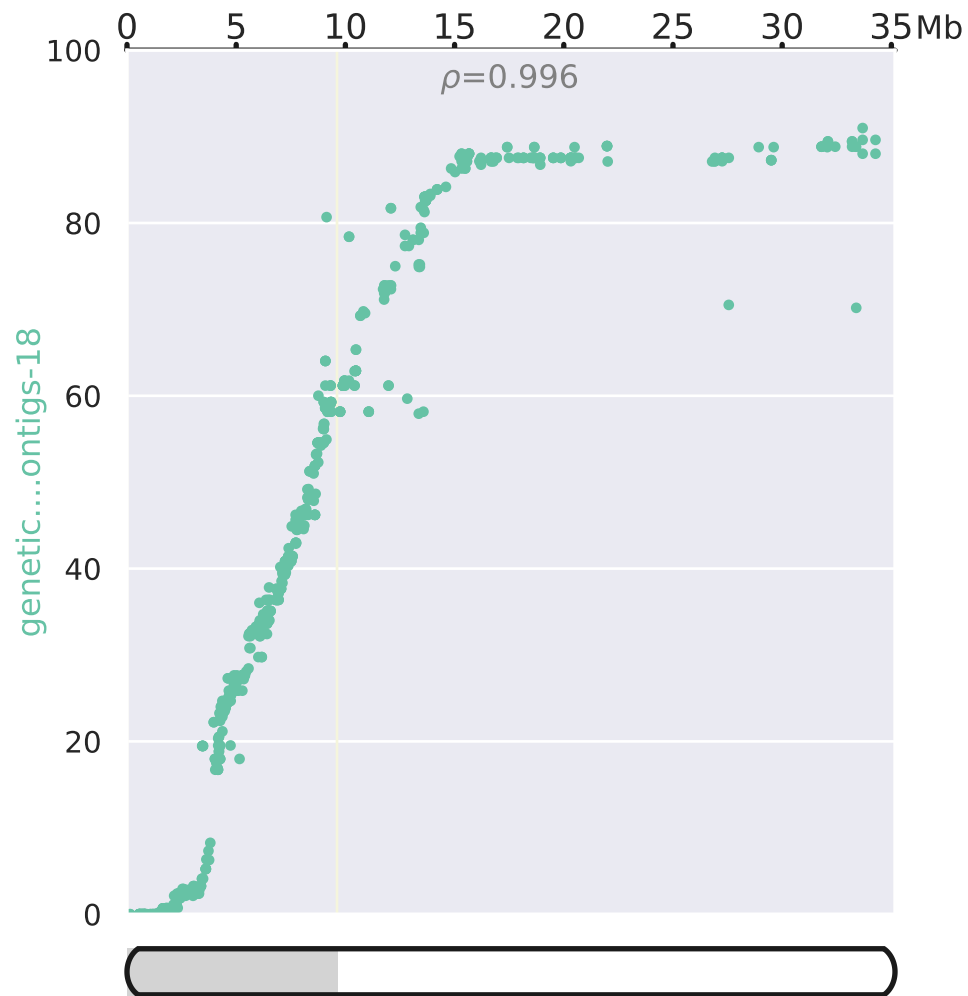

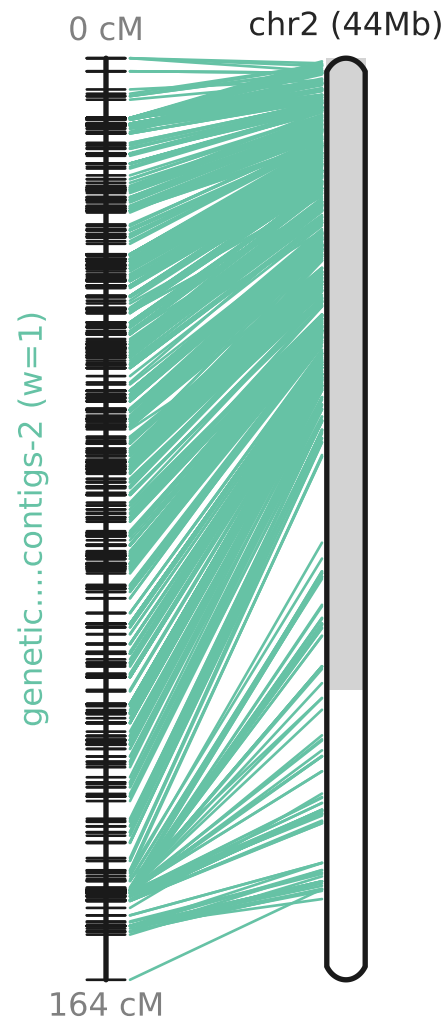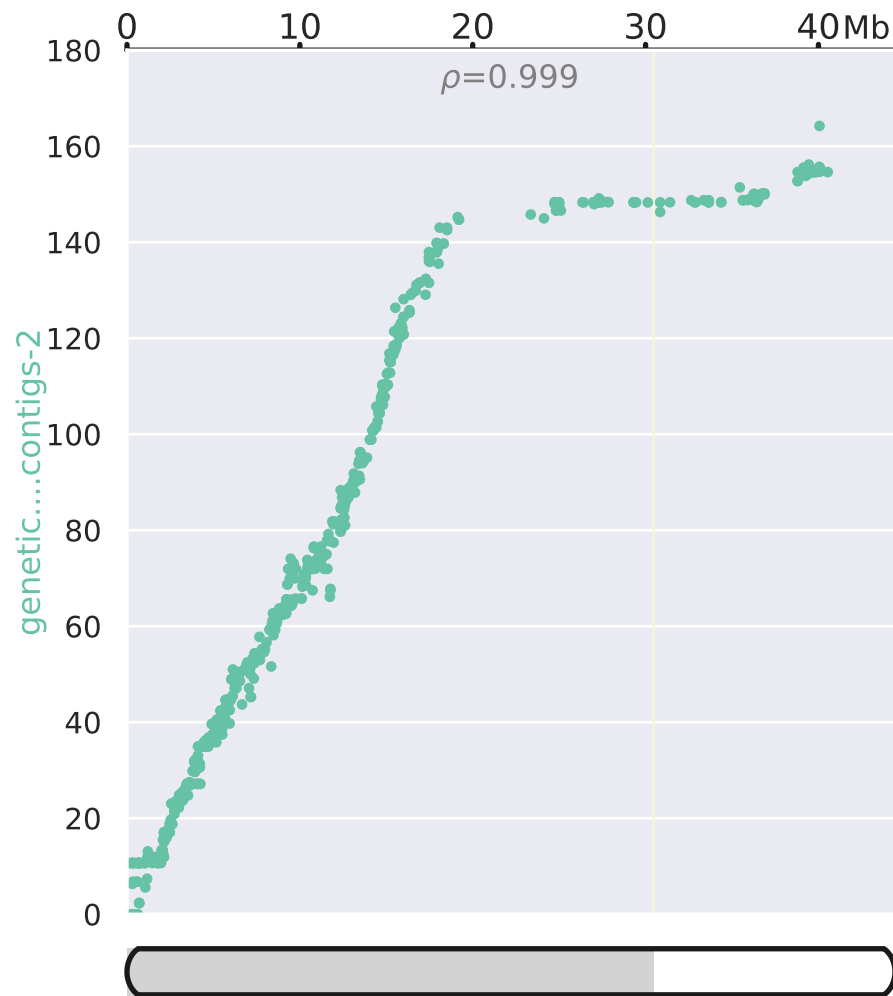

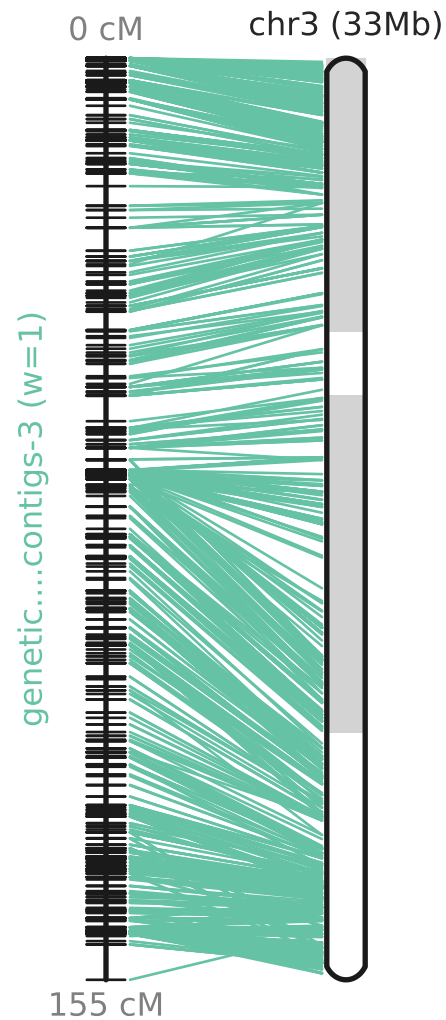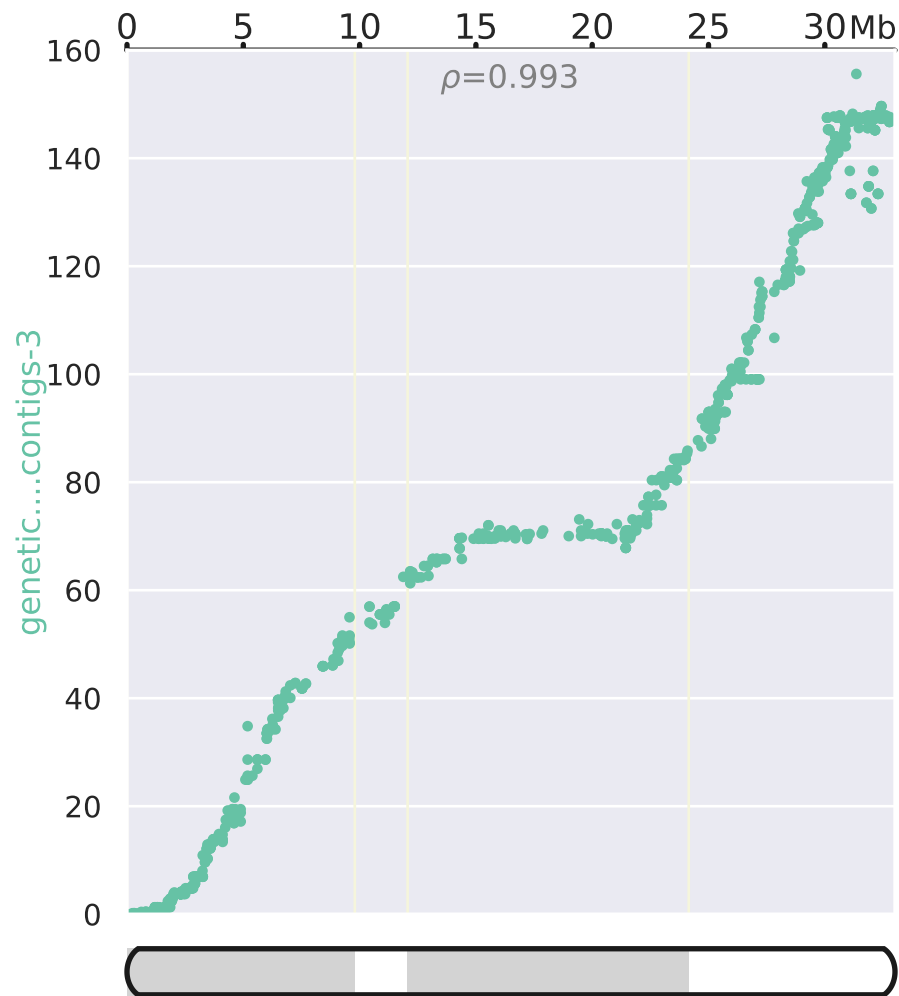

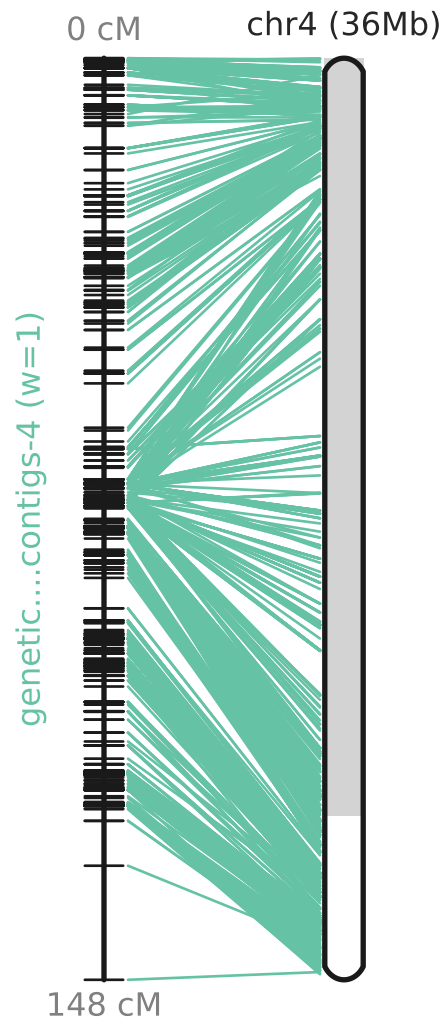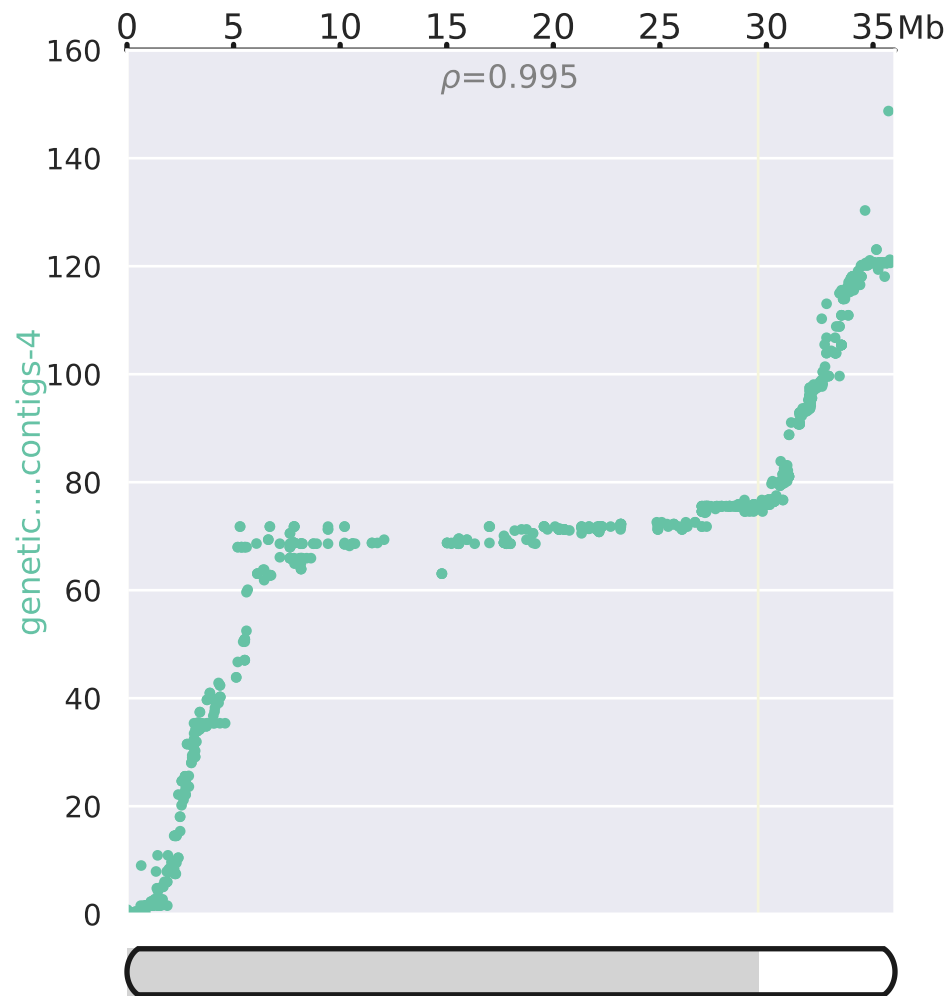

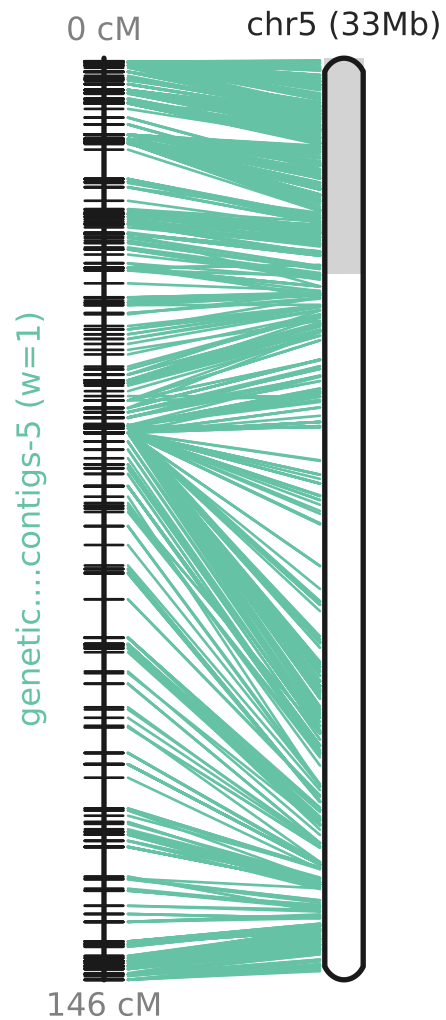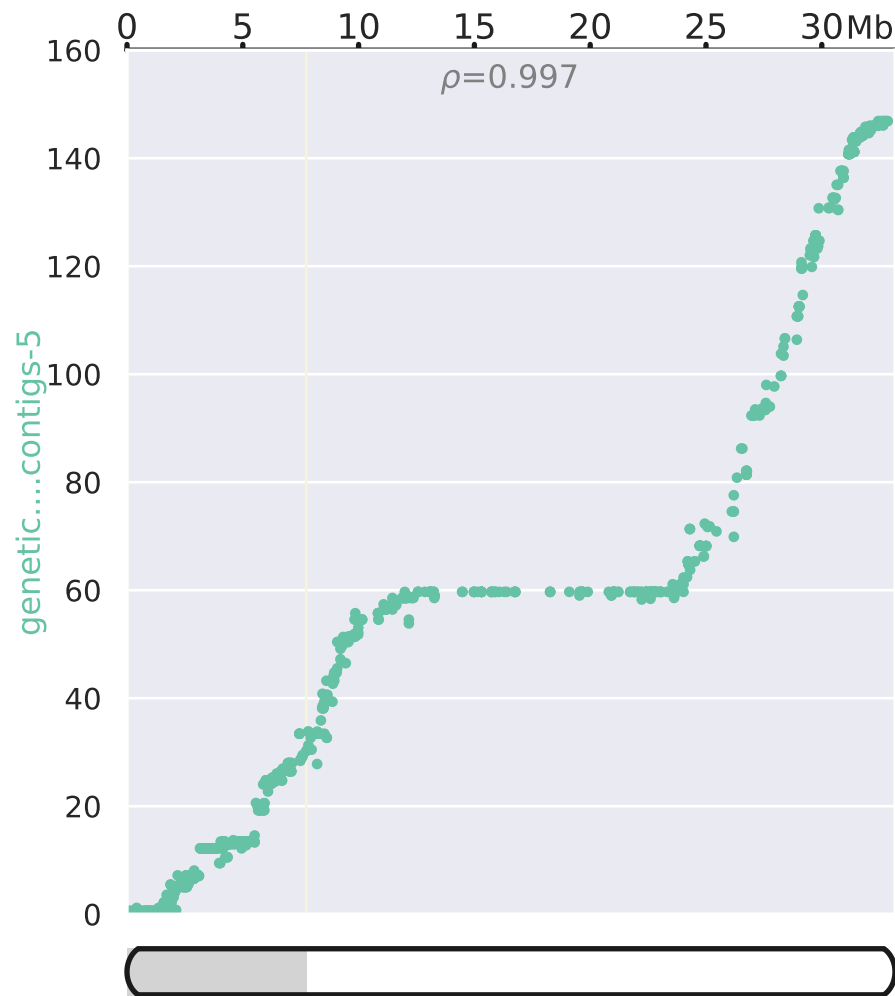

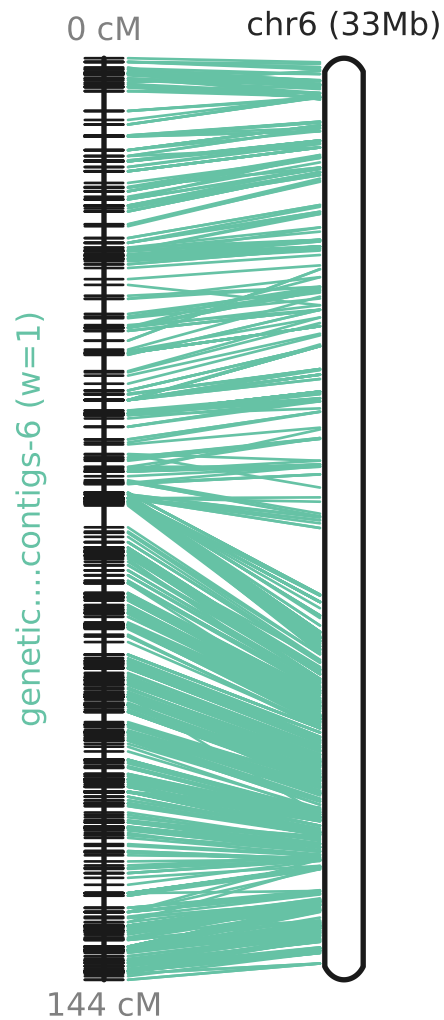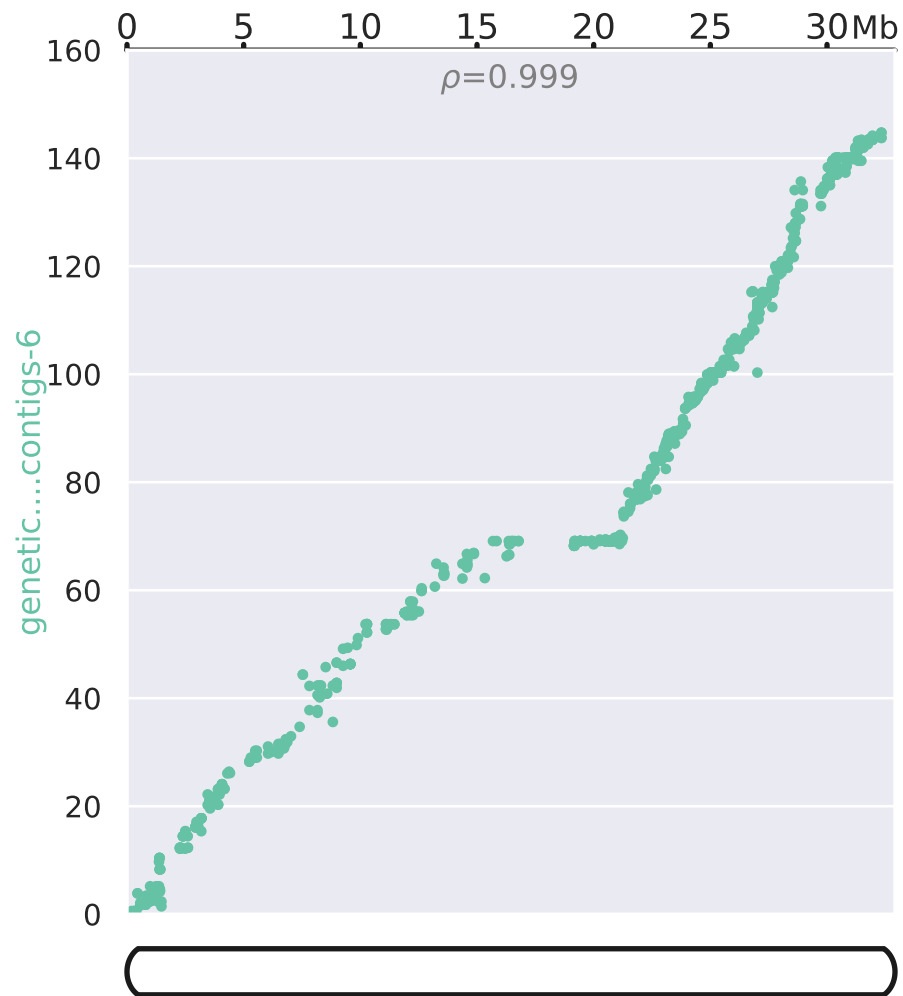

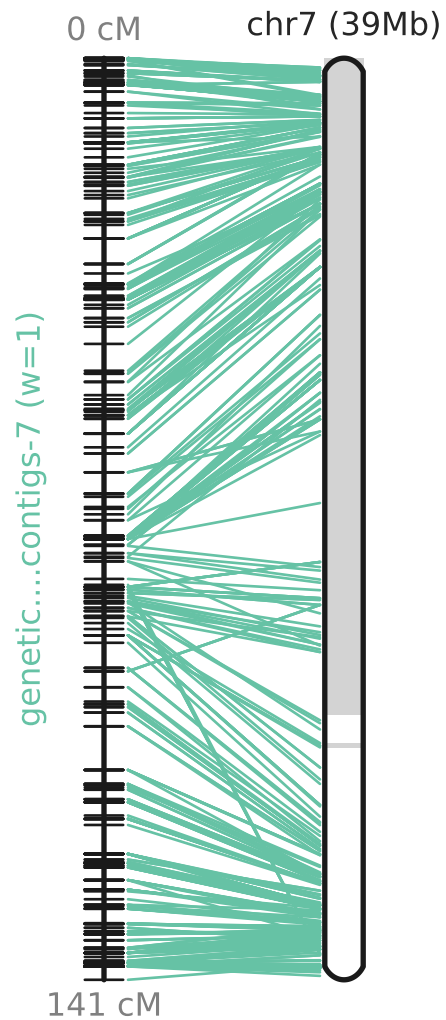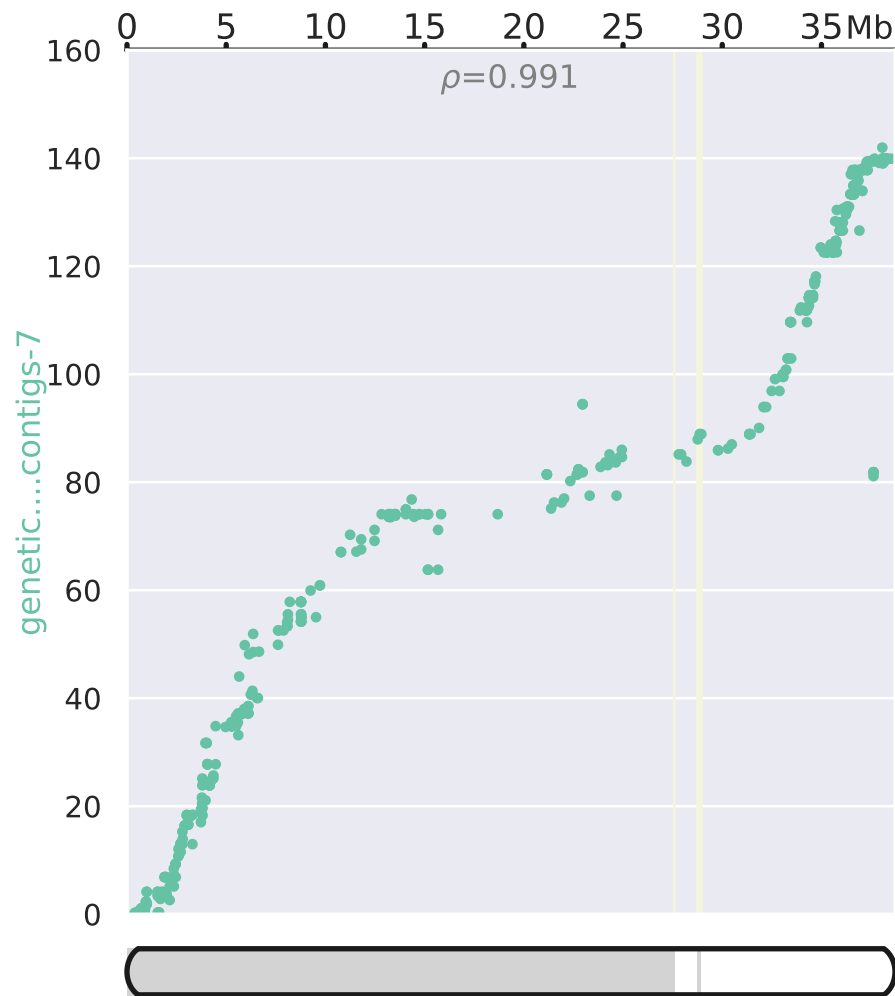

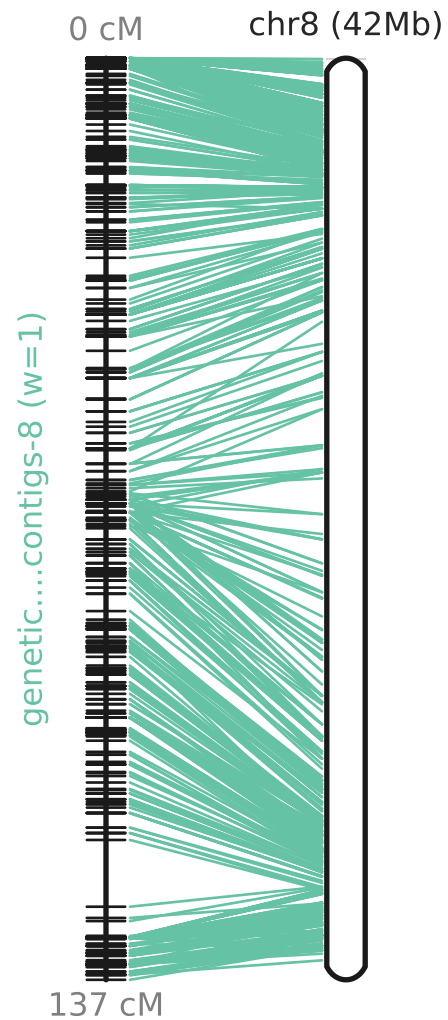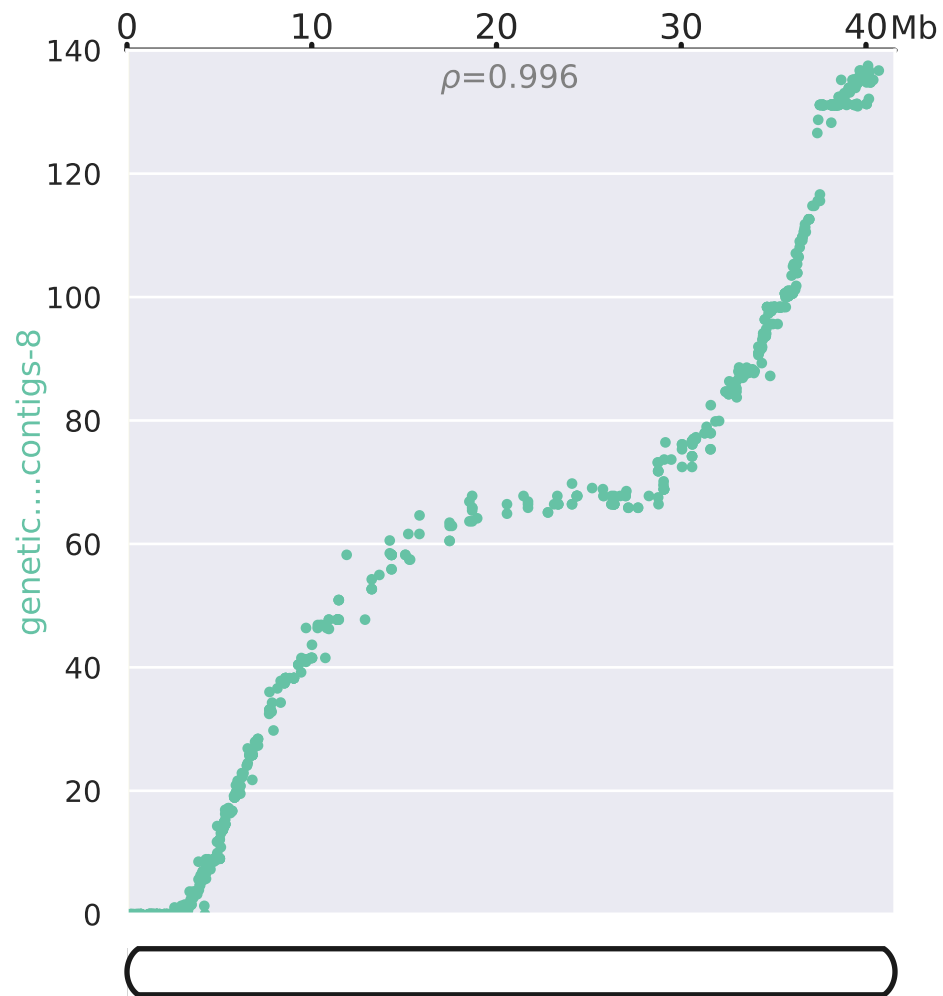

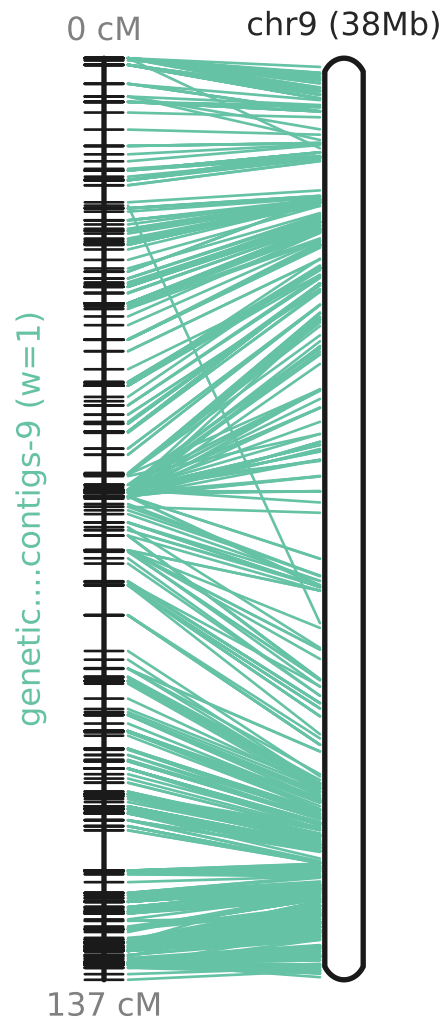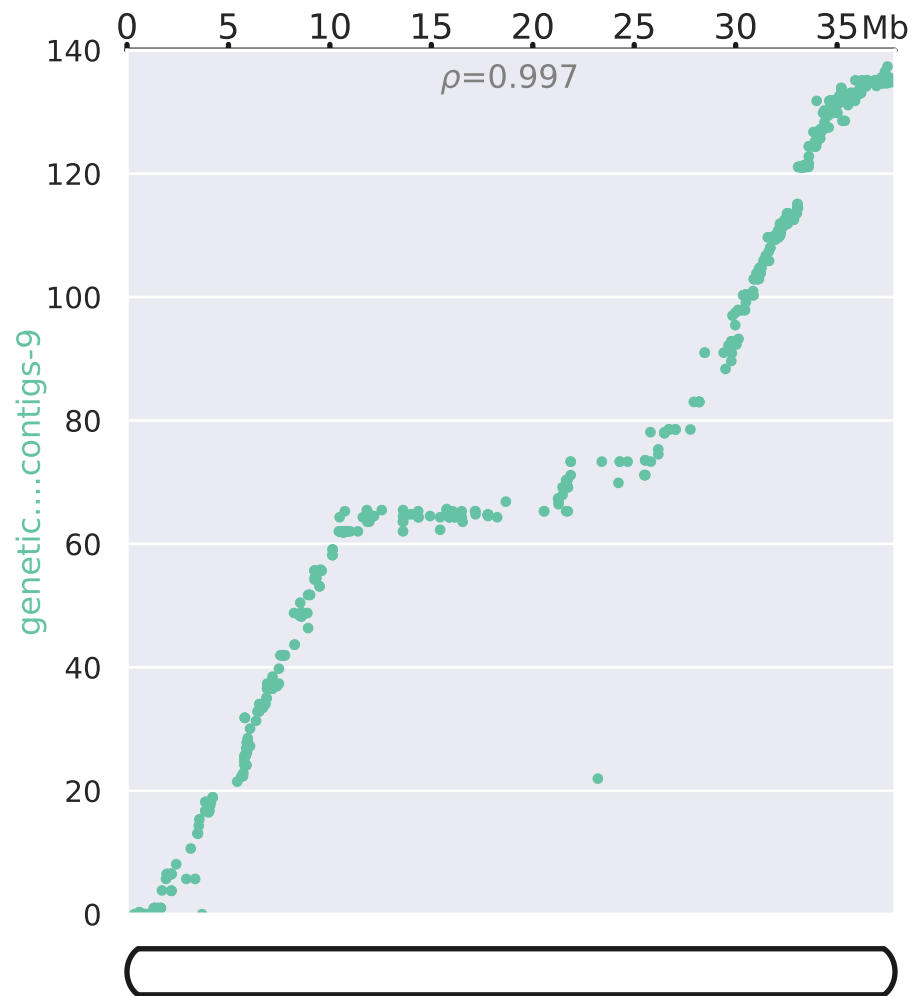

Supplement: jkaf083_Supplementary_Data [file jkaf083_supplementary_data.zip › Additional_File_2_G3-2024-405442.pdf]
